# FoldPAthreader: predicting protein folding pathway using a novel folding force field model derived from known protein universe

**DOI:** 10.1186/s13059-024-03291-x

**Published:** 2024-06-11

**Authors:** Kailong Zhao, Pengxin Zhao, Suhui Wang, Yuhao Xia, Guijun Zhang

**Affiliations:** College of Information Engineering, Zhejiang University of Technology, HangZhou, 310023 China

**Keywords:** Protein folding pathway, Folding force field, Evolutionary history

## Abstract

Protein folding has become a tractable problem with the significant advances in deep learning-driven protein structure prediction. Here we propose FoldPAthreader, a protein folding pathway prediction method that uses a novel folding force field model by exploring the intrinsic relationship between protein evolution and folding from the known protein universe. Further, the folding force field is used to guide Monte Carlo conformational sampling, driving the protein chain fold into its native state by exploring potential intermediates. On 30 example targets, FoldPAthreader successfully predicts 70% of the proteins whose folding pathway is consistent with biological experimental data.

**Supplementary Information:**

The online version contains supplementary material available at 10.1186/s13059-024-03291-x.

## Background

The folding process of proteins reveals fundamental principles of life [1]. Proper folding typically results in proteins existing in a soluble form within cells. If the folding rate is too slow or there are errors in the folding, it may cause the protein to exist in an insoluble form, leading to loss of protein function and even cause some diseases related to abnormal protein aggregation [2]. With knowledge of protein folding, researchers can target specific steps in the folding process to design drugs that stabilize or disrupt specific conformations to achieve the desired therapeutic effect [3]. Therefore, understanding the protein folding process is of great significance for unraveling disease mechanisms and personalized medicine [4, 5].

Protein folding is an extremely intricate process that entails the spontaneous arrangement of amino acid chains into their biologically active three-dimensional structures through a series of conformational changes. Each of these changes is influenced by the surrounding solvent context [6, 7]. This complexity presents significant challenges for experimental scientists when investigating the protein folding pathway. Researchers often employ a multi-faceted approach that combines multiple experimental techniques to obtain protein folding information from different perspectives to understand its dynamic process and the formation of intermediate states [8, 9]. The complexity of experimental techniques has driven scientists to rely on computational techniques to study protein folding pathway [10, 11]. Molecular dynamics (MD) is one of the popular tools for studying protein folding dynamics. David E. Shaw et al. developed a specialized supercomputer named Anton to study the folding process of 12 proteins through equilibrium MD simulations [12]. However, tracking the folding process at the level of thermally driven residue-level dynamics is computationally demanding and often unfeasible for long timescales [13], and the molecular mechanics force fields used in MD simulations are not sufficiently accurate. To overcome the time scale limitations of MD simulations and effectively explore the complex energy landscape of proteins, a flow-based generative modeling approach has been developed to learn and sample the conformational landscape of proteins [14]. In addition to this, various efficient and enhanced sampling methods such as Pathfinder [15], MELD [16, 17], DBFOLD [18], and P3Fold [19] have also been developed to study folding order or pathway [20, 21].

However, force field models in molecular dynamics simulations or Monte Carlo (MC) conformational sampling methods typically focus on capturing stable conformations and final structures of proteins. These force fields include physical potential terms such as hydrogen bonds and hydrophobic interactions, as well as statistical potential terms like Ramachandran, to guide proteins to accurately fold into a three-dimensional structure [22], without focusing on the topological plausibility of transition states or intermediates during the folding process [23]. Therefore, designing dedicated folding force field models specifically for predicting folding pathway and intermediates is an urgent challenge in the post-AlphaFold2 era [24].

During early evolution, there may have been many disordered polypeptides or polypeptide-like molecules [25]. These peptides may function in their disordered structure without specific folding. As biological systems become more complex, a need may arise for specific 3D structures that can more efficiently perform certain biological functions [26]. During this process, evolutionary selection on foldable sequences may have led to the development of folding ability. The appearance and evolution of foldable sequences gradually became the basis of protein folding [27]. Therefore, we can try to establish a link between the folding process of proteins and the evolution of structure. As Ernst Haeckel claimed that ontogeny recapitulates phylogeny. He argues that individuals undergo a series of morphological changes during development that reflect the stages that species has gone through in its evolutionary history [28]. When taking protein folding as an example of this biological structure formation process, we can note that there may be a correlation between the evolutionary development of protein structure and its folding process. Therefore, it may be a feasible approach to exploit protein folding kinetic information and predict the protein folding pathway by exploring the evolutionary conservation of proteins through multiple structures alignments of family proteins. In fact, the structural alphabets, proposed long ago, was constructed based on statistical analysis of large amounts of protein structure data [29, 30]. These alphabets represent the most typical or frequently occurring local conformations observed in protein structures [31, 32], which has been applied to protein dynamics analysis and protein flexibility prediction [33, 34]. Moreover, after AlphaFold2 and ESMFold made breakthroughs in the protein structure prediction, DeepMind and Meta teams released structure databases of 214 million and 617 million, respectively [35, 36]. The availability of large structure databases can undoubtedly provide valuable data for the prediction of protein folding pathway.

In this work, we developed FoldPAthreader, an in silico method for predicting protein folding pathway. This work builds on PAthreader advances by exploiting folding information from 100-million-level structure databases to design folding force field model for guiding protein folding simulations. PAthreader is a previously developed remote template recognition method that uses three-track alignment to thread PDB and AlphaFold DB libraries [37]. Based on the identified remote homologs, PAthreader initially explores the folding order of protein through artificial thresholds. Compared with PAthreader, FoldPAthreader not only identified the folding intermediates free of any arbitrary thresholds, but also predicted a series of transition states from the amino acid chain to the native state. We quantified the results using the lDDT evaluation metric [38]. The results reveal the close link between protein evolution and folding. This work demonstrates that FoldPAthreader has developed into an effective tool for quantitative computational studies of protein folding and dynamics, which can provide a complement to experimental techniques. To the best of our knowledge, this work is the first folding force field model developed specifically for protein folding pathway prediction. It comprehensively uses the state-of-the-art modeling method AlphaFold2 [35], the fastest structure search tool Foldseek [39] and the most abundant structure database AlphaFold DB [40].

## Results and discussion

### FoldPAthreader overview

**Fig. 1:**
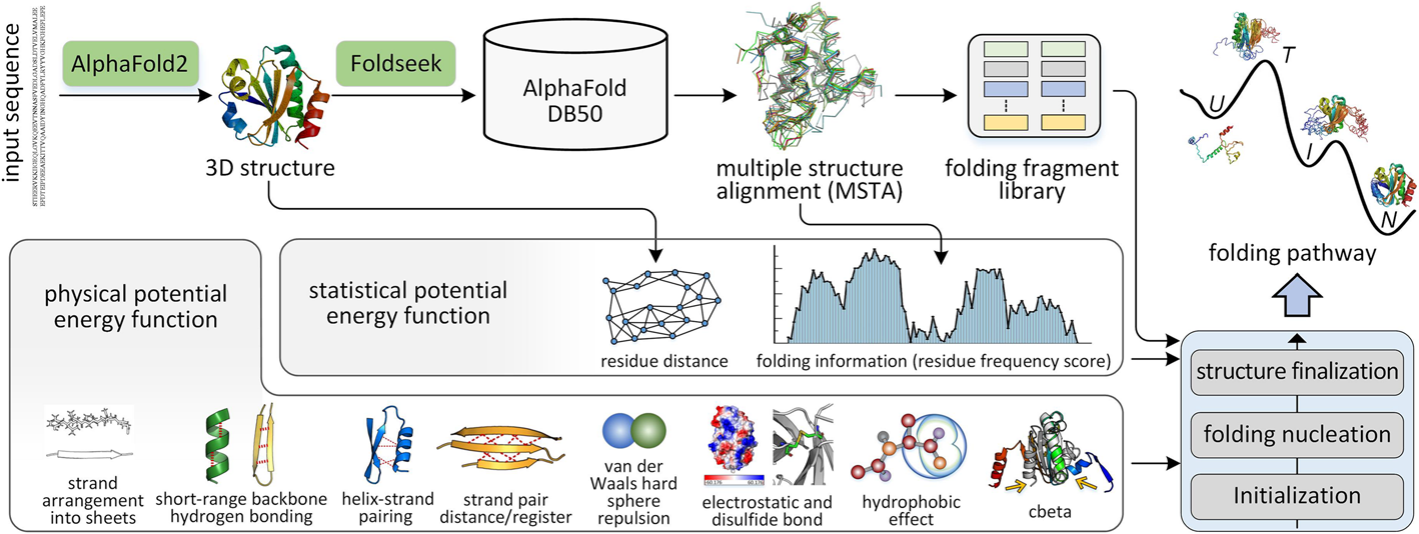
Overview of the FoldPAthreader workflow. The pipeline consists of six consecutive steps: 3D structure modeling and residue distance extraction, homologous structure search, multiple structures alignment, folding information extraction and fragment library generation, statistical and physical potential energy function construction, and folding pathway prediction. The predicted folding pathway includes unfolded state (U), transition state (T), intermediate (I), and native state (N)

The pipeline of FoldPAthreader is shown in Fig. 1, and the details are described in “Methods.” Starting from the query sequence of the target protein, the three-dimensional structure is first modeled by AlphaFold2, and remote homologs of the target are searched from the AlphaFold DB50 library through the fast structure search method Foldseek [39]. Then, structures with TM-score ≥ $\lambda$ are selected for multiple structures alignment (MSTA). $\lambda$ is 0.3, a threshold determined through experiments (Additional file 1: Table S1). Structures filtered by a lower threshold contain more noise information, while structures filtered by a higher threshold are too similar, resulting in a loss of folding information. Based on different distance deviation thresholds, the residue frequency score (*F* value) is calculated from the MSTA, where a higher value indicates a higher frequency of residue alignment at corresponding positions of structures. It reflects the conservation of protein structure during evolution. The *F* value is combined with the residual distance information extracted from the predicted structure to further design the statistical potential energy function. Meanwhile, the candidate structures screened from MSTA are traversed sequentially, and continuous fragments of at least 6 residues and at least 3 residues are added to the fragment list in a dihedral angle representation, to generate a 6-residue fragment library and a 3-residue fragment library. Additional file 2: Text S1 and Additional file 1: Table S2 describe the reasons for selecting 3- and 6-residue fragment. The fragment libraries implicitly contain folding information and are specifically used for folding pathway prediction. Finally, the protein folding pathway is predicted through three different stages of Monte Carlo conformational sampling based on fragment assembly guided by statistical and physical potential energy force fields with different energy terms and weights.

### Comparison with biological experimental data

**Fig. 2:**
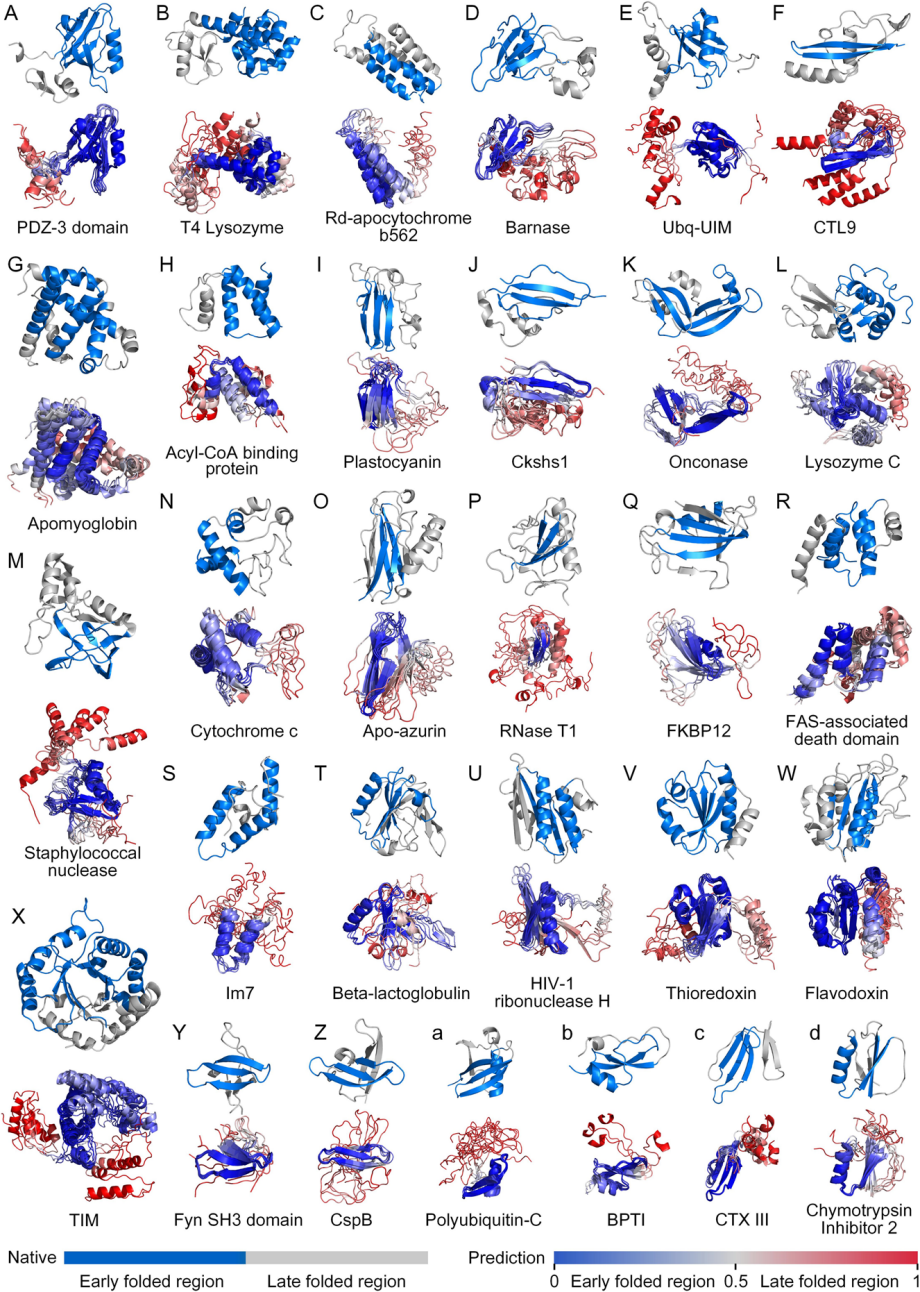
The results of 30 proteins. The blue-grey structure is an annotated folding order in the native state. The blue regions are first folded, followed by the gray. The red-white-blue structures are the intermediate ensembles predicted by FoldPAthreader, which are color-coded by the average ${\text{RMSD}}_{\text{norm}}$ (Additional file 2: Text S2) of the residues of the intermediate ensembles. The color blue indicates high overlap in the predicted intermediate ensemble, suggesting that folding occurs preferentially during the prediction process. The color red indicates a low overlap, suggesting that folding occurs later

We collected 30 proteins to test the performance of FoldPAthreader on folding pathway prediction. These proteins have been analyzed by experimental techniques such as circular dichroism [41], hydrogen deuterium exchange mass spectrometry [42], and fluorescence resonance energy transfer [3] to obtain relevant information that can describe the folding process, including intermediates and transition states. Based on the collected evidence and descriptions of the folding order of these proteins, we annotated the residue range of the early folded region of the protein. Details are listed in Additional file 1: Table S3. The residue range of some proteins may have a deviation of 1–3 residues at the boundary because some experimental methods are biased. The experimentally determined folding order is shown in Fig. 2 with different colors. The blue regions are first folded, followed by the gray. Experiments and molecular dynamics studies generally focus on detailed investigation of one protein at a time, with each study performed under different conditions or using different techniques [12]. We performed multiple analyses on this dataset that focused on elucidating basic principles of protein folding without discussing the physicochemical properties of each individual protein in detail.

**Fig. 3:**
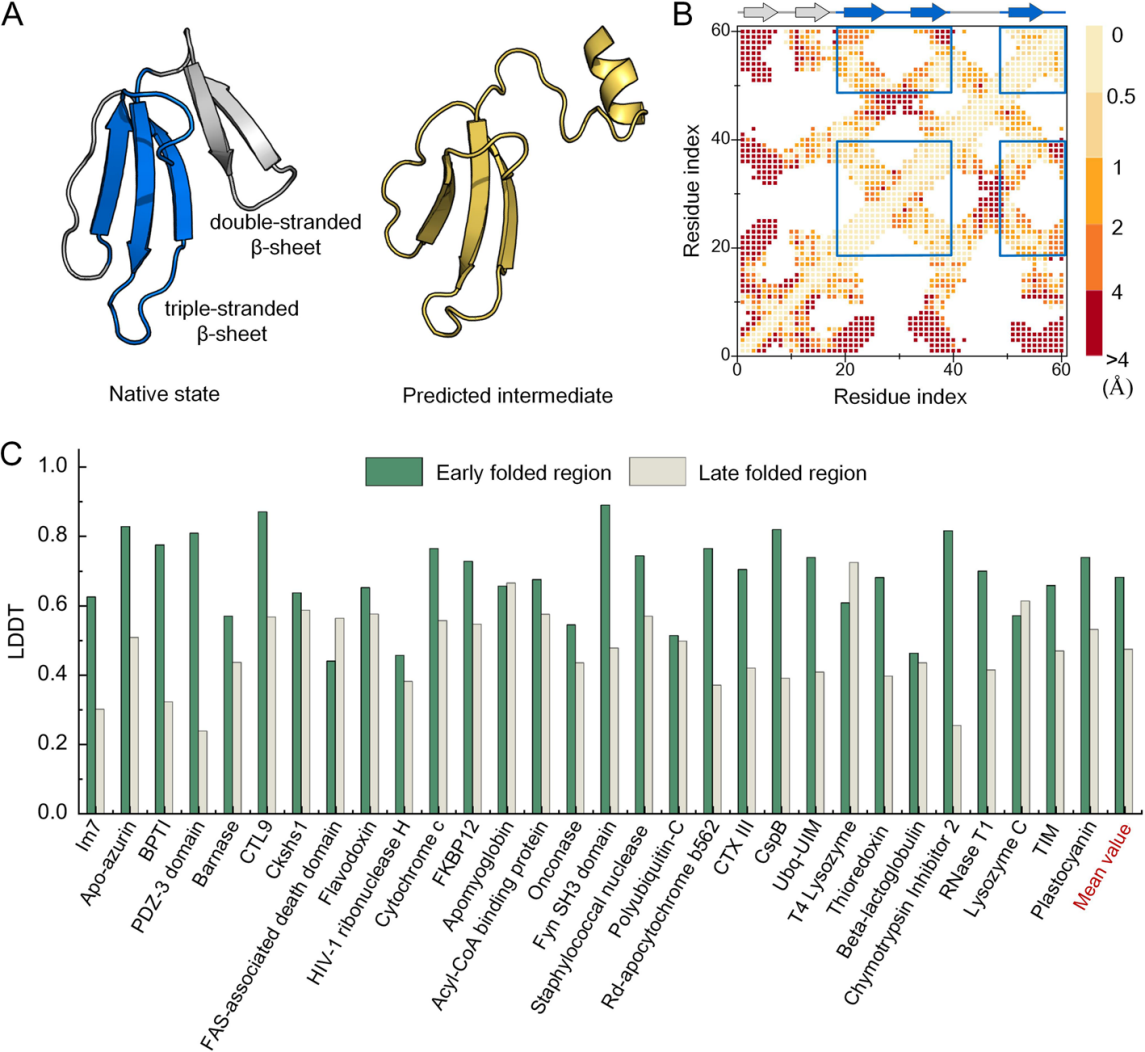
**A** The blue-gray structure is the native state of CTX III. The blue triple-stranded β-sheet are first folded, followed by the gray double-stranded β-sheet. The yellow structure is the predicted intermediate. **B** The distance difference map between the native state and predicted intermediate of CTX III (residue pairs within 15 Å). The lDDT of the EFR is calculated based on the residue pairs of the blue box and that of the LFR is the remaining map region. **C** Comparison of lDDT between EFR and LFR of 30 proteins. At the end of the histogram is the average IDDT of the 30 proteins

The folding process of FoldPAthreader is divided into three stages: initialization, folding nucleation, and structure finalization. The initialization stage uses only physical potential energy functions to guide the assembly of 3-residue fragments to initialize protein chains. The simulation of folding nucleation and structure finalization stage are performed under the guidance of statistical potential and physical potential, with different weighted energy terms and number of iterations, respectively. In the folding nucleation stage, residues with higher residue frequency score form earlier constraints with other residues. Thus, the conformations of the folding nucleation stage have a tendency that the residue pairs with earlier constraints are preferentially formed. In the structure finalization stage, the weight of the energy term of *F* value is reduced, and the overall structure is driven to fold toward the native state. Representative conformations were obtained by clustering the conformations generated from the folding nucleation stage. They are structurally superimposed as folding intermediate ensembles, and the results are shown in Fig. 2. The complete folding pathway of the 30 cases, including potential transition states, intermediates, and final states, are shown in Additional file 3: Fig. S1-30. To objectively evaluate the consistency of protein folding order between the predicted results and biological experimental data, we quantitatively measured the predicted results by IDDT score. lDDT is a scoring metric used to evaluate the local distance difference of atoms in the model, with larger values indicating greater structural similarity. It can reflect the quality of local structures at the residue level and effectively evaluate the folding order by comparing local regions of predicted intermediate and native state [38]. The IDDT of the early folded region (EFR) and late folded region (LFR) were calculated by comparing the predicted intermediates with the native structure. When the lDDT of the EFR of predicted intermediate is 10% higher than that of the LFR, it means that the early folded region forms significantly more near-native contacts than the late folded region, indicating that the folding order is consistent with biological experimental data. As shown in Fig. 3A,B, the blue triple-stranded β-sheet of CTX III is first folded [43], followed by the gray double-stranded β-sheet. The lDDT of the EFR is 0.703, which is 28.4% higher than that of the LFR (0.419), indicating that the triple-stranded β-sheet of the target is preferentially formed during the folding process.

Figure 3C presents the predicted results of 30 cases, including 4 β-sheet proteins, 6 α-helical proteins, and 20 α/β proteins. The average lDDT of EFR is 0.681 and that of LFR is 0.474. On 21 proteins, the lDDT of EFR are significantly higher than that of LFR, showing that the folding order of 70% of the proteins predicted by FoldPAthreader are consistent with the experiment data. Compared with the native state, the final state of the folding simulation has an average TM-score of 0.85, indicating that the designed folding force field model combined with the three different stage of sampling strategies are capable of folding protein to their native structure following the native folding pathway. FoldPAthreader was also compared with Pathfinder, a protein folding pathway prediction method that explores the transition probabilities of folding intermediate through conformational sampling. The results are shown in Additional file 3: Fig. S31. On 30 proteins, FoldPAthreader successfully predicted 21 proteins whose intermediates were consistent with biological experimental data, and Pathfinder successfully predicted 12 proteins. The average IDDT of early folding region and late folding region are 0.681 and 0.474 for FoldPAthreader, and 0.568 and 0.479 for Pathfinder. These results show that the performance of FoldPAthreader is significantly better than that of Pathfinder. Furthermore, MSTA-derived folding fragment libraries also contribute to accelerating the preferential formation of early folded region because the fragment libraries also contain folding information. Additional file 3: Fig. S32 shows the average RMSD of 3-residue fragments and 6-residue fragments corresponding to EFR and LFR. On most successfully predicted proteins, the fragments corresponding to LFR has a higher RMSD than EFR. These results indicate that high *F* value regions tend to be conserved and the derived fragments are similar, which facilitates the rapid assembly of this region. The fragments corresponding to low *F* value regions are diverse, making the low *F* value regions formed later in the assembly process. On the benchmark set, the predicted results are consistent with the proposed that conserved regions of protein structures are preferentially formed during folding process, proving the applicability of this principle and providing support for the method.

### The correlation between the evolution and folding

During the evolution, some proteins may undergo conservative changes in structure, that is, maintain similar structures during evolution because they perform similar functions. Other proteins may undergo innovative changes in structure, meaning they undergo structural remodeling to adapt to new environments or perform different functions [26]. If different species have proteins with similar structures or functions, it is often interpreted that these proteins may have evolved from a common ancestral protein [27]. Therefore, through the comparative analysis of protein structures across various species, it is possible to infer the evolutionary conservation of protein families, thereby enabling a deeper exploration of the folding information of individual proteins [44].

**Fig. 4:**
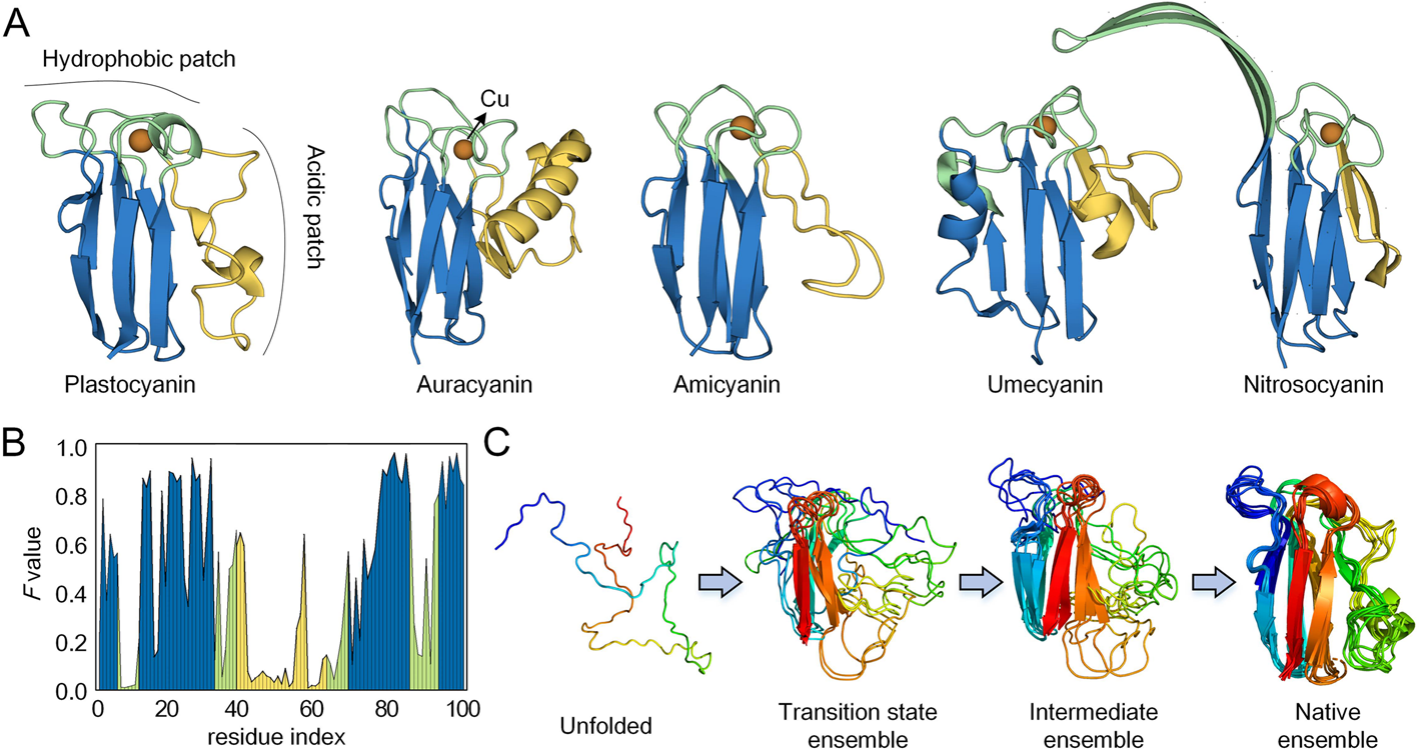
**A** 3D structure of plastocyanin (PDB ID: 9PCY) and its functionally similar auracyanin (PDB ID: 1OV8), amicyanin (PDB ID: 1ACC), umecyanin (PDB ID: 1X9R), and nitrosocyanin (PDB ID: 1IBY). **B** *F* value distribution of plastocyanin residues obtained from MSTA. **C** The folding pathway of plastocyanin simulated by FoldPAthreader from unfolded state to native state

Here, we target plastocyanin for a detailed analysis of the correlation between protein evolutionary history and folding pathway. Plastocyanin is a small copper-binding protein that receive high-energy electrons from the cytochrome *b*_6_*f* complex, and then transfer these electrons to the special reaction center P700^+^ through redox reactions [45]. From a structural point of view, as shown in Fig. 4A, plastocyanin consists of 7 β-sheets (blue β-sandwich) and random helices (green hydrophobic patch and yellow acidic patch). Amide hydrogen exchange experiments coupled with NMR spectroscopy have demonstrated the existence of a well-populated intermediate state during the folding of plastocyanin [46]. The blue β-sheet is folded first, providing the initial context for folding. The other regions (green and yellow) then gradually converge toward the β-sandwich and form the final structure [47].

From the query sequence, we predicted the folding pathway of plastocyanin protein by FoldPAthreader. In the AlphaFold DB50 structure databases, a total of 8469 structures (TM-score ≥ 0.3) were searched for global alignment with the target protein. Then, the *F* value of each residue is calculated, where a larger value indicates a higher frequency of residue alignment at the corresponding position of the target protein, as shown in Fig. 4B. It is obvious that the *F* value of the blue region is significantly higher than that of the green and yellow regions, indicating that blue β-sandwich are present repeatedly in biological structures and are highly conserved in evolution. During the folding optimization of plastocyanin, FoldPAthreader generated a total of 709 conformations, which included gradually folded transition states and intermediates as shown in Fig. 4C. The transition states and intermediate ensembles show that the β-sandwich is preferentially folded, and the other regions then gradually interact with the β-sandwich to form the final state. The results show that the folding pathway simulated by FoldPAthreader is consistent with the biological experiment [46]. This excellent performance mainly benefits from two aspects. On the one hand, the proposed folding force field focuses on the folding process, which is completely different from the traditional modeling force field such as I-TASSER and Rosetta. In the physical potential of folding force field, the hb_srbb, sheet, and rsigma terms promote the formation of secondary structures in the early stages of folding, while the hs_pair, pair, and env terms favor paired helices or sheet foldons. In the statistical potential, residues with a higher *F* value have a larger score weight, which can promote the formation of contact in the structurally conservative region earlier. By using different energy terms and weights, the three sampling stages are able to search for intermediate and transition states in the potential basin, rather than reaching the final state as quickly as possible. On the other hand, the folding fragment library can capture protein folding information from MSTA. The fragments generated from regions with high *F* values will not be diverse, resulting in conserved regions that can be rapidly formed through fragment assembly.

In MSTA, we selected four biologically significant proteins for further analysis. These proteins are members of the Copper-bind protein family or the homologous superfamily related to Copper-bind (Fig. 4A). They are auracyanin from Chloroflexus aurantiacus [47], amicyanin from Paracoccus versutus [48], umecyanin from the roots of Armoracia rusticana [49], and nitrosocyanin from Nitrosomonas europaea [50], respectively. These proteins exhibit a β-sandwich architecture akin to that of plastocyanin, with differences in the Cu-binding site region and the prominent flap on the right, which is composed of helices, random loops, or β-sheets. When the four proteins were superimposed with plastocyanin, the average TM-score was 57% for the blue region but only 38 and 34% for the green and yellow regions, respectively. The similarities and differences between these structures are determined by their respective functions and processes of evolution. It has been experimentally demonstrated that the hydrophobic patch undergoes slight conformational changes when copper is removed or mercury replaces copper in plastocyanin. These conformational differences suggest a flexible region around the copper site that allows copper to be added to the folded apoenzyme [51]. As for the acidic patch, related studies have shown that it is involved in the interaction with cytochrome and contributes to rapid electron transfer in the transient complex [52], suggesting that the hydrophobic and the acidic patch of plastocyanin are functional regions with flexibility. In the evolution of billions of years, the functional regions of proteins have undergone structural changes in order to adapt to new environmental requirements, thus deriving many homologous or remote homologous structures. For example, the nitrosocyanin monomer is part of a trimer. Its extended β-hairpins cap the copper sites of adjacent monomers, facilitating interactions through flexible conformational changes when docking with another protein [50]. For amicyanin and umecyanin, the yellow region on the right side is shorter than that of plastocyanin, and the current study has not found the functional significance of this flap. Mihwa Lee et al. concluded that it was unlikely to evolve into a smaller molecule, so it was gradually eliminated in evolution [47]. The diversity of protein structures observed within protein families is a result of evolutionary processes driven by functional selection, which reflect the evolutionary history of protein families to some extent. These pieces of evidence suggest that the correlation between protein evolutionary history and folding pathway can be revealed from the known protein universe. In addition, we analyzed the correlation between *F* values and lDDT of EFR of predicted intermediates (Additional file 3: Fig. S33A) as well as the comparison of average *F* values of EFR and LFR (Additional file 3: Fig. S33B) on 30 tested proteins. The results show that there is a certain correlation between *F* value and lDDT of EFR (Pearson *r* = 0.577), and 90% of the proteins have a higher *F* value in the EFR than in the LFR. These suggest that conserved evolutionary regions may be preferentially formed during the folding process.

### FoldPAthreader folding force field captures key features of hydrogen bonding and hydrophobic interactions

In structural bioinformatics, protein hydrogen bonding and hydrophobic interactions have always been considered the key features for determining protein folding and stability [10]. In this work, in addition to the statistical potential energy function, hydrogen bonding, and hydrophobic interactions are also included in the folding force field to capture key features of folding dynamic.

In living organisms, hydrogen bonding interactions accelerate the formation of β-sheets and α-helices during protein folding [53]. Both the β-sheet and α-helix utilize hydrogen bonding to maintain their specific secondary structures, but the arrangement of the polypeptide chains and the locations of the hydrogen bonds are distinct between the two structures. The hydrogen bonds in β-sheet are formed between the carbonyl oxygen of one strand and the amino hydrogen of an adjacent strand, which can be either parallel or antiparallel [54]. The β-sheets or β-barrels formed by the multi-strand β are very tightly bound, and their structures are stable and evolutionarily conserved, making it highly likely that β-sheets are formed preferentially during the folding process. In the α-helix, the hydrogen bonds are formed between the carbonyl oxygen atom of one residue and the amino hydrogen atom of a residue located four positions down the chain [55]. This regular pattern of hydrogen bonds stabilizes the helical structure so that individual helices may preferentially fold. But the stable interaction between helix and helix might take more time to establish. From the predicted results, it can be observed that FoldPAthreader performs differently on three secondary structure types of proteins. In order to eliminate the error caused by the prediction method, we excluded 9 poorly predicted proteins and analyzed the results of 21 proteins whose predicted folding pathways were consistent with the experimental data, as shown in Additional file 1: Table S4. The average lDDT of the EFR for β-sheet, α-helix, and α/β type protein intermediates are 0.778, 0.707, and 0.727, respectively, which are 32.4, 24.7, and 28.9% higher than the LFR respectively. It is obvious that the EFR of β-sheet folds the fastest, whereas the LFR of α-helix seems to fold faster than both the β-sheet and α/β type proteins, presenting folding characteristics that are similar to biological behavior.

To further analyze the effect of hydrogen bonding interactions in folding, we calculated the proportion of secondary structure in the conformations during the initialization stage. The initial conformations of 30 proteins contained an average of 28% helical and 3% sheet structure, which is basically consistent with the results of 12 proteins simulated by David E. Shaw et al. using Anton [12]. They reported that the initial conformation contained 16% helical and 5% sheet structure. Although the data sets are different, the FoldPAthreader results exhibit the same tendency as the MD simulations in that the proportion of helices is higher than sheets in the early folding stage, indicating that individual α-helix are formed instantaneously and much faster than individual β-sheet in the early folding stage. These results again demonstrate that FoldPAthreader is effective as well as significantly less computationally expensive than MD simulation.

In addition, early studies have emphasized the importance of distinguishing between solvent-exposed and non-solvent-exposed residues in understanding protein structure and function [56]. Here, we investigated the effect of hydrophobic interactions on protein folding nucleation by calculating the relative solvent accessibility (RSA) of residues in EFR and LFR using DSSP [57]. The RSA value of a residue is obtained by dividing the absolute accessible surface area by the residue-specific maximum accessibility value [58]. If the RSA was below 25%, the residue was classified as buried residue; otherwise, it was classified as exposed residue. The results are shown in Additional file 1: Table S5. The buried residues of EFR and LFR in the native structure are 53.2 and 39.6%, respectively, and the that of intermediate predicted by FoldPAthreader are 38.4 and 26.7%. The buried residues of the intermediates by FoldPAthreader are lower than the biological experimental data, which can be explained by the fact that the intermediates are not fully folded, resulting in more residues being exposed in solution. However, both sets of data show that EFR have higher buried residues than LFR, suggesting that hydrophobic amino acids are more prevalent in the EFR. This is consistent with experimental reports that proteins typically form a hydrophobic core region during folding [59], which reduces the free energy of the system and thus promotes further folding of the protein toward its native state. Overall, the results indicated that the folding force field of FoldPAthreader can capture key features of protein folding dynamics such as hydrogen bond and hydrophobic interactions, demonstrating FoldPAthreader’s ability to predict folding pathways.

### The folding process is conserved in homologous proteins

Some studies have reported that protein folding rates are dependent on native topology, that is, proteins with similar structures often have same folding rates even if the sequences are different [22, 60]. This suggests that the folding process may be conserved among homologs, meaning that they may have similar intermediate states or transition states during protein folding. In the datasets we collected, CspB and Fyn SH3 domain have been reported to be homologs [61, 62]. CspB is a 67-amino acid cold-shock protein from Bacillus subtilis that helps cells survive at low temperatures [63]. The Fyn SH3 domain is a protein domain consisting of 59 residues, which exists in a large number of eukaryotic proteins involved in signal transduction and cell polarization [64]. The sequence identity of the two proteins is only 22.4%, but they are similar in structure. As shown in Fig. 2Y and Z, they are composed of five β-strands arranged as two tightly packed antiparallel β-sheets, forming a closed β-barrel structure. The difference is that the triple-stranded β-sheet of CspB is composed of β1-β3, while that of Fyn SH3 domain is β2-β4. There is already sufficient evidence in the existing literature that the folding pathway between CspB and Fyn SH3 domain are similar, with folding intermediates characterized by folded triple-stranded β-sheet and unfolded remaining regions [22]. In the predicted results, the intermediate ensembles of CspB and Fyn SH3 domain are both well aligned on the triple-stranded β-sheet, which are consistent with the biological experimental data [63, 64]. Furthermore, the plastocyanidin and Apo-azurin in the datasets are also homologs and have similar experimental folding orders (Fig. 2I and O), that is, the β-sandwich is the preferred folding region [46, 65]. The predicted results showed that the lDDT of the EFR of plastocyanidin and Apo-azurin were 0.739 and 0.828 respectively, which were higher than the 0.531 and 0.508 of the LFR, indicating that β-sandwich of folding intermediate predicted by FoldPAthreader are preferentially formed. In general, the predicted results reveal the general principle that folding pathway are conserved among homologs, demonstrating that the proposed method is able to capture the potential biological properties of protein folding to some extent.

### FoldPAthreader can successfully predicted the folding pathway of BPTI and TIM

**Fig. 5:**
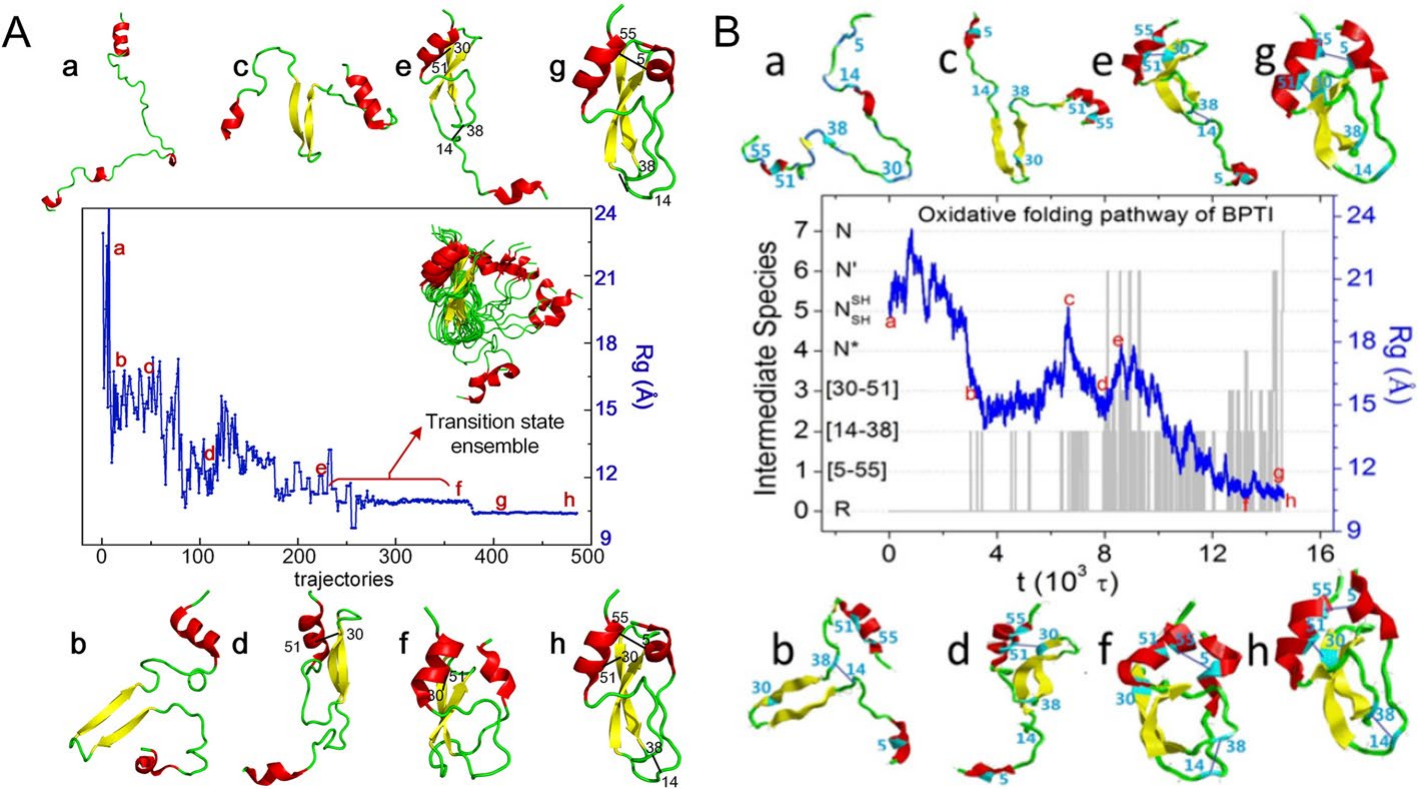
BPTI protein (PDB ID: 1QLQ). **A** The folding trajectory generated by FoldPAthreader, showing the radius of gyration from the fully reduced starting conformation to the folded state. (*a*)–(*h*) show some of the conformations sampled in the trajectory. The right side shows the transition state ensembles from conformation (*e*) to conformation (*f*). **B** 2000 oxidative folding trajectories simulated by MD. The blue curve shows the decrease in the radius of gyration (Rg). The gray lines show formation of various disulfide species labeled on the left. Snapshots (*a*)–(*h*) show some of the conformations sampled in the trajectory (The image B is from ref. [66])

**Fig. 6:**
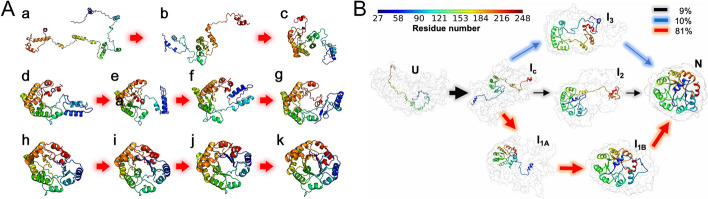
TIM protein (PDB ID: 7TIM). **A** Folding pathway predicted by FoldPAthreader. Conformation *a* is the initial state, *b,c* are the transition states, *d–g *are the intermediate states, *h*–*j* are the transition states, and *k* is the final state. **B** Multiple folding pathways simulated by MD, the upper right legend shows the transition probabilities from *I*_c_ to *I*_1A_, *I*_2_, and *I*_3_ (The image B is from ref. [67])

In addition to intermediates, we examined multiple transition states predicted by FoldPAthreader on the widely studied bovine pancreatic trypsin inhibitor (BPTI) and triosephosphate isomerase (TIM) proteins, whose folding pathways have been revealed by Meng Qin et al. and Kevin T. Halloran et al. using MD simulations [66, 67]. For comparison, we present the conformational snapshots of BPTI (Fig. 5) and TIM (Fig. 6) from FoldPAthreader and MD, respectively. Figure 5A shows the radius of gyration of BPTI, which gradually decreases from the initial conformation to the final state. Interestingly, similar to the MD, the conformations of FoldPAthreader also temporarily fall into local basin, i.e., conformation *d*, which indicates that BPTI has intermediates in the folding process. Furthermore, it can be seen from the folding trajectory of FoldPAthreader that the conformations *a*–*h* are almost consistent with the snapshots of the conformations sampled from the MD trajectory (Fig. 5B). As shown in conformations *b* and *c*, the yellow β-hairpin and C-terminal α-helix adopt a native-like structure in the early stages, and the remaining regions are disordered. Next, the C-terminal helix interacts with the β-hairpin to form a stable intermediate containing two native S–S bonds, i.e., conformations *d* and *e*. Finally, the N-terminal helix gradually converges to the C-terminus through a series of transition states to form the final state (conformations *f*–*h*). The results indicated that the folding pathway predicted by FoldPAthreader for BPTI is consistent with the maximum probability pathway of MD simulation.

Figure 6A and B present the folding pathway of TIM predicted by FoldPAthreader and simulated by MD, respectively. It can be seen that the central region of conformation *b,c* forms most of the contacts in the early folding stages. Then, the red region at the C-terminus forms contacts with the central region, i.e., conformation *d*–*g*. Finally, the blue region at the N-terminus converges toward the folded core to form the final state (conformation *h*–*k*). It is observed that the folding pathway predicted by FoldPAthreader is consistent with the third folding pathway simulated by MD (red arrow in Fig. 6B). A slight difference from the MD is the formation of the intermediates. The intermediate I_1A_ simulated by MD has a tight 7-strand barrel structure, which prevent the incorporation of N-terminus blue α and β into the barrel structure [67]. In comparison, the intermediates (conformation d-g) of FoldPAthreader exhibit a 6-strand barrel shape that includes a gap. When the N-terminus blue regions are inserted into the barrel, the overall structure becomes tighter. This suggests that there is a possibility of potential intermediates that have not been detected by MD simulation. Overall, these results again demonstrate FoldPAthreader’ ability to predict folding pathway. This protocol can greatly improve the efficiency of folding simulations compared with computationally intensive MD simulations.

### The performance of FoldPAthreader is related to the quality of MSTA

**Fig. 7:**
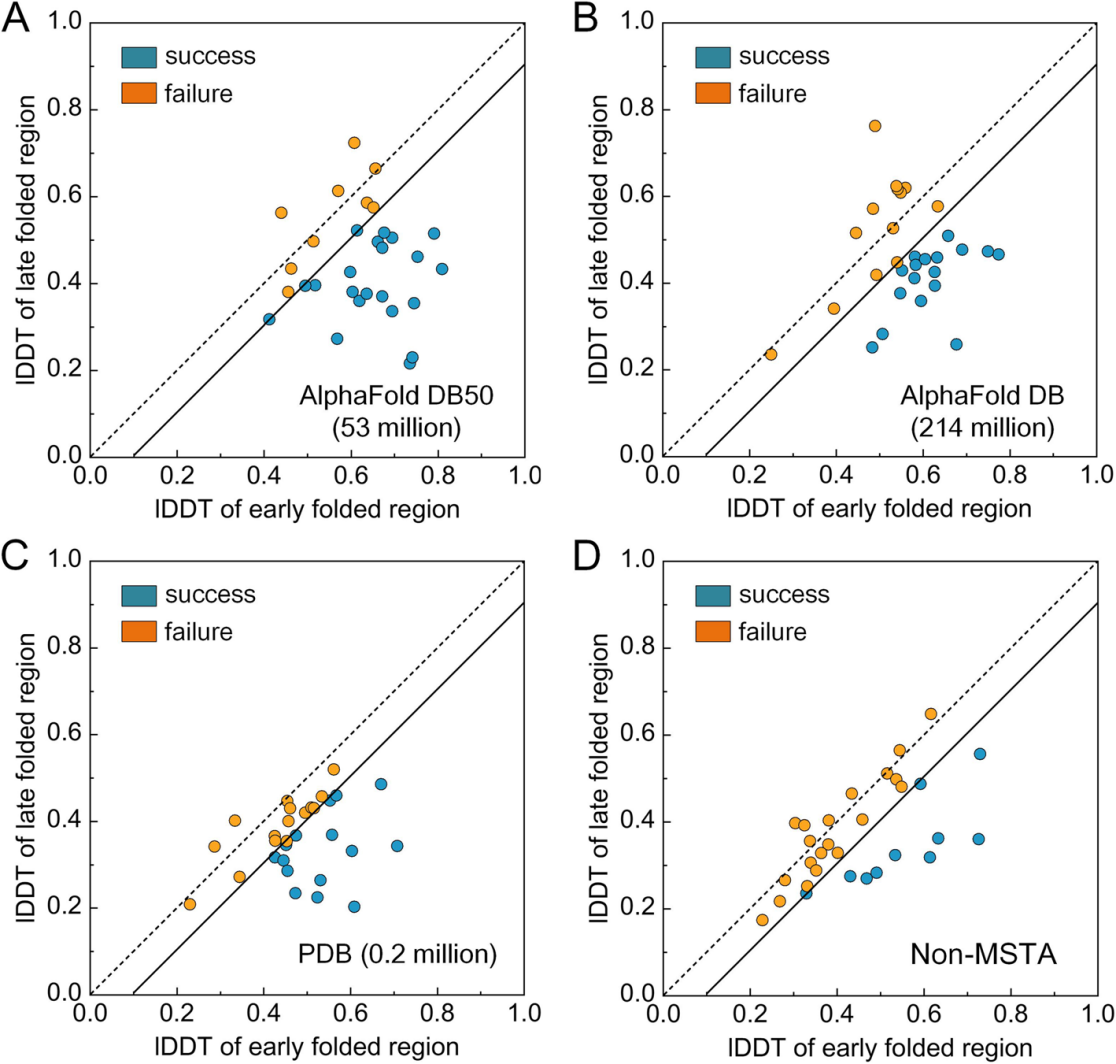
Results of MSTA ablation experiments. Head-to-head comparison between EFR and LFR of intermediates predicted by FoldPAthreader using AlphaFold DB50, AlphaFold DB, PDB, and without MSTA. The number of protein structures in the database is marked in parentheses. Blue circles are targets successfully predicted by FoldPAthreader, yellow circles are failed targets

The excellent performance of FoldPAthreader is mainly contributed by the folding force field and the folding fragment library, which are related to the quality of MSTA. Here, we examined whether and how MSTA impact the performance of FoldPAthreader by searching for MSTA from AlphaFold DB [40], AlphaFold DB50 [40], and Protein Data Bank (PDB) [68] databases respectively with the same Foldseek parameters (-s 9.5 -e 0.001 –max-seqs 10000 –alignment-type 2) [39], and without MSTA. The AlphaFold DB database, created by DeepMind and EMBL’s European Bioinformatics Institute, contains 214,683,829 entries, providing broad coverage of UniProt. AlphaFold DB50, a variant of AlphaFold DB, is a clustered database using MMseqs2 to achieve 50% sequence identity and 90% bidirectional coverage for AlphaFold DB, containing 53,665,860 structures [39, 69]. The PDB is a single global archive of three-dimensional structure data of biological macromolecules and has deposited more than 200,000 proteins as of September 2023 [68]. The results of the ablation experiments are shown in Fig. 7 and Additional file 1: Table S6. On AlphaFold DB50, the lDDT of the EFR is 0.681, which is higher than the other three performances of 0.602, 0.507, and 0.468. The number of predicted intermediates consistent with the biological experimental data also performs best on AlphaFold DB50. This is mainly due to the fact that the homologous structures from AlphaFold DB50 are more diverse than AlphaFold DB and PDB. Although AlphaFold DB has the most homologous structures, they are extremely identical and redundant, resulting in relatively little available folding information. Likewise, the smaller PDB database structure also results in limited folding information, as evidenced by the number of effective structures (Neff-str) obtained through clustering the structure of MSTA with Foldseek and counting the number of centroids. As shown in Additional file 1: Table S6, the Neff-str of AlphaFold DB50 is 562, which is double that of AlphaFold DB and PDB, indicating that the correlation between Neff-str and precision of folding pathway is significant.

Furthermore, the experimental results of Non-MSTA show the folding fragment library is essential for enabling EFR to form preferentially. Non-MSTA does not use the statistical energy function from MSTA in the folding optimization, but it uses the fragment library generated by AlphaFold DB50. As shown in Fig. 7D, the results present that there are still 9 protein folding intermediates that are consistent with the biological experimental data even without the guidance of the statistical potential function, indicating that the folding fragment library at least partially contain folding information. In general, the diversity of MSTA determines the precision of folding force field and the quality of fragments, which together drive the protein fold to its final state following the native folding pathway.

## Conclusions

At present, AI-based protein structure prediction has made a significant breakthrough. To some extent, AlphaFold2 provides only a black-box model from sequence to structure, and does not provide information about how proteins fold, which is crucial to understanding the central dogma of biology [1, 70]. In this study, we develop a protein folding pathway prediction protocol FoldPAthreader that includes a folding force field and a conformational sampling method to reveal the protein folding pathway, which is ignored by traditional protein structure prediction methods.

We developed a new folding force field model and folding fragment library with folding information by searching remote homologous structures from the known protein universe (AlphaFold DB50). Different from traditional modeling, the proposed folding force field model and fragment library not only performs high-precision modeling, but also focuses on exploring folding transition states and potential intermediates. The comparison with biological experimental data shows that the proposed folding force field at least partially captures the basic physics of protein folding. This work also proves that there is a significant correlation between the evolutionary development of protein structure and the folding process, that is, evolutionarily conserved structures are preferentially formed during the folding process. Overall, FoldPAthreader provides a new tool for revealing protein folding pathway in addition to wet-lab experiments and MD simulations. The combination of physicochemical knowledge and folding evolutionary information from homologous structures will probably emerge as a new paradigm for studying protein folding pathway in the future.

Although proposed method achieves promising results on the given dataset, we also note some challenges. First, for rare proteins, there may not be enough homologs in the structural database, which will lead to reduced performance in folding pathway prediction. Second, this method predicts protein folding pathways based on the AlphaFold models and the patterns from MSAs and is insensitive to point mutations. Third, protein folding pathways are also strongly affected by many cellular environmental factors. For example, molecular chaperones can interact with folding proteins to provide temporary structural support to prevent nonspecific interactions and aggregation. The dynamic nature of transmembrane proteins makes it very challenging to determine the structure of their folding processes. The influence of environmental factors and the dynamic interactions during protein folding may also lead to proteins containing multiple folding pathways [3, 10]. Therefore, combining biological experimental data in protein folding pathway prediction methods will be helpful to improve the prediction accuracy, which may be a potential direction for future research.

## Methods

### Data collection

Over the past few decades, numerous wet-lab experiments have been conducted to acquire a deeper understanding of protein folding and dynamics [1]. Some progress has been made in identifying the intermediates and transition states of these proteins [71, 72]. We collected biological experimental data for a total of 30 proteins, including 4 β-sheet proteins, 6 α-helical proteins, and 20 α/β proteins, with lengths ranging from 59 to 363. From the literature, we found evidence and descriptions of the folding order of 30 proteins and presented them in the Additional file 2: Text S3. We annotated the residue range of the EFR of the protein, which has an average length of 53.7% of the total length. Detailed information is listed in Additional file 1: Table S3.

### Folding information extraction

For the input sequence, the three-dimensional structure was first predicted by AlphaFold2 [35], which was used as the input structure of Foldseek [39] (parameters “-s 9.5 -e 0.001 –max-seqs 10,000 –alignment-type 2”) to search for homologous structures from AlphaFold DB50 [40]. The searched structures are globally aligned with the target protein through TM-align, and structures with TM-score < 0.3 are removed, which improves the quality of multiple structures alignment (MSTA). Then the frequency distribution *F* value of each residue of the target protein was calculated according to formula (1) and (2), which reflects the conservation of the protein structure during the evolution process.

1 $${F}_{i}= \frac{1}{N}\sum_{n=1}^{N}{\text{sco}}_{n},\text{i}\in \left[1,L\right]$$

2 $${\mathrm{sco}}_n=\left\{\begin{array}{lc}1,&\mathrm{if}\;d_i\leq2\overset\circ{\mathrm A}\\0.75,&\mathrm{if}\;2\overset\circ{\mathrm A}<d_i\leq4\overset\circ{\mathrm A}\\0.25,&\mathrm{if}\;4\overset\circ{\mathrm A}<d_i\leq5\overset\circ{\mathrm A}\\0,&\mathrm{otherwith}\end{array}\right.$$

where *L* is the length of the target protein; *N* is the homologous structures number of MSTA; ${d}_{i}$ is the Euclidean distance between the *i*th residue of the target protein and the corresponding residue of the aligned MSTA structure.

### Folding force field design

The conformational sampling process of FoldPAthreader is divided into three stages, including initialization, folding nucleation, and structure finalization. In the initialization stage, the physical potential energy function ${E}_{\text{score}1}^{\text{physi}}$ is used to guide the conformation initialization. ${E}_{\text{score}1}^{\text{physi}}$ contains two energy terms: vdw and hb_srbb. The vdw term represents only steric repulsion and avoids unreasonable conformations with atomic collisions. hbond_sr_bb is the short-range backbone-backbone hydrogen bond energy term, which is to allow the helix or adjacent β-hairpin to be quickly formed in the initial state. ${E}_{\text{score}1}^{\text{physi}}$ is defined as follows:

3 $$E_{\text{score}1}^\text{physi}=w_\text{vdw}\cdot E_\text{vdw}+w_{\text{hb}\_\text{srbb}}\cdot E_{\text{hb}\_\text{srbb}}$$

The folding nucleation stage uses physical and statistical potential energy functions. The score3 of Rosetta’s Abinitio protocol is used as a reference [73, 74], and the pair, env, sheet, hs_pair, cbeta, and rsigma terms are added to the physical potential energy function in the folding nucleation stage. ${E}_{\text{score}2}^{\text{physi}}$ is defined as follows:

4 $$\begin{array}{l}{E}_{\text{score}2}^{\text{physi}}={w}_{\text{vdw}}\cdot {E}_{\text{vdw}}+{w}_{\text{hb}\_\text{srbb}}\cdot {E}_{\text{hb}\_\text{srbb}}+{w}_{\text{pair}}\cdot {E}_{\text{pair}}\\ + {w}_{\text{env}}\cdot {E}_{\text{env}}+{w}_{\text{sheet }}\cdot {E}_{\text{sheet }}+{w}_{\text{hs}\_\text{pair}}\cdot {E}_{\text{hs}\_\text{pair}}\\ +{ w}_{\text{cbeta }}\cdot {E}_{\text{cbeta }}+{w}_{\text{rsigma}}\cdot {E}_{\text{rsigma}}\end{array}$$

where ${E}_{\text{pair}}$ is the energy term of the electrostatic and disulfide bond interaction of the residue pair; ${E}_{\text{env}}$ describes the hydrophobic effect of a particular residue; the ${E}_{\text{sheet}}$ term favors the arrangement of individual β strand into sheets. The ${E}_{\text{hs}\_\text{pair}}$ term describes the interaction between the strands and the helices. The ${E}_{\text{cbeta}}$ is another solvation term intended to correct for excluded volume effects introduced by the simulation and favor compact structures. ${E}_{\text{rsigma}}$ scores strand pairs based on the distance between them and the register of the two strands [75]. Different weights are used for each energy term, and the parameters are shown in Additional file 1: Table S7. The statistical potential energy function ${E}_{\text{score}1}^{\text{stati}}$ is designed based on the folding information extracted from MSTA, which is defined as follows:

5 $${E}_{\text{score}1}^{\text{stati}}= \sum_{i=1}^{L} \sum_{j=1}^{L}{w}_{i,j}\cdot \frac{\left|{d}_{i,j}{-\overline{d} }_{i,j}\right|}{{d}^{*}}$$

6 $${d}^{*}=\text{log}\left(\upvarepsilon +\left|i-j\right|\right),i\ne j$$

7 $${w}_{i,j}=\frac{2{F}_{i}\times {F}_{j}}{{F}_{i}+{F}_{j}}$$

where *L* is the length of the target protein; ${d}_{i,j}$ is the distance between the *i*th and *j*th residues extracted from the 3D structure of the target protein, and ${\overline{d} }_{i,j}$ is that of the folded conformation; ${d}^{*}$ is the normalized scale; and ε is an infinitely small quantity so that ${d}^{*}$ is not zero. ${w}_{i,j}$ is the weight for the distance deviation score between the *i*th and *j*th residues, which is calculated by taking the harmonic mean of ${F}_{i}$ and ${F}_{j}$. When both ${F}_{i}$ and ${F}_{j}$ are high, ${w}_{i,j}$ will be higher. It speeds up the formation of structures corresponding to high *F* value.

In the structure finalization stage, the same physical potential energy function as in the folding nucleation stage is used, but the statistical potential energy function is different. The weight of the statistical potential energy function is removed to accelerate the region with low *F* value to converge to the folded region and form the final state. ${E}_{\text{score}2}^{\text{stati}}$ is defined as follows:

8 $${E}_{\text{score}2}^{\text{stati}}= \sum_{i=1}^{L} \sum_{j=1}^{L}\frac{\left|{d}_{i,j}{-\overline{d} }_{i,j}\right|}{{d}^{*}}$$

### Folding fragment library generation

The folding simulation of FoldPAthreader is based on fragment assembly for conformational sampling. Folding fragment library is a very important component for the protocol, which are derived from structures of MSTA. All structures were first ranked according to identity (TM-score) to the target protein. Then the top *M* structures are removed, and the remaining structures are used as candidate structures for generating fragments. Finally, each structure is traversed in turn, and contiguous fragments of at least 6 residues and at least 3 residues are added to the fragment list to generate a 6-residue fragment library and a 3-residue fragment library. The backbone and side chains of each fragment are represented in torsion space. *M* is defined as follows:

9 $$M=N\times \text{min}\left\{{F}_{i} \right|i\in [1, L]\}$$

The top *M* structures have high structural overlap with the target protein, indicating that they are very identical to the target protein. The fragments generated from these highly identical structures will not carry any folding information. In contrast, the candidate structures that were screened out had locally identical or diversified regions. Candidate structure-derived fragments can avoid exploring high-energy dead ends of conserved structure regions, which accelerates the formation of conserved regions. The flexible structure regions will be assembled into more possible conformations.

### Folding optimization

FoldPAthreader uses a Monte Carlo simulated annealing search strategy for conformational sampling. In the initialization stage, the conformation is initialized by random 20*L times of 3-residue fragment assembly. The assembled trial conformation was scored by ${E}_{\text{score}1}^{\text{physi}}$, and the Metropolis criterion was used for conformational replacement.

In the folding nucleation stage, the trial conformations in the first half of the generations were generated by 6-residue fragment assembly, and in the second half of the generations using 3-residue fragment assembly. Then, the ${E}_{\text{score}2}^{\text{physi}}$ and ${E}_{\text{score}1}^{\text{stati}}$ were used to score the trial conformation and the Metropolis criterion was used to select the conformation. The flowchart of conformation update is shown in Additional file 2: Text S4. The annealing temperatures of the physical potential and the statistical potential energy function are different. They are *kT*_physi_ = 5 and *kT*_stati_ = 2 respectively. The function of the physical potential energy function is to ensure that the conformation is physically reasonable, but the continuous reduction of physical energy during folding is not necessary. On the contrary, high annealing temperature can increase the probability of conformational update.

In the structure finalization stage, the generation of trial conformations follows the same process as in the folding nucleation stage. For the conformation update process, as shown in the flowchart of Additional file 2: Text S5, ${E}_{\text{score}2}^{\text{stati}}$ was first used to score trial conformation and Metropolis criterion was used to perform conformational replacement. If it fails, ${E}_{\text{score}2}^{\text{physi}}$ is used for scoring. This greedy search strategy speeds up the convergence of protein structures.

### Supplementary Information

Additional file 1: Table S1. Prediction results at different TM-score thresholds. Table S2. Results of 3-residues fragment and 6-residues fragment ablation experiments. Table S3. Detailed information of 30 cases. Table S4. Average lDDT of early fold regions and late fold regions of 21 successfully predicted proteins. Table S5. Proportion of buried residues in early fold regions and late fold regions. Table S6. Results of MSTA ablation experiments. Table S7. Weights of energy term for Monte Carlo conformational sampling.

Additional file 2: Text S1. The reasons for selecting 3- and 6-residues fragment. Text S2. Definition of ${\mathrm{RMSD}}_{\mathrm{norm}}$. Text S3. The descriptions and evidence of experimentally determined folding intermediates. Text S4. Flowchart of conformation update strategy in the folding nucleation stage. Text S5. Flowchart of conformation update strategy in the structure finalization stage.

Additional file 3: Fig S1-30. Representative conformations of predicted folding pathways of 30 tested proteins. Fig S31. Head-to-head comparison between early folded region and late folded region of intermediates predicted by FoldPAthreader and Pathfinder. Fig S32. The average RMSD of 3-residue fragments and 6-residue fragments of 30 test proteins. Fig S33. Correlation between F value and folding order.

Additional file 4: Review history.

## Data Availability

The source codes for protein folding pathway prediction using FoldPAthreader are now available on GitHub (https://github.com/iobio-zjut/FoldPAthreader) [76] under the MIT license. It is also been deposited to Zenodo (https://zenodo.org/records/11275735) [77] with assigned 10.5281/zenodo.11275735 under the MIT license. The FoldPAthreader web server (http://zhanglab-bioinf.com/PAthreader) was made accessible by all users. The dataset for this study is publicly available via GitHub (https://github.com/iobio-zjut/FoldPAthreader/tree/main/Benchmark) [78].
