# Supplementary material for: FoldPAthreader: predicting protein folding pathway using a novel folding force field model derived from known protein universe
Source: Genome Biol. 2024 Jun 11;25:152. doi: 10.1186/s13059-024-03291-x (PMC11167914; doi:10.1186/s13059-024-03291-x)
Supplement: Supplementary file 1 — Additional file 1: Table S1. Prediction results at different TM-score thresholds. Table S2. Results of 3-residues fragment and 6-residues fragment ablation experiments. Table S3. Detailed information of 30 cases. Table S4. Average lDDT of early fold regions and late fold regions of 21 successfully predicted proteins. Table S5. Proportion of buried residues in early fold regions and late fold regions. Table S6. Results of MSTA ablation experiments. Table S7. Weights of energy term for Monte Carlo conformational sampling. [file 13059_2024_3291_MOESM1_ESM.docx]

**Supplementary Tables**

**Table S1.** Prediction results at different TM-score thresholds. Num is the number of proteins whose predicted intermediates are consistent with experimental data.

|  | Num (consistent with experimental data) | lDDT of early folded region | lDDT of late folded region |
| --- | --- | --- | --- |
| TM-score = 0.1 | 18/30 | 0.672 | 0.473 |
| TM-score = 0.2 | 20/30 | 0.680 | 0.476 |
| TM-score = 0.3 | 21/30 | 0.681 | 0.474 |
| TM-score = 0.4 | 19/30 | 0.692 | 0.499 |
| TM-score = 0.5 | 16/30 | 0.695 | 0.514 |

**Table S2.** Results of 3-residues fragment and 6-residues fragment ablation experiments. Num is the number of proteins whose predicted intermediates are consistent with experimental data.

|  | Num (consistent with experimental data) | lDDT of early folded region | lDDT of late folded region | TM-score of final state |
| --- | --- | --- | --- | --- |
| use 3 and 6-fragment | 21/30 | 0.681 | 0.474 | 0.854 |
| use only 3-fragment | 21/30 | 0.674 | 0.499 | 0.826 |
| use only 6-fragment | 19/30 | 0.670 | 0.497 | 0.868 |
| use only 9-fragment | 17/30 | 0.695 | 0.511 | 0.877 |

**Table S3.** Detailed information of 30 cases. Residue ranges of early folded region were annotated according to descriptions in references. The residue range of some proteins may have a deviation of 1-3 residues at the boundary because some reports are not comprehensive.

| **Protein name** | **PDB ID** | **length** | **Type** | **Residue range of early fold region** | **Reference (DOI)** |
| --- | --- | --- | --- | --- | --- |
| BPTI | 1QLQ | 58 | α/β | 18-58 | 10.1073/pnas.1503909112 |
| Apo-azurin | 1AIZ | 129 | α/β | 27-36, 89-98, 106-112, 121-129 | 10.1002/prot.22099 |
| Im7 | 1AYI | 86 | α | 12-45, 66-79 | 10.1038/nsb757 |
| PDZ-3 domain | 1BE9 | 115 | α/β | 10-92 | 10.1016/j.jmb.2004.11.040 |
| Barnase | 1BGS | 110 | α/β | 1-22, 53-75, 85-110 | 10.1021/bi0362267 |
| CTL9 | 1DIV | 90 | α/β | 66-90 | 10.1002/prot.22099 |
| Ckshs1 | 1DKT | 72 | α/β | 1-21, 50-72 | 10.1016/s0022-2836(02)01202-0 |
| FAS-associated death domain | 1E3Y | 104 | α | 1-32, 47-79 | 10.1007/s00249-011-0756-6 |
| Flavodoxin | 1FTG | 168 | α/β | 1-8, 47-53, 80-87, 98-110, 139-143, 151-168 | 10.1006/jmbi.1998.2045 |
| HIV-1 ribonuclease H | 1HRH | 125 | α/β | 13-21, 46-72, 89-110, 125-1, 13-21, 46-72, 89-110 | 10.1002/pro.5560071014 |
| Cytochrome c | 1I5T | 104 | α | 1-14, 61-69, 87-104 | 10.1073/pnas.1706196114 |
| FKBP12 | 1J4H | 107 | α/β | 21-30, 56-65, 71-76, 96-107 | 10.1002/prot.22099 |
| Apomyoglobin | 1MBC | 153 | α | 7-18, 24-44, 61-78, 102-142 | 10.1073/pnas.0804033105 |
| Acyl-CoA binding protein | 1NTI | 86 | α | 1-36, 65-86 | 10.1073/pnas.0509100103 |
| Onconase | 1ONC | 103 | α/β | 17-36, 55-99 | 10.1021/bi961085c |
| Fyn SH3 domain | 1SHF | 59 | β | 25-30, 36-41, 47-51 | 10.1073/pnas.0404436101 |
| Staphylococcal nuclease | 1STN | 136 | α/β | 1-36, 66-90 | 10.1016/j.bbrc.2008.08.073  10.1073/pnas.91.2.449 |
| Polyubiquitin-C | 1UBQ | 76 | α/β | 1-16, 22-34, 64-76 | 10.1073/pnas.89.6.2017  10.1021/bi00161a019 |
| Rd-apocytochrome b562 | 1YYJ | 104 | α | 22-43, 65-89 | 10.1073/pnas.0501372102 |
| CTX III | 2CRT | 60 | β | 19-39, 49-60 | 10.1074/jbc.273.17.10181 |
| CspB | 2F52 | 67 | β | 1-19, 46-53 | 10.1016/j.jmb.2004.04.011 |
| Ubq-UIM | 2KDI | 114 | α/β | 11-80 | 10.1016/j.bpc.2011.05.004 |
| T4 Lysozyme | 2LZM | 164 | α/β | 1-11, 65-164 | 10.1016/j.jmb.2006.10.048 |
| Thioredoxin | 2TRX | 108 | α/β | 1-90 | 10.1016/j.bbapap.2014.11.004 |
| Beta-lactoglobulin | 3BLG | 162 | α/β | 91-162 | 10.1038/84145 |
| Chymotrypsin Inhibitor 2 | 3CI2 | 64 | α/β | 12-34, 45-50 | [10.1016/j.polymer.2003.10.092](https://doi.org/10.1016/j.polymer.2003.10.092) |
| RNase T1 | 3RNT | 104 | α/β | 56-61, 75-81, 86-92 | 10.1002/pro.5560060702 |
| Lysozyme C | 6LYZ | 129 | α/β | 1-39, 85-129 | 10.1110/ps.8.1.35 |
| TIM | 7TIM | 247 | α/β | 1-164 | 10.1016/s0022-2836(02)01100-2 |
| Plastocyanin | 9PCY | 99 | β | 1-6, 13-15, 18-31, 69-83, 93-99 | 10.1021/bi00097a005 |

**Table S4.** Average lDDT of early fold regions and late fold regions of 21 successfully predicted proteins.

|  | | β-sheet proteins | α-helix proteins | α/β proteins |
| --- | --- | --- | --- | --- |
| lDDT | early fold region | 0.778 | 0.707 | 0.727 |
|  | late fold region | 0.454 | 0.460 | 0.438 |

**Table S5.** Proportion of buried residues in early fold regions and late fold regions. Residues with a relative solvent accessibility of less than 25% are classified as buried residues.

|  | Native of experimental | | | | Intermediate of FoldPAthreader | | |
| --- | --- | --- | --- | --- | --- | --- | --- |
|  | early fold region | late fold region | | | early fold region | | late fold region |
| Buried residue | 53.2% | | 39.6% | 38.4% | | 26.7% | |

**Table S6.** Results of MSTA ablation experiments. The number of effective structures is obtained by clustering similar structures of MSTA through Foldseek.

|  | lDDT of early folded region | lDDT of late folded region | num (consistent with experimental data) | effective structures of MSTA |
| --- | --- | --- | --- | --- |
| AlphaFold DB50 | 0.681 | 0.474 | 21/30 | 562 |
| AlphaFold DB | 0.602 | 0.485 | 17/30 | 202 |
| PDB | 0.507 | 0.373 | 15/30 | 291 |
| Non-MSTA | 0.468 | 0.382 | 10/30 | 0 |

**Table S7.** Weights of energy term for Monte Carlo conformational sampling.

| $\boldsymbol{w}_{\mathbf{vdw}}$ | 1.0 |
| --- | --- |
| $\boldsymbol{w}_{\boldsymbol{hb\_srbb}}$ | 0.5 |
| $\boldsymbol{w}_{\mathbf{pair}}$ | 1.0 |
| $\boldsymbol{w}_{\mathbf{env}}$ | 1.0 |
| $\boldsymbol{w}_{\mathbf{sheet}}$ | 1.0 |
| $\boldsymbol{w}_{\boldsymbol{hs\_pair}}$ | 1.0 |
| $\boldsymbol{w}_{\mathbf{cbeta}}$ | 0.5 |
| $\boldsymbol{w}_{\mathbf{rsigma}}$ | 1.0 |
