# Supplementary material for: FoldPAthreader: predicting protein folding pathway using a novel folding force field model derived from known protein universe
Source: Genome Biol. 2024 Jun 11;25:152. doi: 10.1186/s13059-024-03291-x (PMC11167914; doi:10.1186/s13059-024-03291-x)
Supplement: Supplementary file 2 — Additional file 2: Text S1. The reasons for selecting 3- and 6-residues fragment. Text S2. Definition of ${\mathrm{RMSD}}_{\mathrm{norm}}$RMSDnorm. Text S3. The descriptions and evidence of experimentally determined folding intermediates. Text S4. Flowchart of conformation update strategy in the folding nucleation stage. Text S5. Flowchart of conformation update strategy in the structure finalization stage. [file 13059_2024_3291_MOESM2_ESM.docx]

**Supplementary Text**

**Text S1**

We tested the performance using only 3-residue fragments, 6-residue fragments, and 9-residue fragments on 30 test proteins, and the results are shown in **Additional file 1: Table S2**. It can be seen that the performance of prediction using only 3-residues fragments is consistent with using both 3-residues and 6-residues fragments. But the TM-score of its final state is lower than that of 6-residues and 9-residues fragment. In contrast, although high-precision final state structures were obtained for 6-residues and 9-residues fragment, the number of predicted intermediates consistent with experimental data was lower than that 3-residues fragment. This is because MSTA-derived large fragments can assemble to form conformations faster than small fragments, accelerating the convergence of conformation, but they cannot explore conformations with diverse angles in small steps like 3-residues fragment. Therefore, conformational sampling through cooperative assembly of 3-residues and 6-residues fragment may be the optimal strategy in this method.

**Text S2**

$\mathrm{RMSD}_{\mathrm{norm}}$ is obtained by normalizing RMSD using a method similar to TM-score, which is used to encode the color of the predicted intermediates ensemble. The value range is from 0 to 1 and is defined as follows:

$$\mathrm{RMSD}_{\mathrm{norm}}= \frac{1}{1+\left( \frac{d_{i}}{d_{0}} \right)^{2}} (1)$$

$$d_{0}=1.24\sqrt[3]{L-15}-1.8 (2)$$

where $d_{i}$ is the distance between the *i*-th pair of residues. $d_{0}$ is a scale to normalize the match difference, which comes from the definition of TM-score. $L$ is the length of the protein.

**Text S3.** We collected descriptions and evidence of experimentally determined folding intermediates for 30 cases. They are present in (1) to (30).

1. **PDZ-3 domain**

**Description from ref.[1]:** “Figure 1. The structure of PDZ-3 domain (pdb code: 1BE9). Residue F41 is mutated to Trp to provide a fluorescence probe. The hidden intermediate has the red region unfolded.”

“Figure 8. Proposed folding pathway for PDZ-3 with different concentrations of GdmCl. The blue filled oval represents the folded region of the intermediate. The red filled oval represents the region that is unfolded in the intermediate.”

1. **Barnase**

**Description from ref.[2]:** “FIGURE 10: Free energy diagram illustration of the folding pathway of barnase under native conditions. Light gray represents unfolded regions. Colored regions represent folded regions.”

1. **BPTI**

**Description from ref.[3]:** “In several of the folding trajectories, such as the one in figure, we find that in the conversion process between two native-like disulfide bond intermediates nonnative species, [30–51, 5–38] and [30–51, 5–14], are transiently populated. These were precisely the ones identified in experiments.”

1. **Apo-azurin**

**Description from ref.[4]:** “The strongest consolidation of apo-azurin is observed in and around S3 with some diffuse contacts to S4–S6. Overall the structural consolidation is 43%.”

1. **Im7**

**Description from ref.[5]:** “In the first millisecond of Im7 folding, a highly structured intermediate forms that contains three of the four native helices (Ⅰ, Ⅱand IV). The core of this intermediate is specific in that some of the hydrophobic side chains are not buried, most notably all of those that are highly exposed to solvent in the native state.”

1. **CTL9**

**Description from ref.[4]:** “Overall this has one of the most weakly consolidated transition states with an average Φ_#_ of 0.21. However, a very clear clustering of some highly consolidated parts involving the S2-loop-S3 motif is observed.”

1. **Ckshs1**

**Description from ref.[6]:** “The results show that ckshs1 folds sequential pairs of β-strands first (β1/β2 and β3/β4). Subsequently, these pairs pack against each other and onto the α-helical region to form the core.”

1. **FAS-associated death domain**

**Description from ref.[7]:** “Thus, it appears that, while all six helices independently and cooperatively form concomitant with the hydrophobic collapse, only helices 1, 2, 4, and 5 interact in the transition state structure, with helices 3 and 6 associating on a later folding timescale.”

1. **Flavodoxin**

**Description from ref.[8]:** “We suggest that the structured part of the putative intermediate is composed of the elements of secondary structure which have the slowest exchanging amide protons in the native protein. These elements are strands β1, β3, β4 and β5 and helices α4 and α5.”

1. **HIV-1 ribonuclease H**

**Description from ref.[9]:** “Refolding of the isolated HIV RNase H domain shows a kinetic intermediate detectable by stopped-flow far UV circular dichroism and pulse-labeling H/D exchange. In this intermediate, strands 1, 4, and 5 as well as helices A and D appear to be structured.”

1. **Cytochrome c**

**Description from ref.[10]:** “Experiment shows that, under equilibrium native conditions, cytochrome c unfolds by stepping energetically uphill through a ladder of forms that differ one from the next by the unfolding of one more native-like foldon (far right). HX MS experiments during kinetic folding demonstrate a pathway that steps sequentially downhill through the same intermediates. These results are able to specify the stepwise pathway in close to 3D structural detail (rather than as a 1D projection onto some reaction coordinate) because the downhill kinetic folding units and the uphill equilibrium unfolding units are very similar to the foldons that compose the native structure.”

1. **FKBP12**

**Description from ref.[4]:** “The folding appears to have progressed already beyond initial nucleation. Strong interactions in the molecule are observed in and between many residues of S2, H, S5, S6.”

1. **Apomyoglobin**

**Description from ref.[11]:** “Fig. 4. Location of the most rapidly protected amides. The above figure shows the location of the most rapidly protected amides. Distribution of fully and nonequilibrated amides in the 0.4-ms intermediate ensemble. The structure of holomyoglobin is represented as a tube of varying radius. Residues whose amides are fully equilibrated after 0.4 ms of refolding, i.e., for which [NH_open_ has reached the equilibrium value, are depicted in magenta with increased tube radius. Residues for which [NH_open_ has not reached the equilibrium values and which are fluctuating between folded and unfolded states are depicted by a thinner blue tube. Very thin tubes represent regions that do not exhibit exchange protection and probably remain unstructured in the folding intermediate.”

1. **Acyl-CoA binding protein**

**Description from ref.[12]:** “The major folding transition state has been characterized in detail by value analyses of a large number of mutants, revealing that interactions between helices 1, 2, and 4 exist in this state.”

1. **Onconase**

**Description from ref.[13]:** “On the other hand, I_Φ_ has well-established secondary structure involving the second helix and a large part of the α-sheet region, while the other parts of the protein remain unstructured in this intermediate.”

1. **Fyn SH3 domain**

**Description from ref.[14]:** “The model’s folding intermediate is structured in the three β-strands that make up the protein’s core and is strikingly similar to intermediates detected in a recent NMR study of Fyn SH3 folding and to folding transition states elucidated in mutagenesis studies of SH3 domains. The unfolding intermediate is formed by dissociation of the folded protein’s two terminal β-strands from its core.”

“Transient intermediates on the protein's folding pathways are represented by 50 structures collected from the simulation data as described in the text. The N-terminal (strand β1, RT loop), core (strands β2, β3, β4; n-src, distal loops), and C-terminal (3_10_ helix, strand β5) portions of the protein are drawn in blue, black, and red, respectively. Each of the structures in the ensembles is aligned with respect to strand β3.”

1. **Staphylococcal nuclease**

**Description from ref.[15]:** “According to the previous NMR study, the N-terminal β1-β3 are the most stable structure formed at the first step of the folding process. Then, after the remaining C-terminal β4-β5 and α-helices are formed, the docking of the α-domain with the β-domain follows.”

1. **Polyubiquitin-C**

**Description from ref.[16]:** “Most of the amide protons with a *P* value larger than 10^4^ are in the hydrophobic core formed by three strands of β-sheet and the α-helix, while those with *P* values less than 10^2^ are located in regions of irregular structure, or on the surface of the protein.”

**Description from ref.[17]:** “Thus, the core of ubiquitin, composed of the a-helix and β-sheet and the interface between them, is formed in a major cooperative folding event. The sheet and helix protons are protected at nearly identical rates, indicating cooperativity in the formation both of the individual elements of secondary structure and of the interfacial region. The rapid protection rates for the amide protons of Ile-23 and Leu-56 provide direct evidence for early association of the sheet and the helix.”

1. **Rd-apocytochrome *b*_562_**

**Description from ref.[18]:** “In the case of Rd-apocyt *b*_562_, a redesigned stable variant of apocytochrome *b*_562_ with a four-helix bundle fold, NHX experiments identified two partially unfolded forms (PUFs). PUF1 has the N-terminal helix and a part of the C-terminal helix unfolded. PUF2 has only the N-terminal helix unfolded.”

1. **CTX III**

**Description from ref.[19]:** “Folding kinetics of CTX III based on the amide-protection data reveals that the triple-stranded, antiparallel β-sheet segment, which is located in the central core of the molecule, appears to fold faster than the double-stranded β-sheet segment.”

1. **cspB**

**Description from ref.[20]:** “Thirdly, it highlights the residues in strand β4 (Phe49 and Ile51) and β5 (Ala60 and Val63) that are essential for the stability of the native protein (very high DDGNU values), but not of the transition state (low Φ-values). This suggests that the second β sheet is not stabilized in the transition state.”

“In the first β sheet (β1–β3) the Φ-values increase to 1 within the first half of strand β1, stay at 1 and then decrease to zero within the second half of strand β3. In the second sheet (β4–β5) the Φ-values are zero, and in the long connecting loop there is a narrow peak of intermediate to high Φ-values around residue 41. This led to the simple interpretation that in the transition state the first sheet is folded, but the second is unfolded.”

1. **Ubq-UIM**

**Description from ref.[21]:** “We have previously engineered a Ubq-UIM fusion protein that allows independent experimental manipulations of the individual domains. It takes advantage of the fact that the ubiquitin interacting motif (UIM), consisting of a 20-residue helix, binds to ubiquitin (Ubq) in an orientation that positions the C-terminus of Ubq in close proximity to the N-terminus of the UIM.”

“Thermodynamic studies of cooperativity upon temperature-induced unfolding of Ubq-UIM showed that it does not follow two-state unfolding but includes detectable population of two equilibrium intermediate states consisting of either one of the two domains (Ubq or UIM) folded and the other unfolded.”

1. **T4 Lysozyme**

**Description from ref.[22]:** “A native-state hydrogen exchange experiment subsequently detected an equilibrium intermediate that only involves the formation of the C-terminal domain.”

1. **Thioredoxin**

**Description from ref.[23]:** “The experiments show that C-terminal α-helix is mainly unfolded in transition state ensembles TSE1 and the intermediate and becomes structured in TSE2. Structure-based molecular dynamics are in agreement with these experiments and provide protein-wide structural information on transient states. In our model, thioredoxin folding starts with structure formation in the β-sheet, while the protein helices coalesce later.”

1. **Beta-lactoglobulin**

**Description from ref.[24]:** “The intermediate contains hydrogen bonded structure as measured by burst-phase labeling in the core of the β-sheet (βF, βG and βH) and the major α-helix, as well as some fluctuating helical structure near the N-terminus that is later converted into β-sheet.”

1. **Chymotrypsin Inhibitor 2**

**Description from ref.[25]:** “Our results indicate a preferred pathway for the unfolding of Chymotrypsin Inhibitor 2: it starts with the loss of native contacts between N terminus and β3 and continues with β2 and β3. Some helical contacts and some non-native contacts around the termini persist even in highly unfolded conformations of the structure.”

1. **RNase T1**

**Description from ref.[26]:** “All of the slowest exchanging amide residues are located in strands 2-4 of the central β-sheet and these residues are protected first in the early stages of folding. The residues that have somewhat lower rate constants for protection in early folding are predominantly found in the α-helix and the first strand of the small β-sheet.”

1. **Lysozyme C**

**Description from ref.[27]:** “This species contains stable hydrogen bonded structure in the helical α-domain, but lacks stable structure in the second, predominantly β-sheet, domain. Formation of the α-domain intermediate occurs with biphasic kinetics.”

**Description from ref.[28]:** “The α-helical domain (residues 1-37 and 85-123) is shown in blue and the β-sheet domain (residues 38-84) in red.”

“The structure of the molten globule is highly heterogeneous, having the highly structured α-helical domain formed by loose hydrophobic interactions whereas the β-sheet domain is significantly more unfolded.”

1. **TIM**

**Description from ref.[29]:** “To test the stability of the I_1A_ state, we further sampled another set of 100 trajectories beginning from the I_1A_ state for 8,000 time units. Even with a quadrupled simulation time, only 17% of the trajectories reached the native state, confirming the extremely long lifetime of the I_1A_ state.”

1. **Plastocyanin**

**Description from ref.[30]:** “The amide protons protected in the intermediate are shown as white spheres attached to their amide nitrogen (dark spheres) with residue numbers.”

**Text S4.** Flowchart of conformation update strategy in the folding nucleation stage.


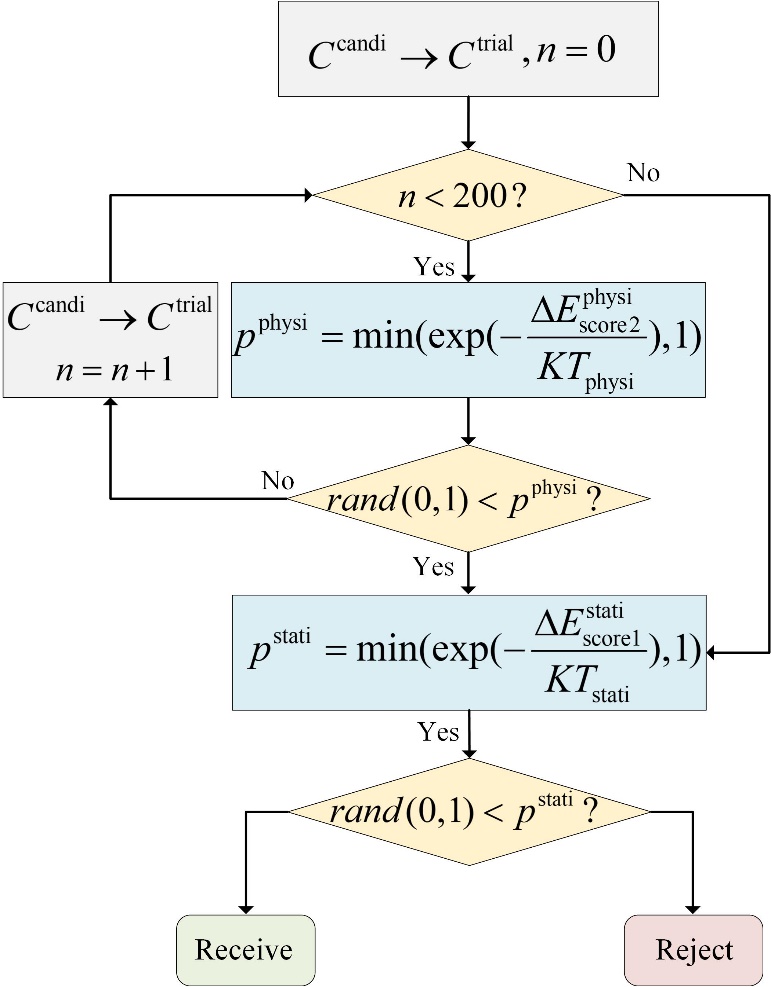


Where $\Delta E_{score2}^{\mathrm{physi}}$ is the $E_{score2}^{\mathrm{physi}}$ score difference between the trial conformation and the candidate conformation; $\Delta E_{score1}^{\mathrm{stati}}$ is the $E_{score1}^{\mathrm{stati}}$ score difference between the trial conformation and the candidate conformation; rand(0, 1) is a random number between 0 and 1; and *kT*_physi_ and *kT*_stati_ are the temperature scaling factor. Trial conformation is generated by fragment assembly of candidate conformations, which is received according $p^{\mathrm{sta}\mathrm{ti}}$ based on physical potential energy scores. If the reception fails, the fragment assembly is reperformed up to 200 times. Finally, the conformation is received based on the statistical potential energy scores.

**Text S5.** Flowchart of conformation update strategy in the structure finalization stage.


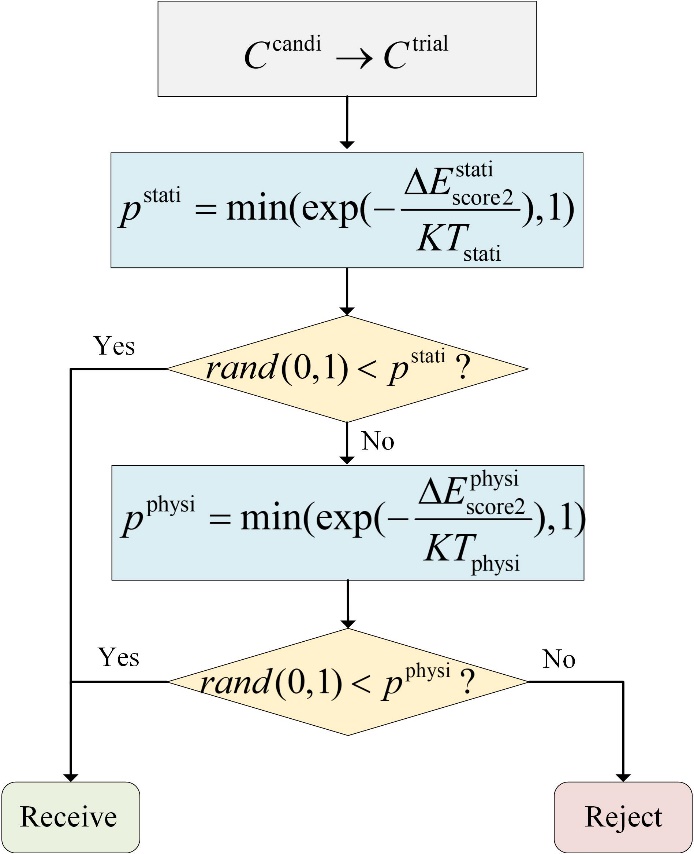


Where $\Delta E_{score2}^{\mathrm{physi}}$ is the $E_{score2}^{\mathrm{physi}}$ score difference between the trial conformation and the candidate conformation; $\Delta E_{score2}^{\mathrm{stati}}$ is the $E_{score2}^{\mathrm{stati}}$ score difference between the trial conformation and the candidate conformation; rand(0, 1) is a random number between 0 and 1; and *kT*_physi_ and *kT*_stati_ are the temperature scaling factor.

**SI References**

1. Feng H, Vu ND, & Bai Y, Detection of a hidden folding intermediate of the third domain of PDZ. *Journal of molecular biology* 346(1):345-353 (2005).

2. Vu ND, Feng H, & Bai Y, The folding pathway of barnase: the rate-limiting transition state and a hidden intermediate under native conditions. *Biochemistry* 43(12):3346-3356 (2004).

3. Qin M, Wang W, & Thirumalai D, Protein folding guides disulfide bond formation. *Proceedings of the National Academy of Sciences of the United States of America* 112(36):11241-11246 (2015).

4. Nölting B & Agard DA, How general is the nucleation-condensation mechanism? *Proteins* 73(3):754-764 (2008).

5. Capaldi AP, Kleanthous C, & Radford SE, Im7 folding mechanism: misfolding on a path to the native state. *Nature structural biology* 9(3):209-216 (2002).

6. Seeliger MA, Breward SE, & Itzhaki LS, Weak cooperativity in the core causes a switch in folding mechanism between two proteins of the cks family. *Journal of molecular biology* 325(1):189-199 (2003).

7. Greene LH, Li H, Zhong J, Zhao G, & Wilson K, Folding of an all-helical Greek-key protein monitored by quenched-flow hydrogen-deuterium exchange and NMR spectroscopy. *European biophysics journal : EBJ* 41(1):41-51 (2012).

8. Steensma E & van Mierlo CP, Structural characterisation of apoflavodoxin shows that the location of the stable nucleus differs among proteins with a flavodoxin-like topology. *Journal of molecular biology* 282(3):653-666 (1998).

9. Kern G, Handel T, & Marqusee S, Characterization of a folding intermediate from HIV-1 ribonuclease H. *Protein science : a publication of the Protein Society* 7(10):2164-2174 (1998).

10. Englander SW & Mayne L, The case for defined protein folding pathways. *Proceedings of the National Academy of Sciences of the United States of America* 114(31):8253-8258 (2017).

11. Uzawa T, et al., Hierarchical folding mechanism of apomyoglobin revealed by ultra-fast H/D exchange coupled with 2D NMR. *Proceedings of the National Academy of Sciences of the United States of America* 105(37):13859-13864 (2008).

12. Teilum K, Poulsen FM, & Akke M, The inverted chevron plot measured by NMR relaxation reveals a native-like unfolding intermediate in acyl-CoA binding protein. *Proceedings of the National Academy of Sciences of the United States of America* 103(18):6877-6882 (2006).

13. Houry WA & Scheraga HA, Structure of a hydrophobically collapsed intermediate on the conformational folding pathway of ribonuclease A probed by hydrogen-deuterium exchange. *Biochemistry* 35(36):11734-11746 (1996).

14. Ollerenshaw JE, Kaya H, Chan HS, & Kay LE, Sparsely populated folding intermediates of the Fyn SH3 domain: matching native-centric essential dynamics and experiment. *Proceedings of the National Academy of Sciences of the United States of America* 101(41):14748-14753 (2004).

15. Ishii T, et al., Probing force-induced unfolding intermediates of a single staphylococcal nuclease molecule and the effect of ligand binding. *Biochemical and biophysical research communications* 375(4):586-591 (2008).

16. Pan Y & Briggs MS, Hydrogen exchange in native and alcohol forms of ubiquitin. *Biochemistry* 31(46):11405-11412 (1992).

17. Briggs MS & Roder H, Early hydrogen-bonding events in the folding reaction of ubiquitin. *Proceedings of the National Academy of Sciences of the United States of America* 89(6):2017-2021 (1992).

18. Feng H, Zhou Z, & Bai Y, A protein folding pathway with multiple folding intermediates at atomic resolution. *Proceedings of the National Academy of Sciences of the United States of America* 102(14):5026-5031 (2005).

19. Sivaraman T, Kumar TK, Chang DK, Lin WY, & Yu C, Events in the kinetic folding pathway of a small, all beta-sheet protein. *The Journal of biological chemistry* 273(17):10181-10189 (1998).

20. Garcia-Mira MM, Boehringer D, & Schmid FX, The folding transition state of the cold shock protein is strongly polarized. *Journal of molecular biology* 339(3):555-569 (2004).

21. Patel MM, Tzul F, & Makhatadze GI, Equilibrium and kinetic studies of protein cooperativity using urea-induced folding/unfolding of a Ubq-UIM fusion protein. *Biophysical chemistry* 159(1):58-65 (2011).

22. Kato H, Vu ND, Feng H, Zhou Z, & Bai Y, The folding pathway of T4 lysozyme: an on-pathway hidden folding intermediate. *Journal of molecular biology* 365(3):881-891 (2007).

23. Vazquez DS, Sánchez IE, Garrote A, Sica MP, & Santos J, The E. coli thioredoxin folding mechanism: the key role of the C-terminal helix. *Biochimica et biophysica acta* 1854(2):127-137 (2015).

24. Kuwata K, et al., Structural and kinetic characterization of early folding events in beta-lactoglobulin. *Nature structural biology* 8(2):151-155 (2001).

25. Ozkan SB, Dalgýn GS, & Haliloglu T, Unfolding events of Chymotrypsin Inhibitor 2 (CI2) revealed by Monte Carlo (MC) simulations and their consistency from structure-based analysis of conformations. *Polymer* 45(2):581-595 (2004).

26. Mullins LS, Pace CN, & Raushel FM, Conformational stability of ribonuclease T1 determined by hydrogen-deuterium exchange. *Protein science : a publication of the Protein Society* 6(7):1387-1395 (1997).

27. Kulkarni SK, et al., A near-native state on the slow refolding pathway of hen lysozyme. *Protein science : a publication of the Protein Society* 8(1):35-44 (1999).

28. Kuwajima K, The molten globule state of alpha-lactalbumin. *FASEB journal : official publication of the Federation of American Societies for Experimental Biology* 10(1):102-109 (1996).

29. Halloran KT, et al., Frustration and folding of a TIM barrel protein. *Proceedings of the National Academy of Sciences of the United States of America* 116(33):16378-16383 (2019).

30. Koide S, Dyson HJ, & Wright PE, Characterization of a folding intermediate of apoplastocyanin trapped by proline isomerization. *Biochemistry* 32(46):12299-12310 (1993).
