# Supplementary material for: FoldPAthreader: predicting protein folding pathway using a novel folding force field model derived from known protein universe
Source: Genome Biol. 2024 Jun 11;25:152. doi: 10.1186/s13059-024-03291-x (PMC11167914; doi:10.1186/s13059-024-03291-x)
Supplement: Supplementary file 3 — Additional file 3: Fig S1-30. Representative conformations of predicted folding pathways of 30 tested proteins. Fig S31. Head-to-head comparison between early folded region and late folded region of intermediates predicted by FoldPAthreader and Pathfinder. Fig S32. The average RMSD of 3-residue fragments and 6-residue fragments of 30 test proteins. Fig S33. Correlation between F value and folding order. [file 13059_2024_3291_MOESM3_ESM.docx]

**Supplementary Figures**

Figure S1-30 shows the representative conformational ensemble of the complete folding pathway from the unfolded state to the folded state of 30 test proteins, including potential transition states, intermediates, and final states.


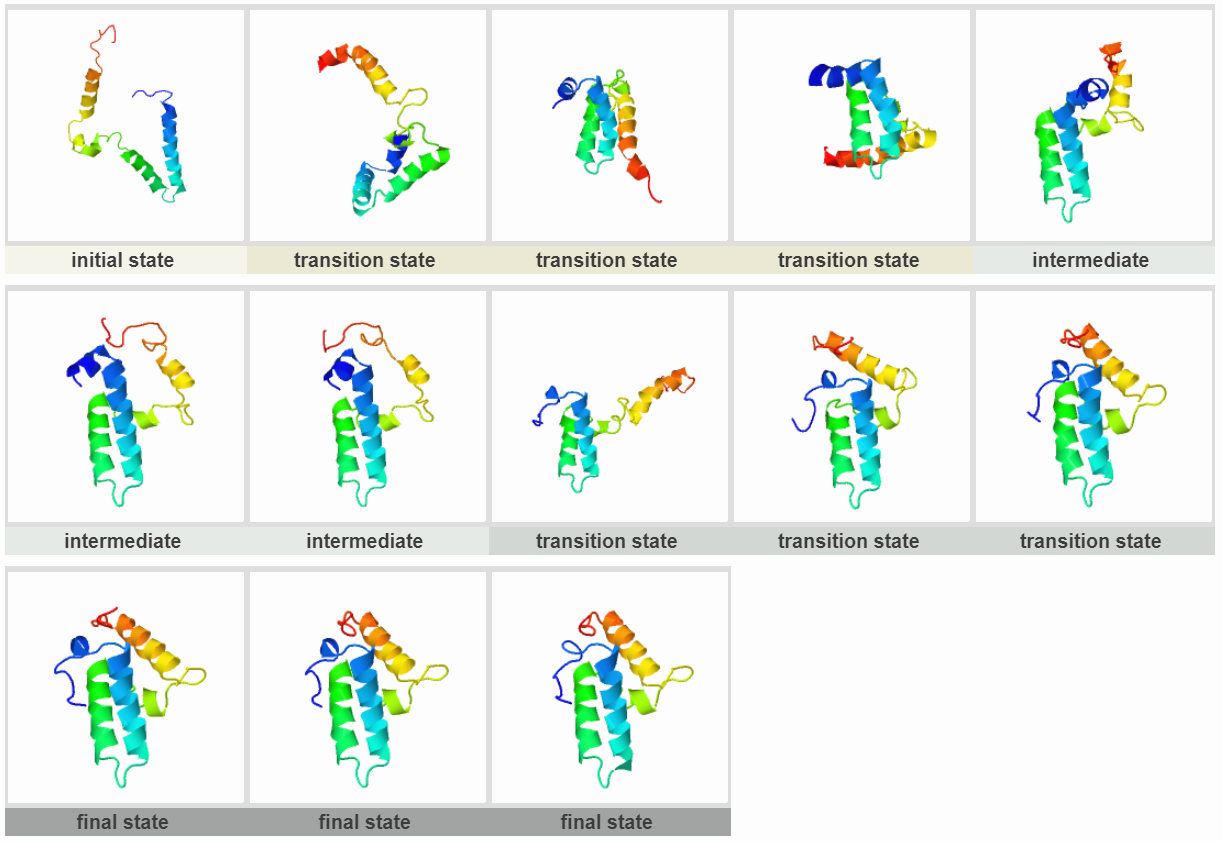


**Fig S1.** Im7 (PDB ID:1AYI)


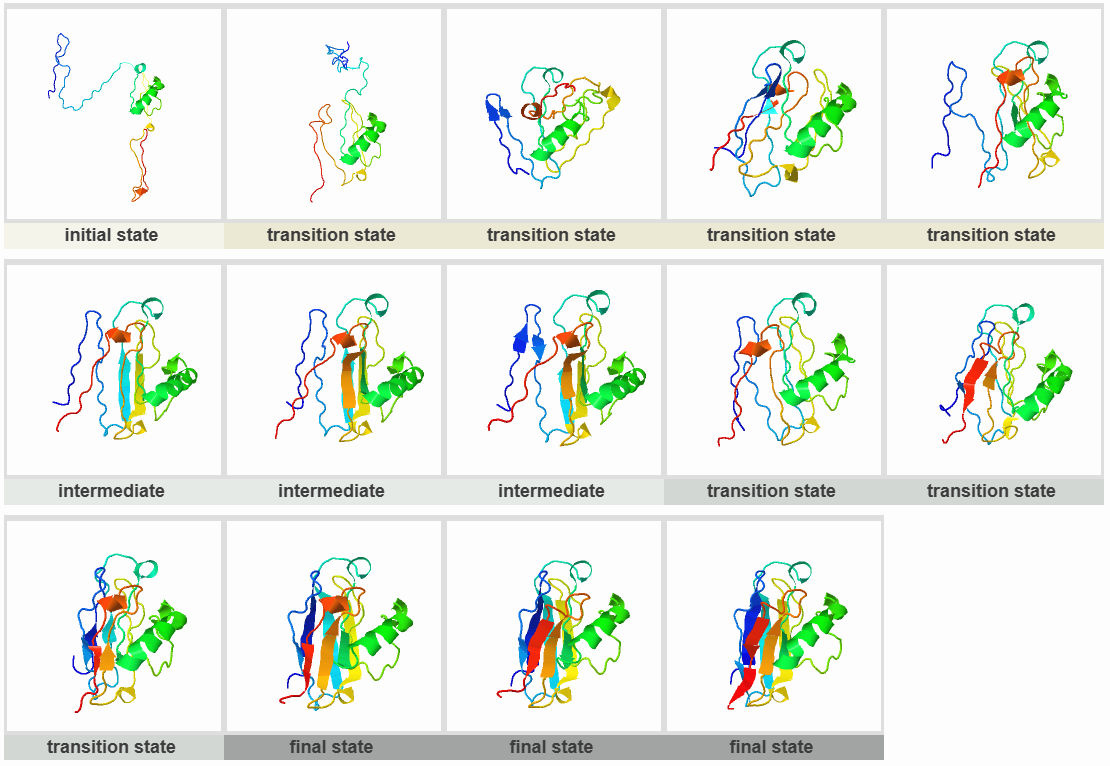


**Fig S2.** Apo-azurin (PDB ID:1AIZ)


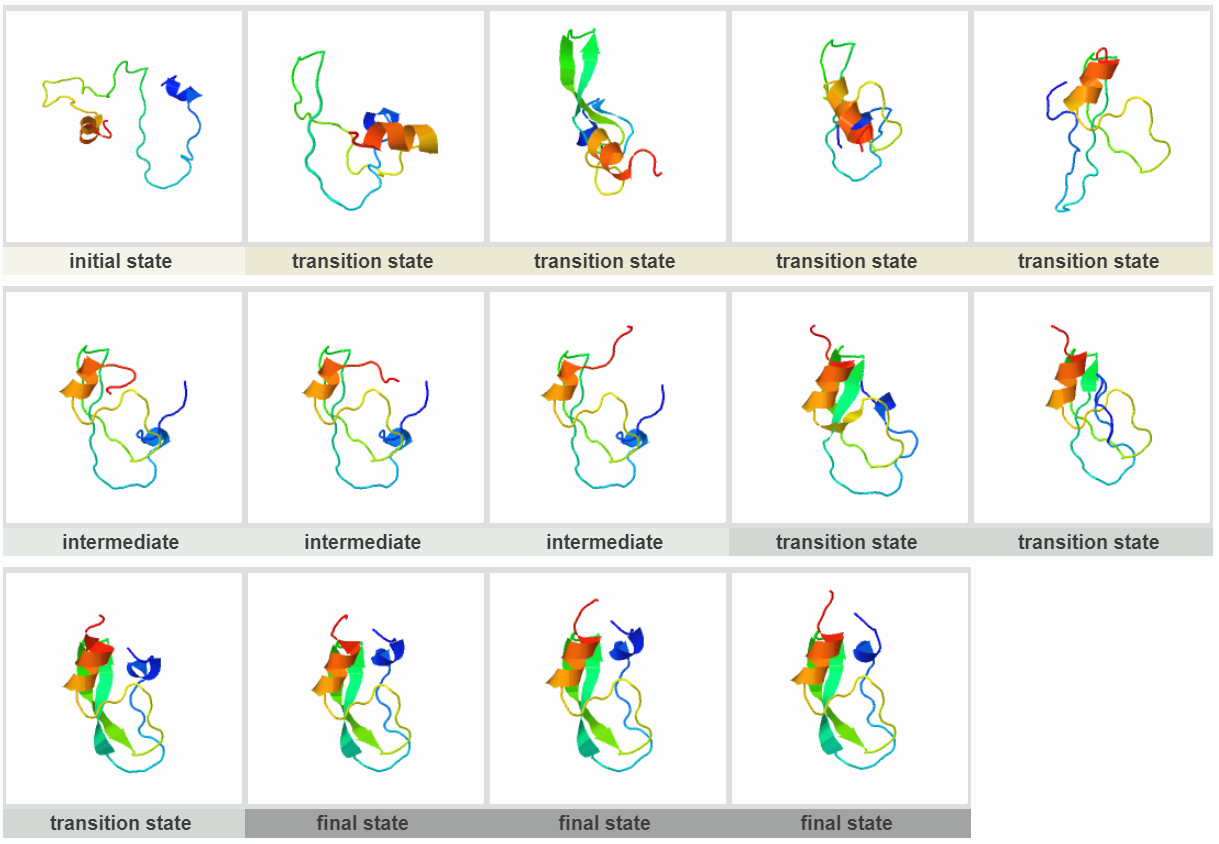


**Fig S3.** BPTI (PDB ID:1QLQ)


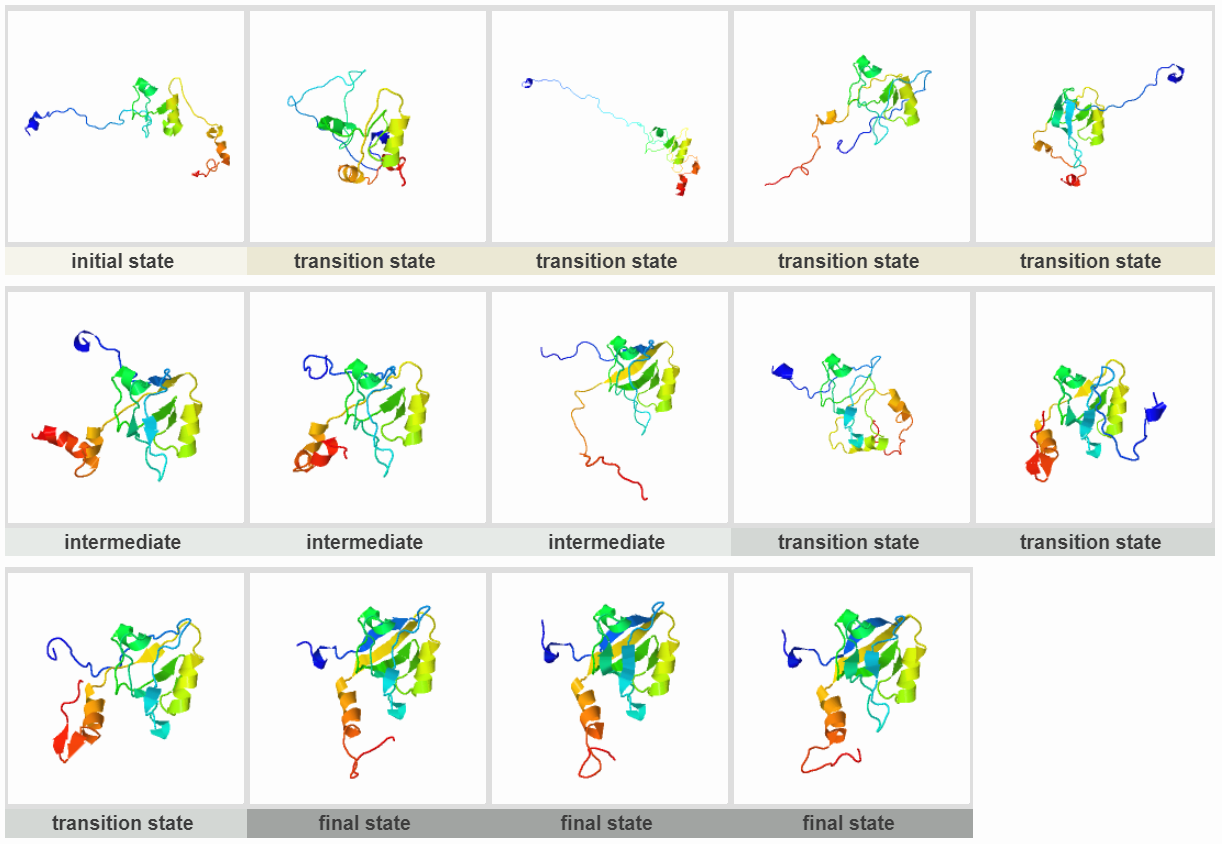


**Fig S4.** PDZ-3 domain (PDB ID:1BE9)


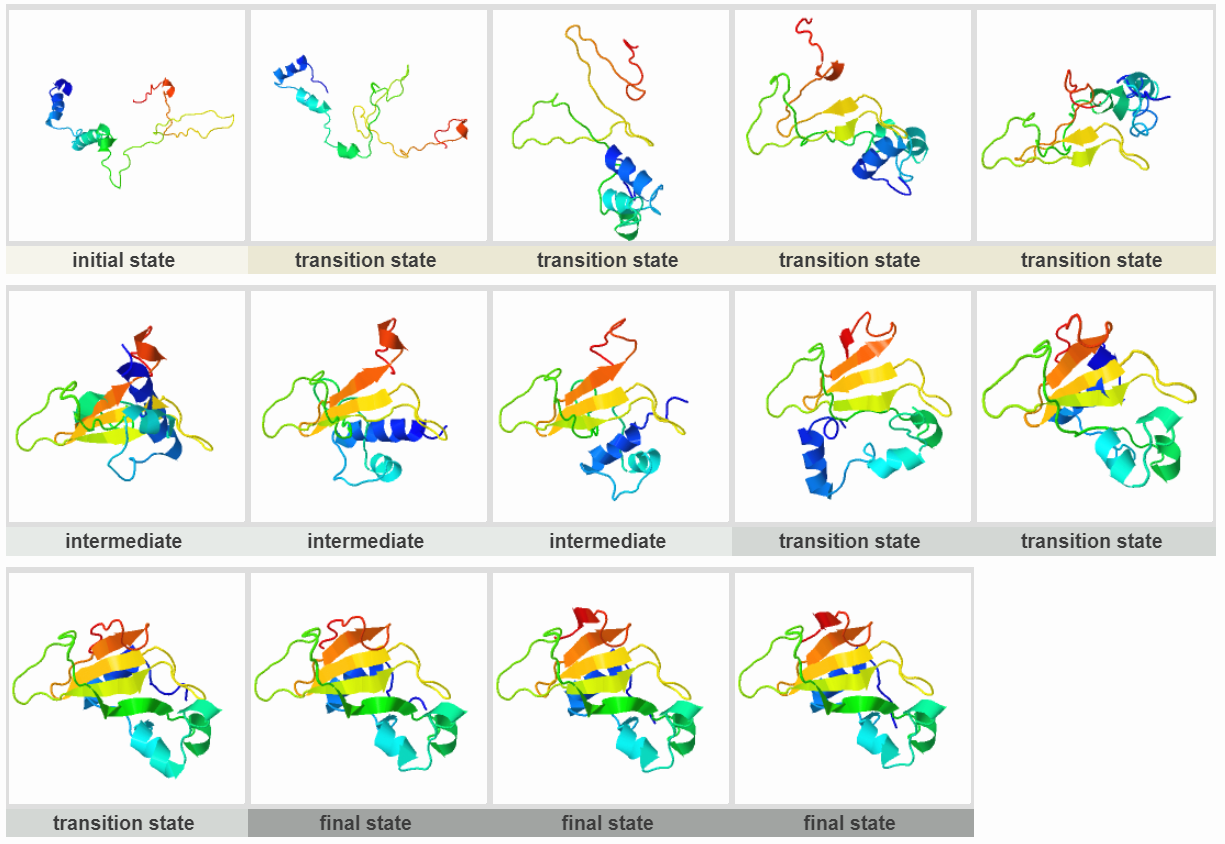


**Fig S5.** Barnase (PDB ID:1BGS)


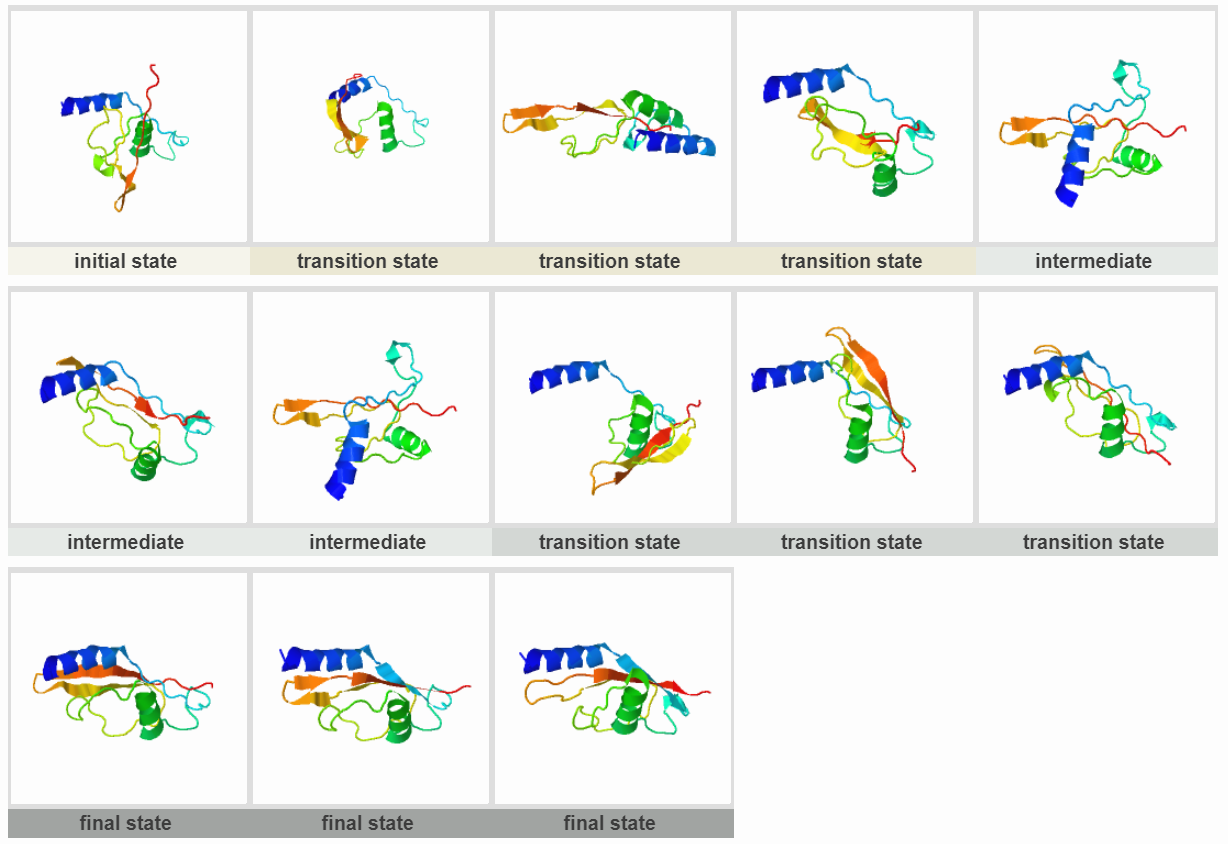


**Fig S6.** CTL9 (PDB ID:1DIV)


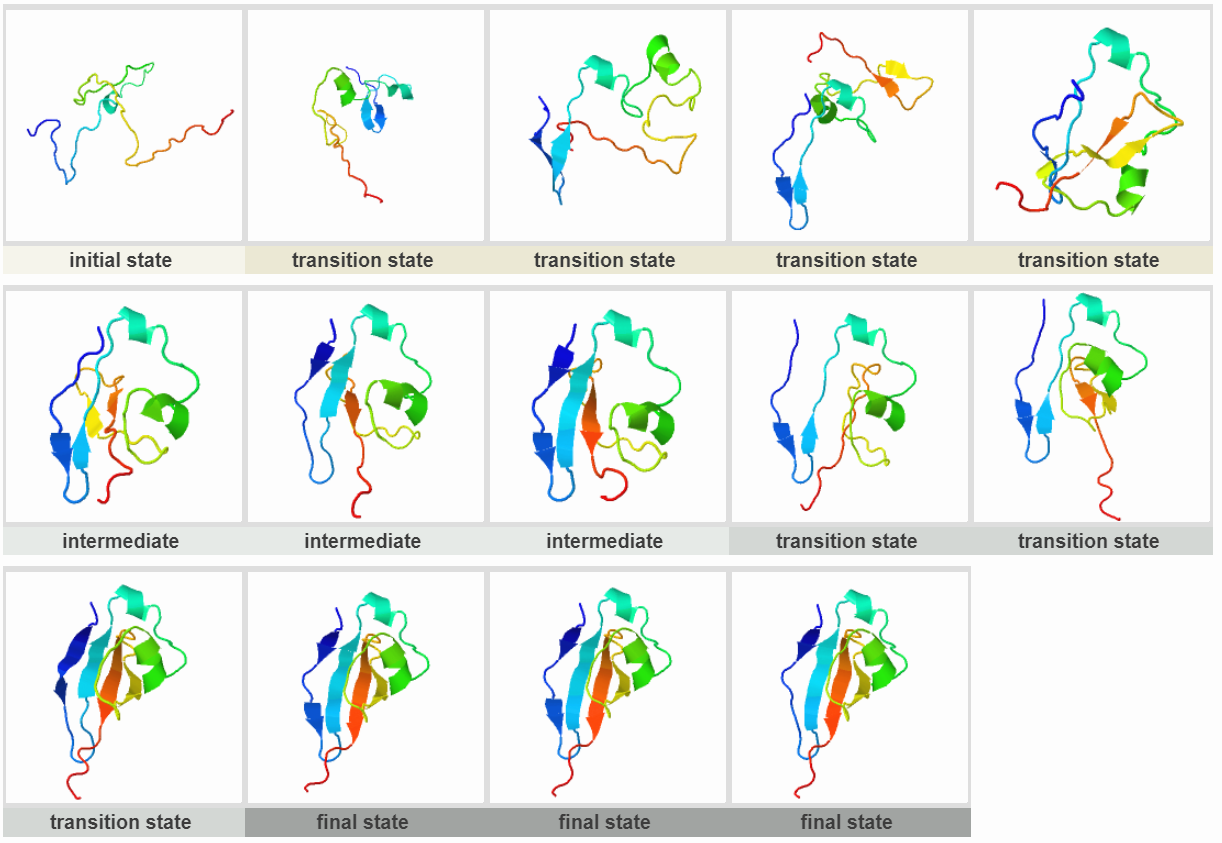


**Fig S7.** Ckshs1 (PDB ID:1DKT)


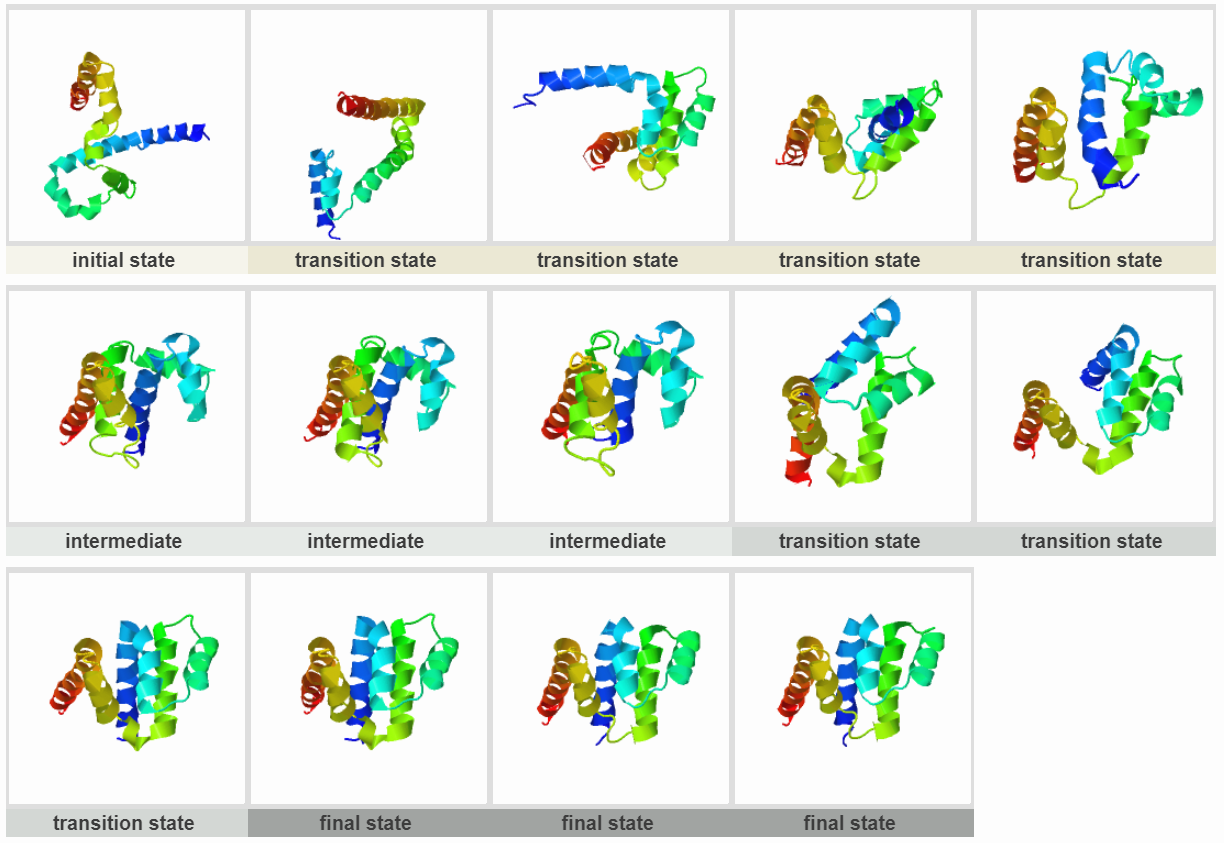


**Fig S8.** FAS-associated death domain (PDB ID:1E3Y)


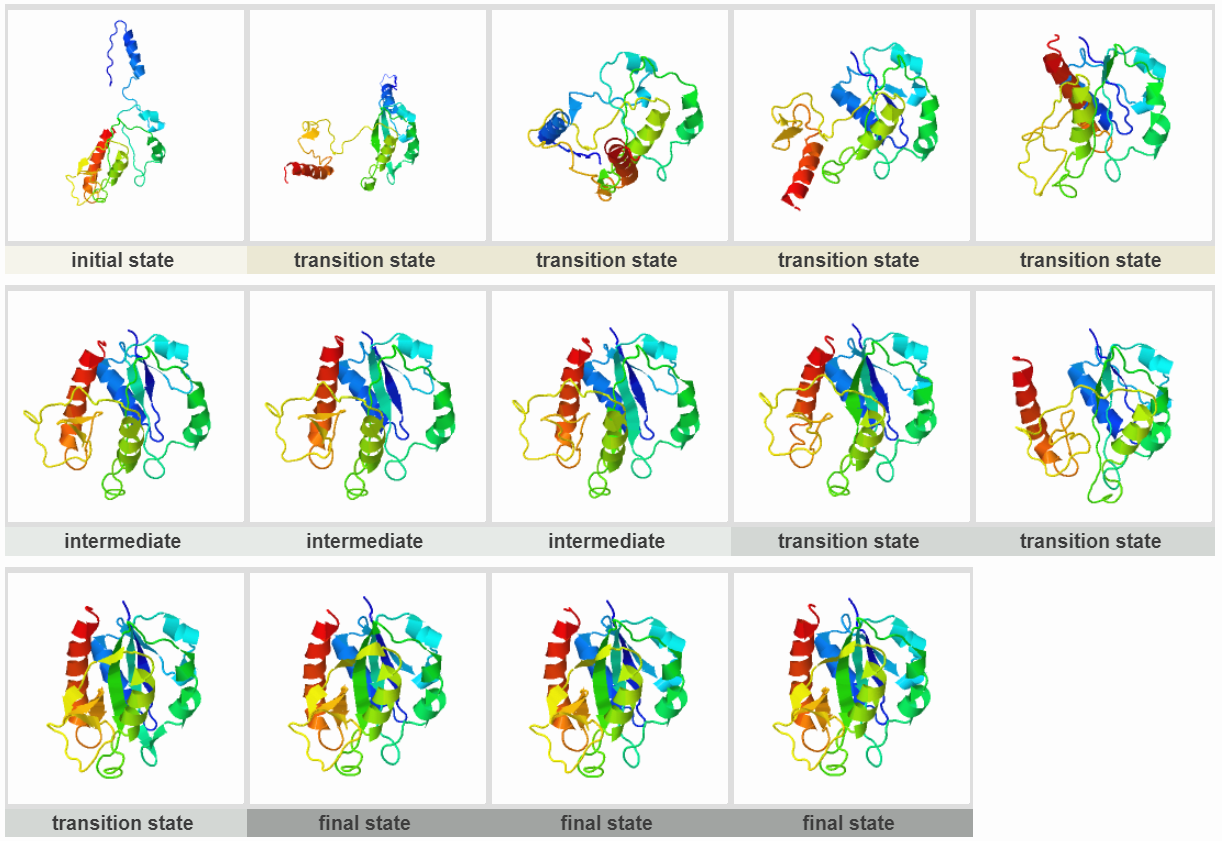


**Fig S9.** Flavodoxin (PDB ID:1FTG)


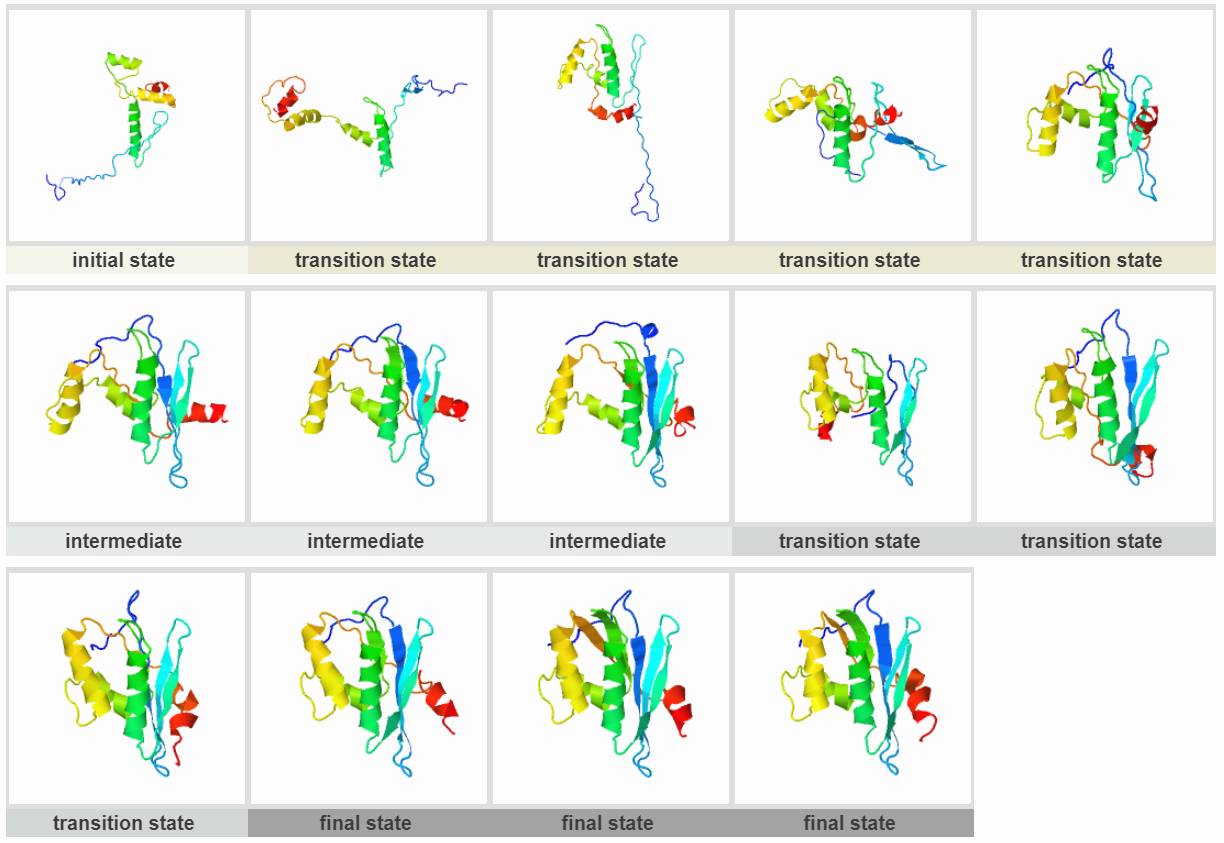


**Fig S10.** HIV-1 ribonuclease H (PDB ID:1HRH)


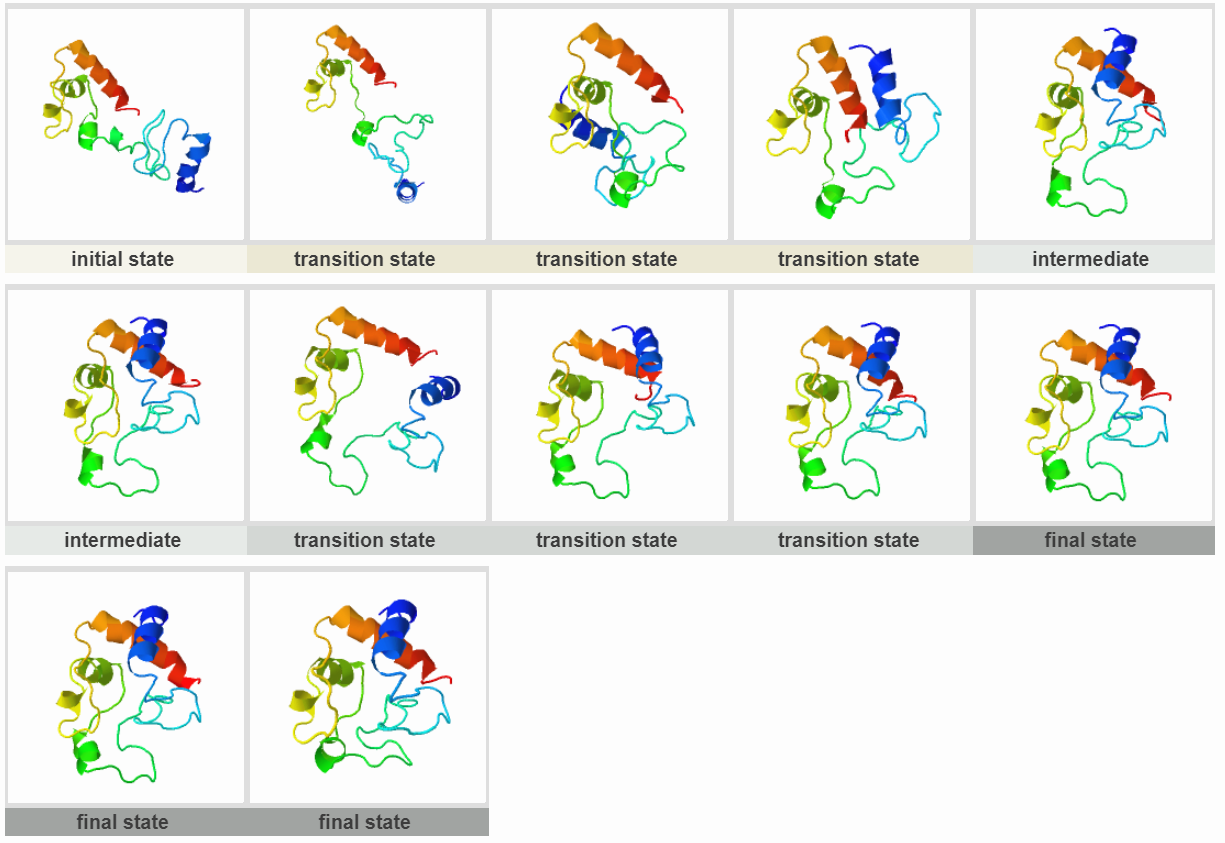


**Fig S11.** Cytochrome c (PDB ID:1I5T)


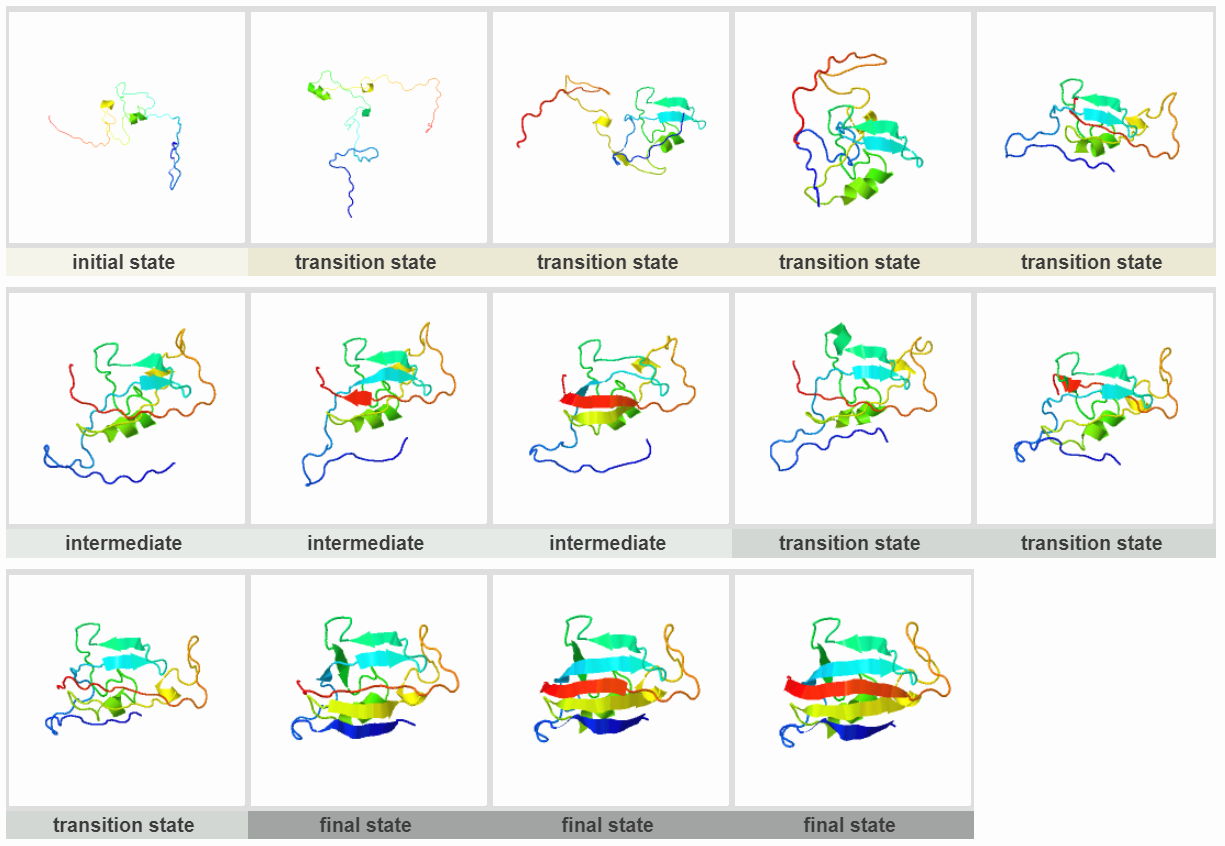


**Fig S12.** FKBP12 (PDB ID:1J4H)


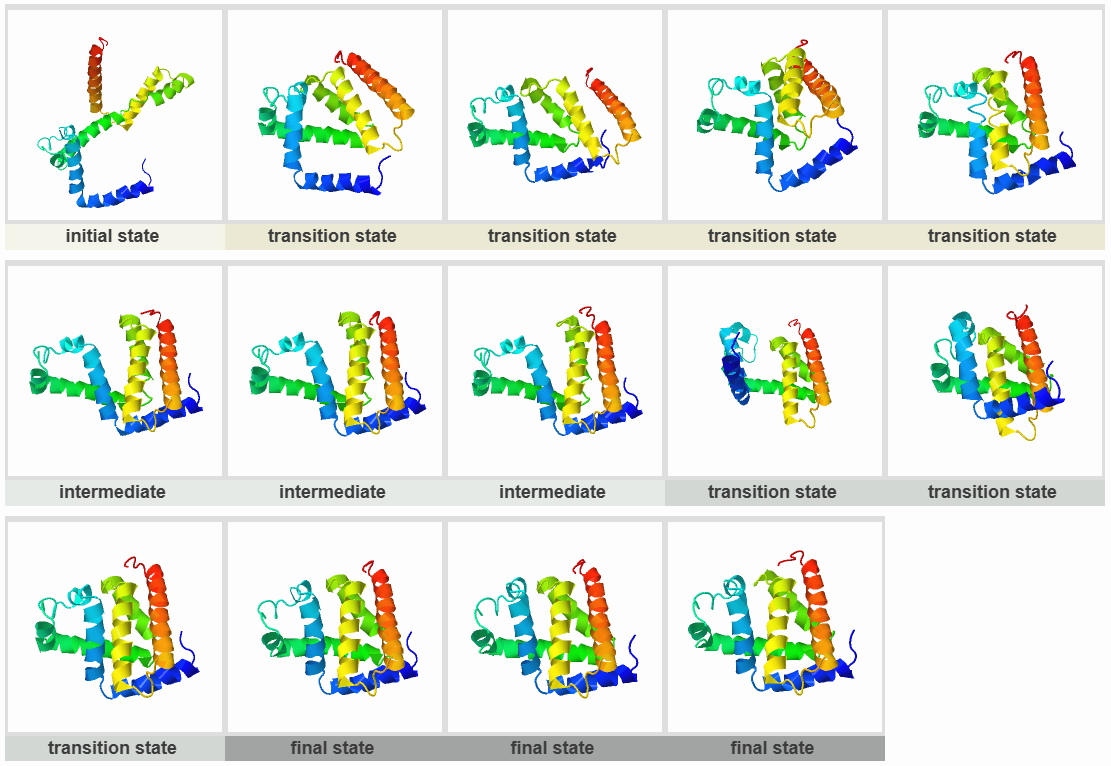


**Fig S13.** Apomyoglobin (PDB ID:1MBC)


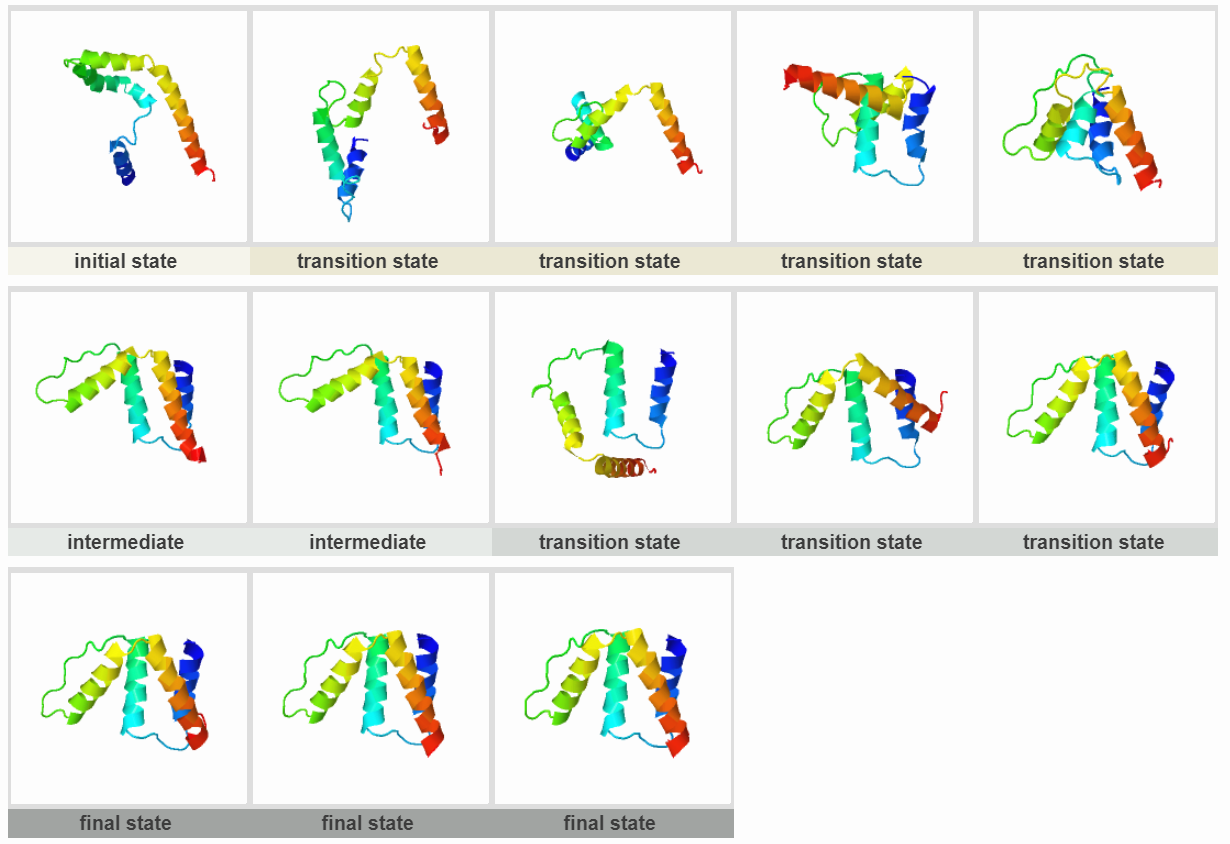


**Fig S14.** Acyl-CoA binding protein (PDB ID:1NTI)


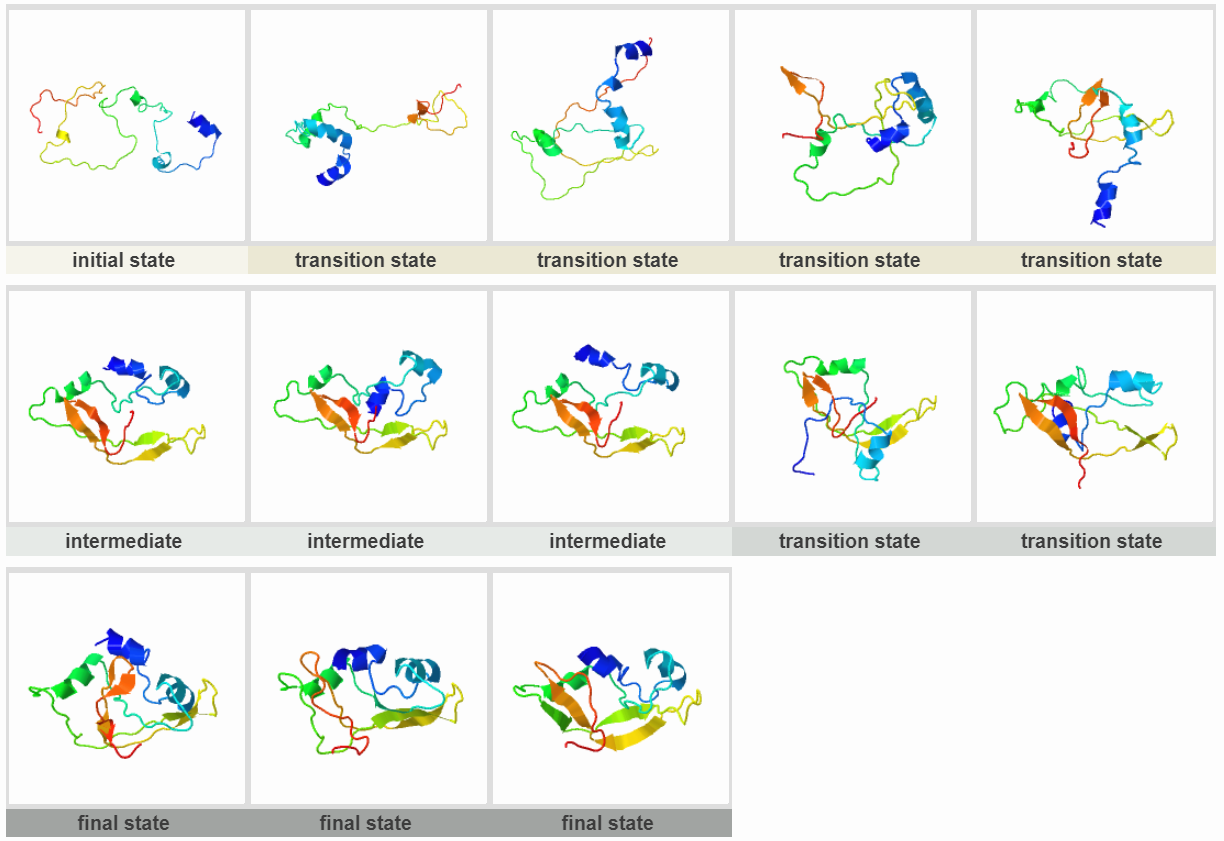


**Fig S15.** Onconase (PDB ID:1ONC)


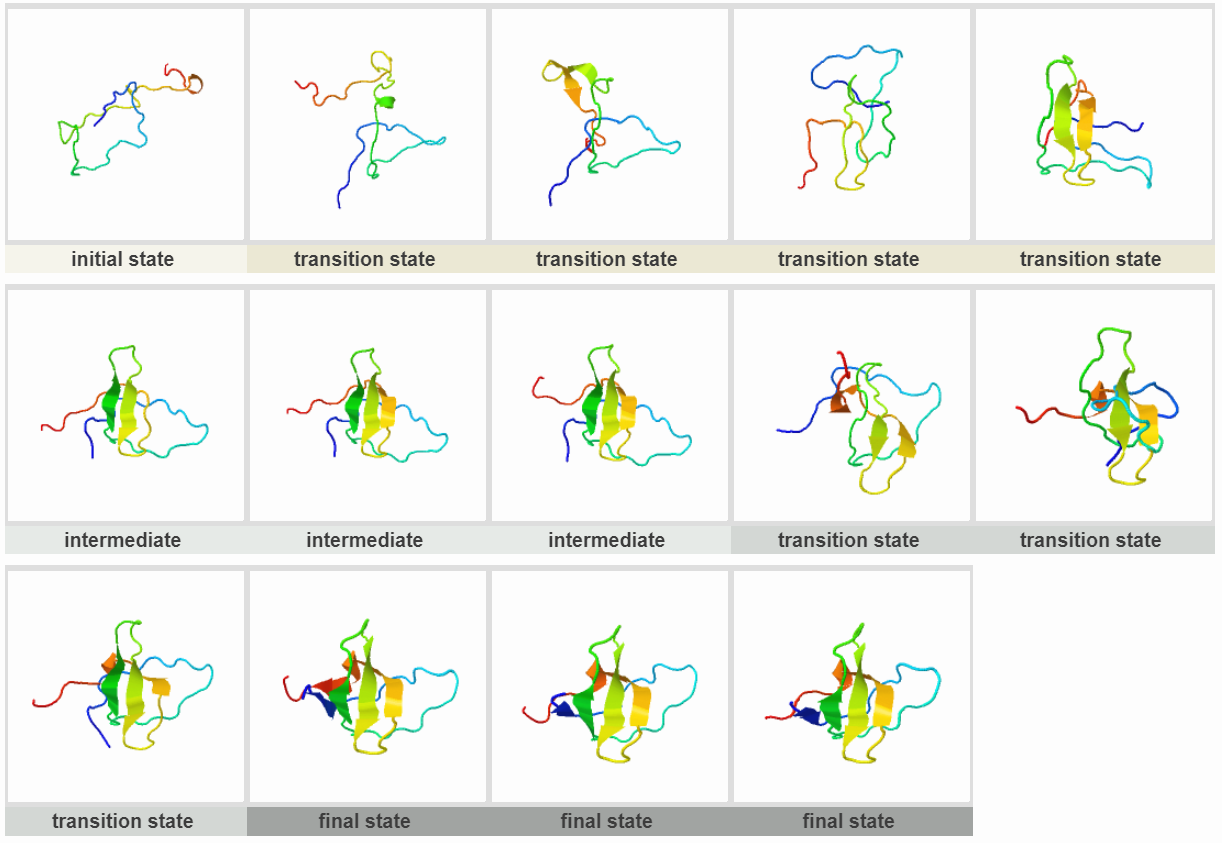


**Fig S16.** Fyn SH3 domain (PDB ID:1SHF)


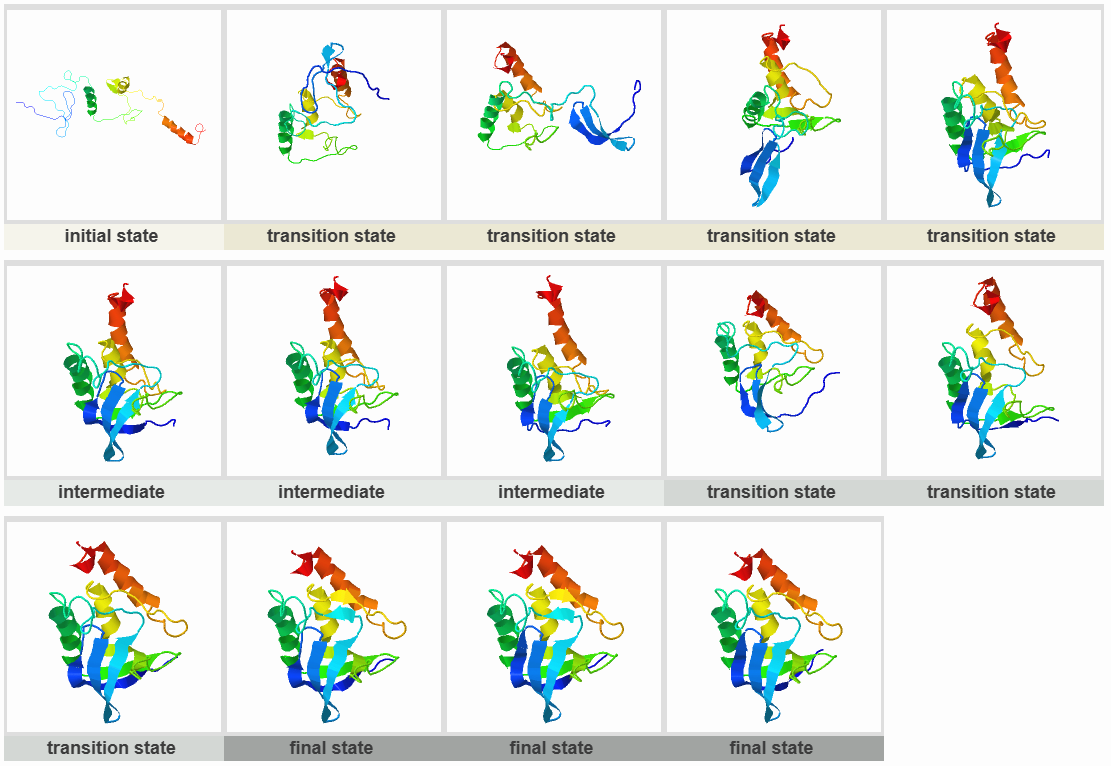


**Fig S17.** Staphylococcal nuclease (PDB ID:1STN)


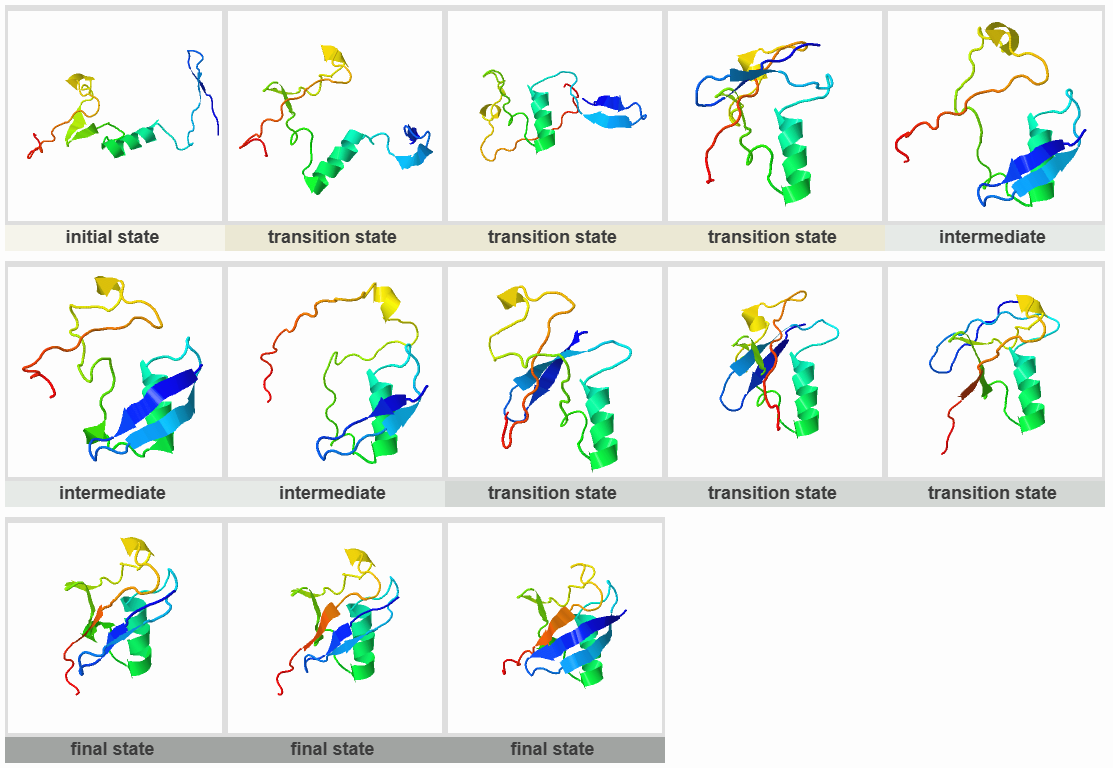


**Fig S18.** Polyubiquitin-C (PDB ID:1UBQ)


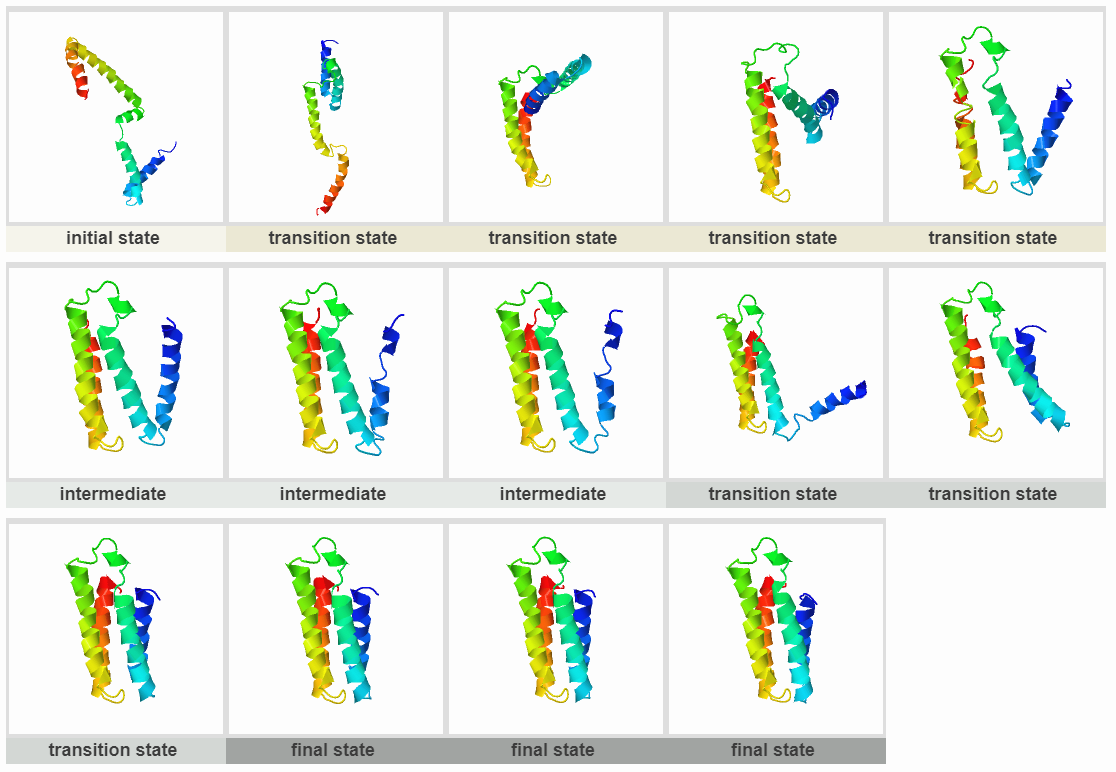


**Fig S19.** Rd-apocytochrome b562 (PDB ID:1YYJ)


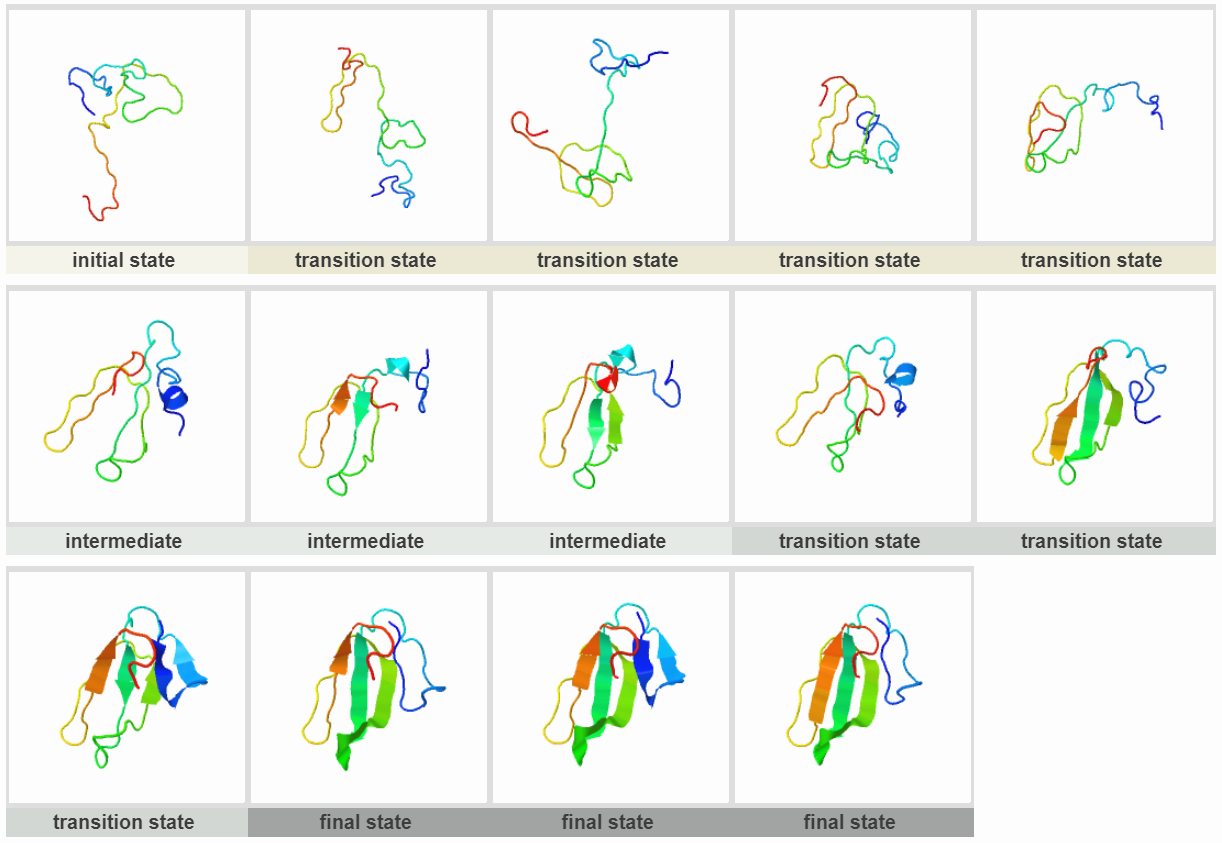


**Fig S20.** CTX III (PDB ID:2CRT)


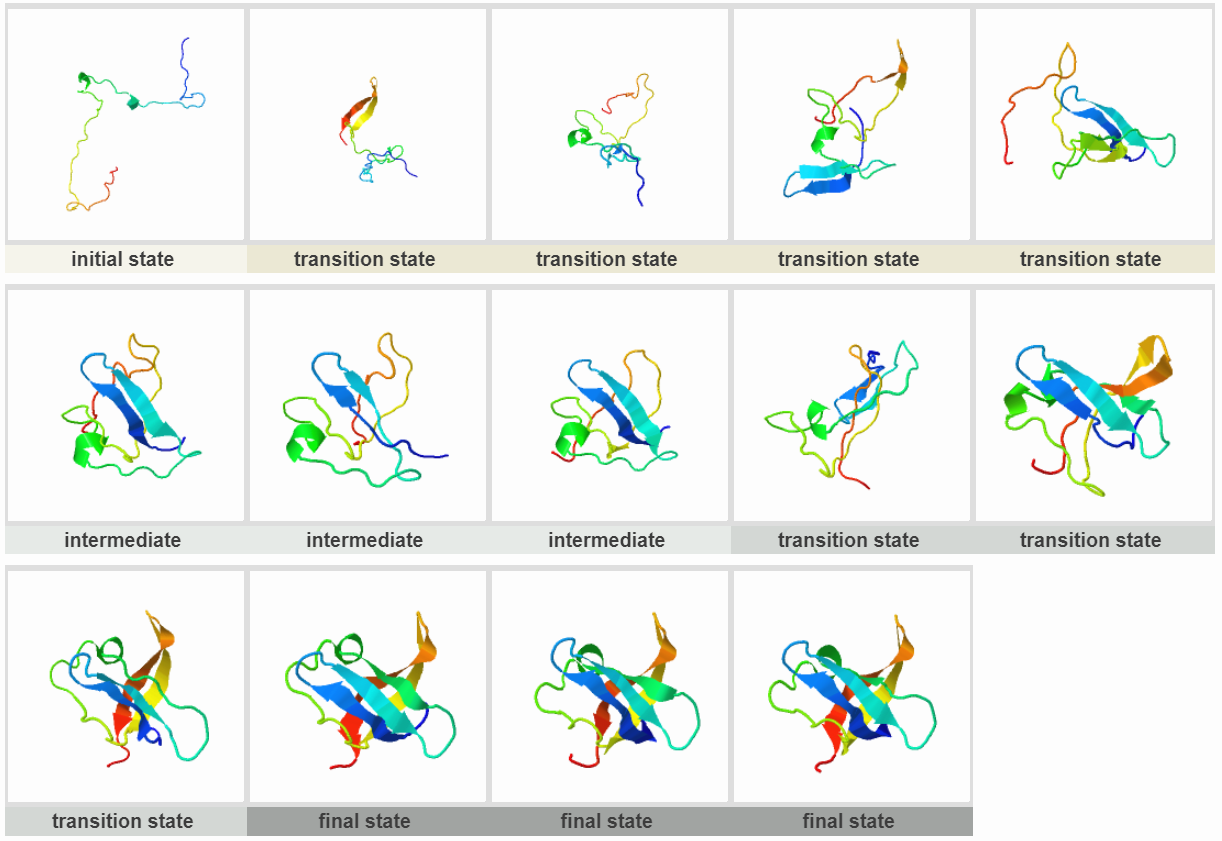


**Fig S21.** CspB (PDB ID:2F52)


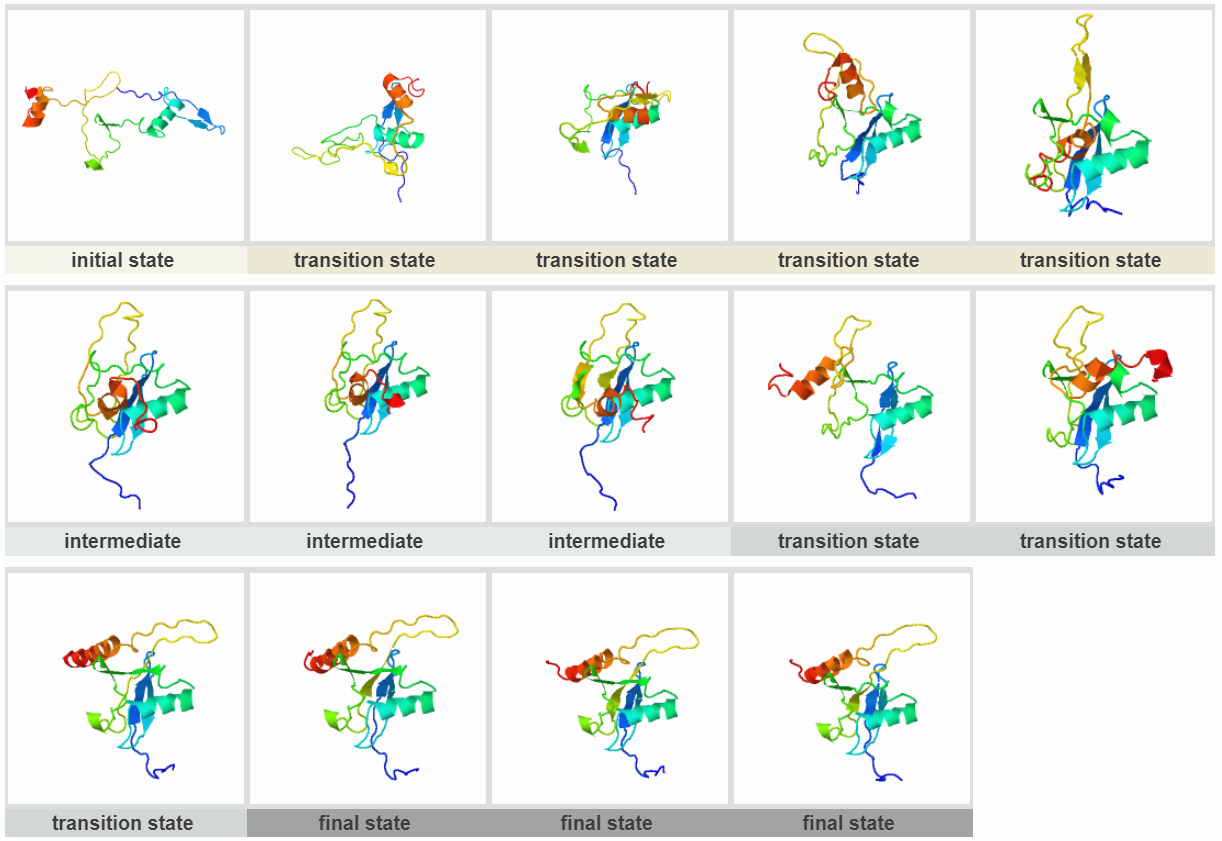


**Fig S22.** Ubq-UIM (PDB ID:2KDI)


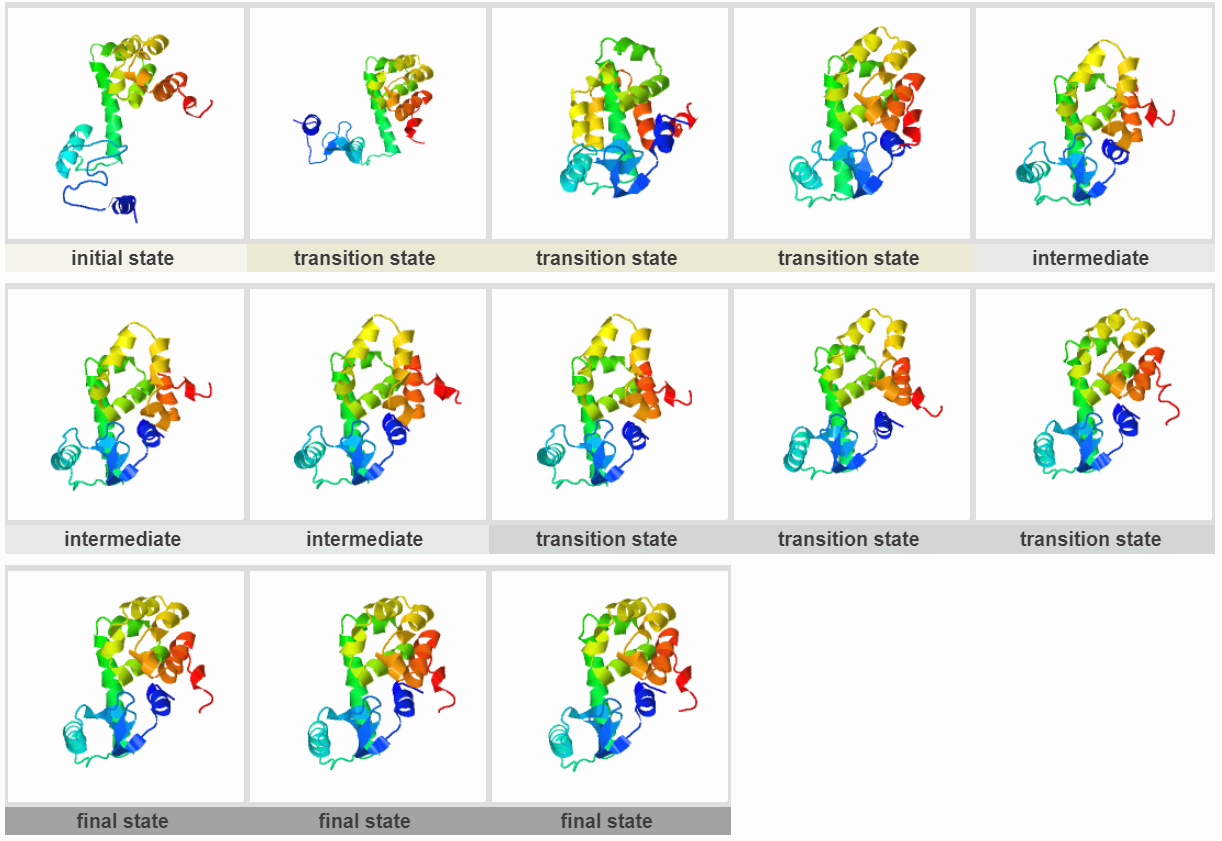


**Fig S23.** T4 Lysozyme (PDB ID:2LZM)


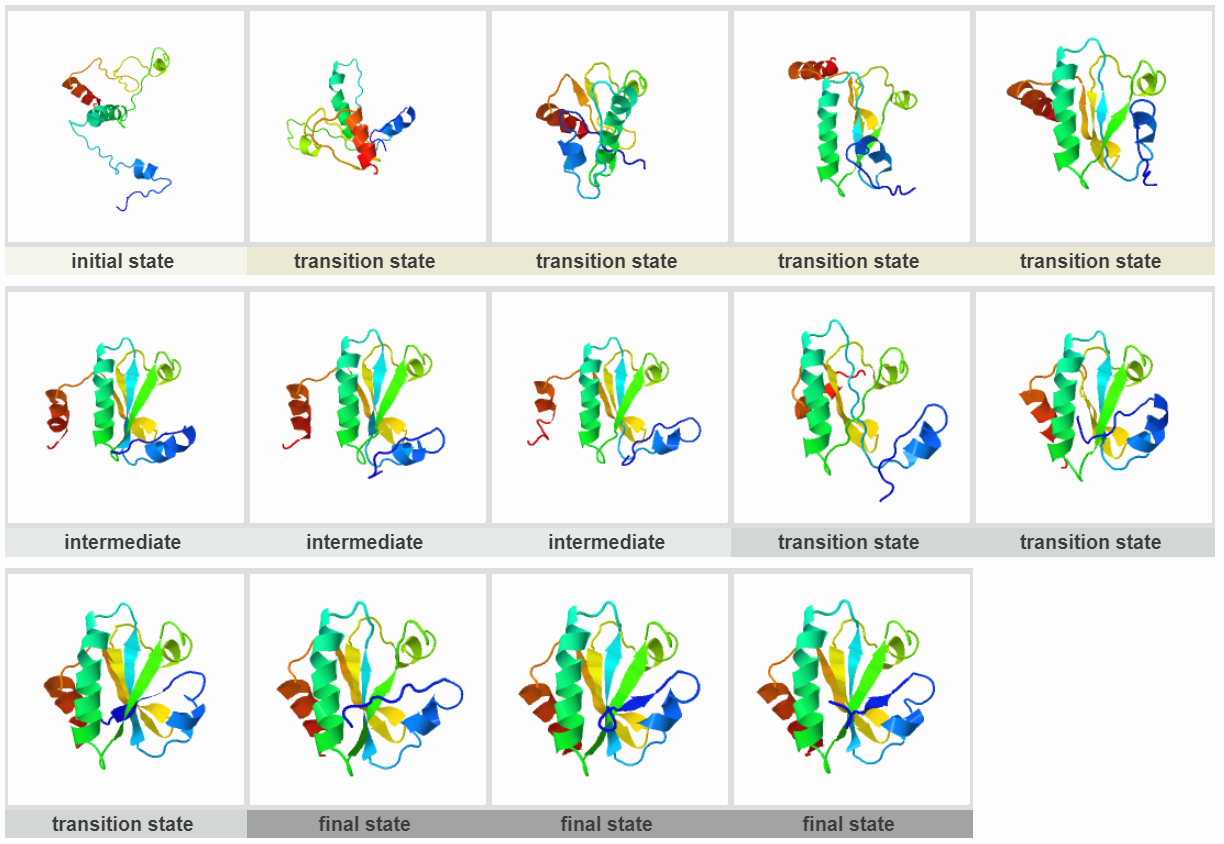


**Fig S24.** Thioredoxin (PDB ID:2TRX)


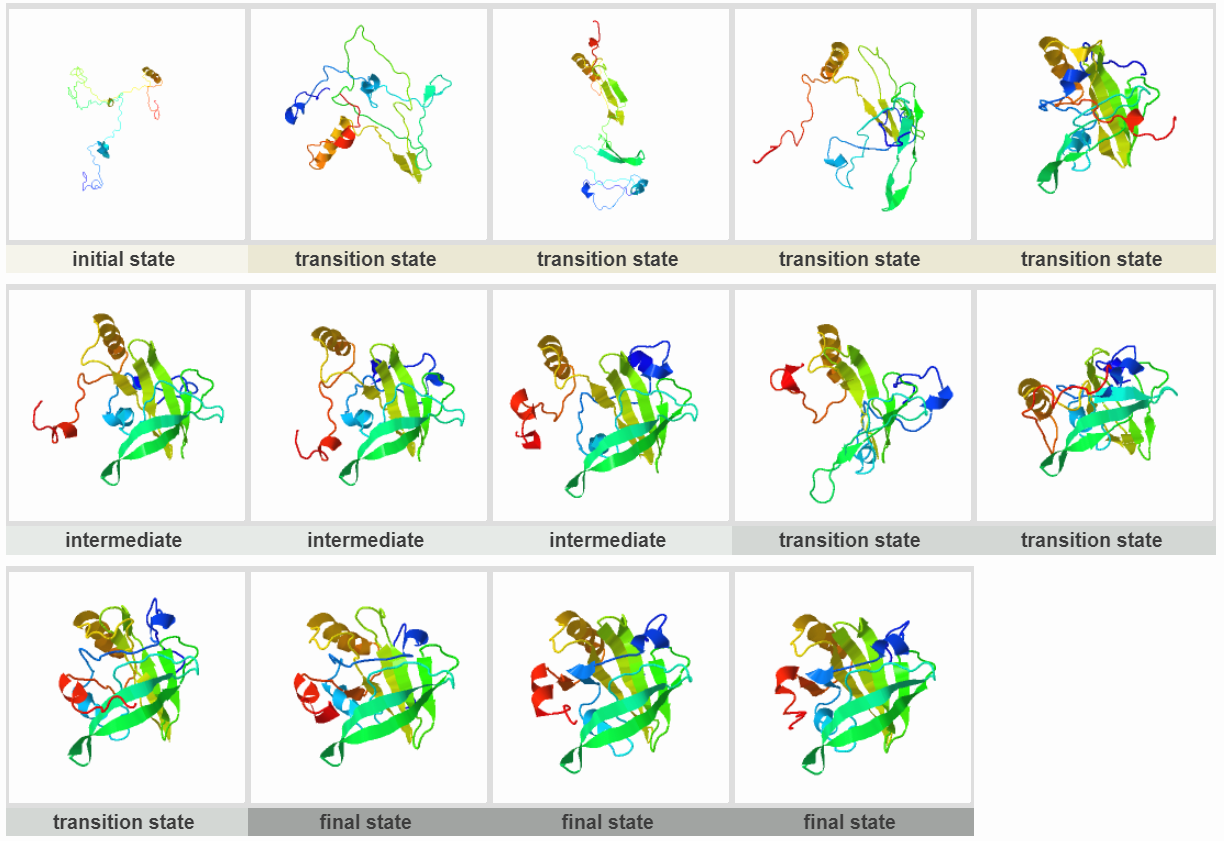


**Fig S25.** Beta-lactoglobulin (PDB ID:3BLG)


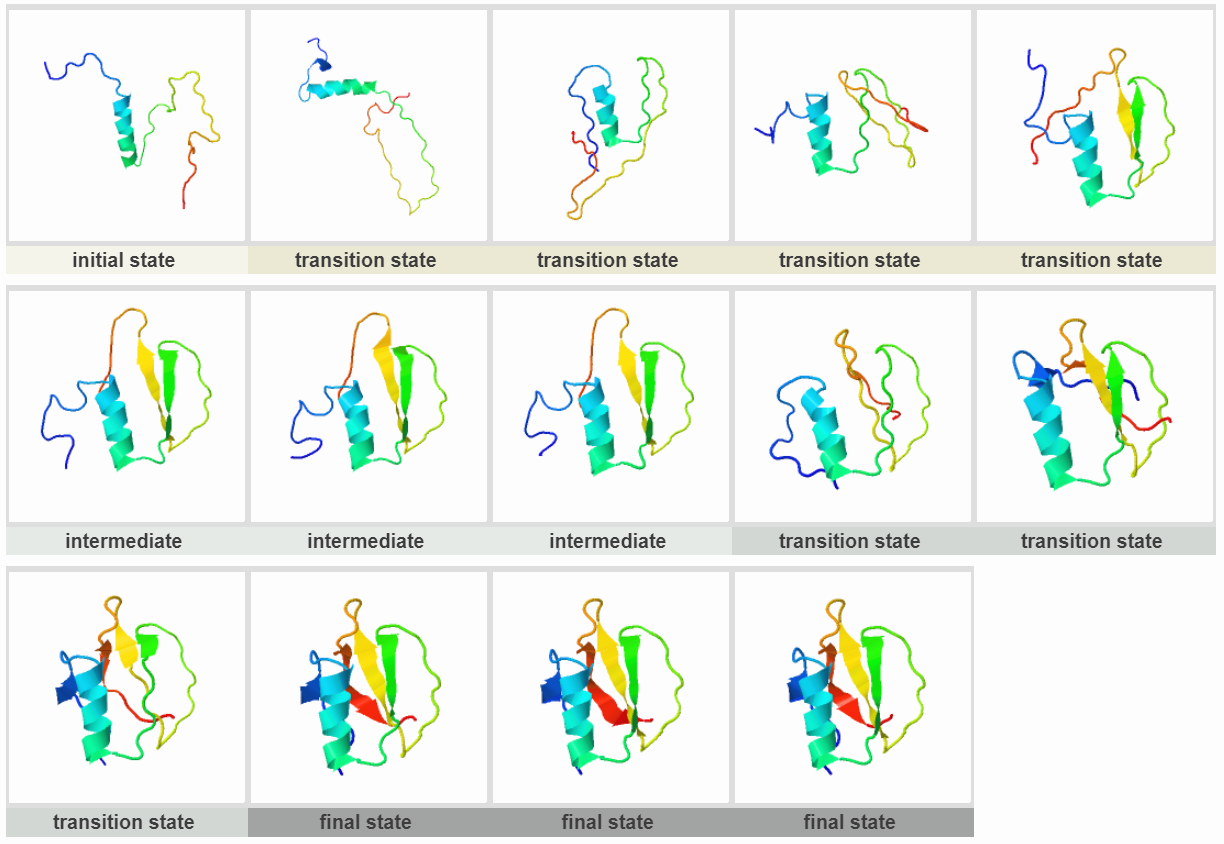


**Fig S26.** Chymotrypsin Inhibitor 2 (PDB ID:3CI2)


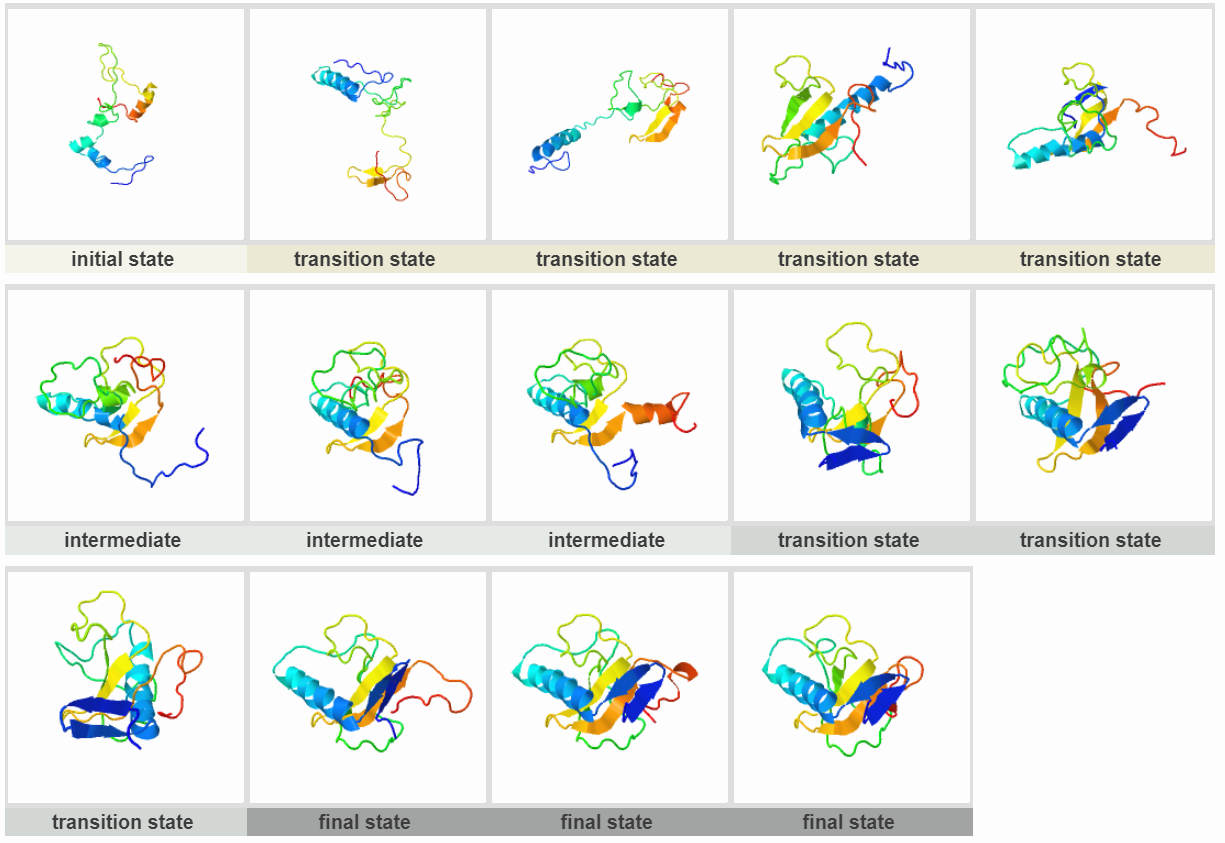


**Fig S27.** RNase T1 (PDB ID:3RNT)


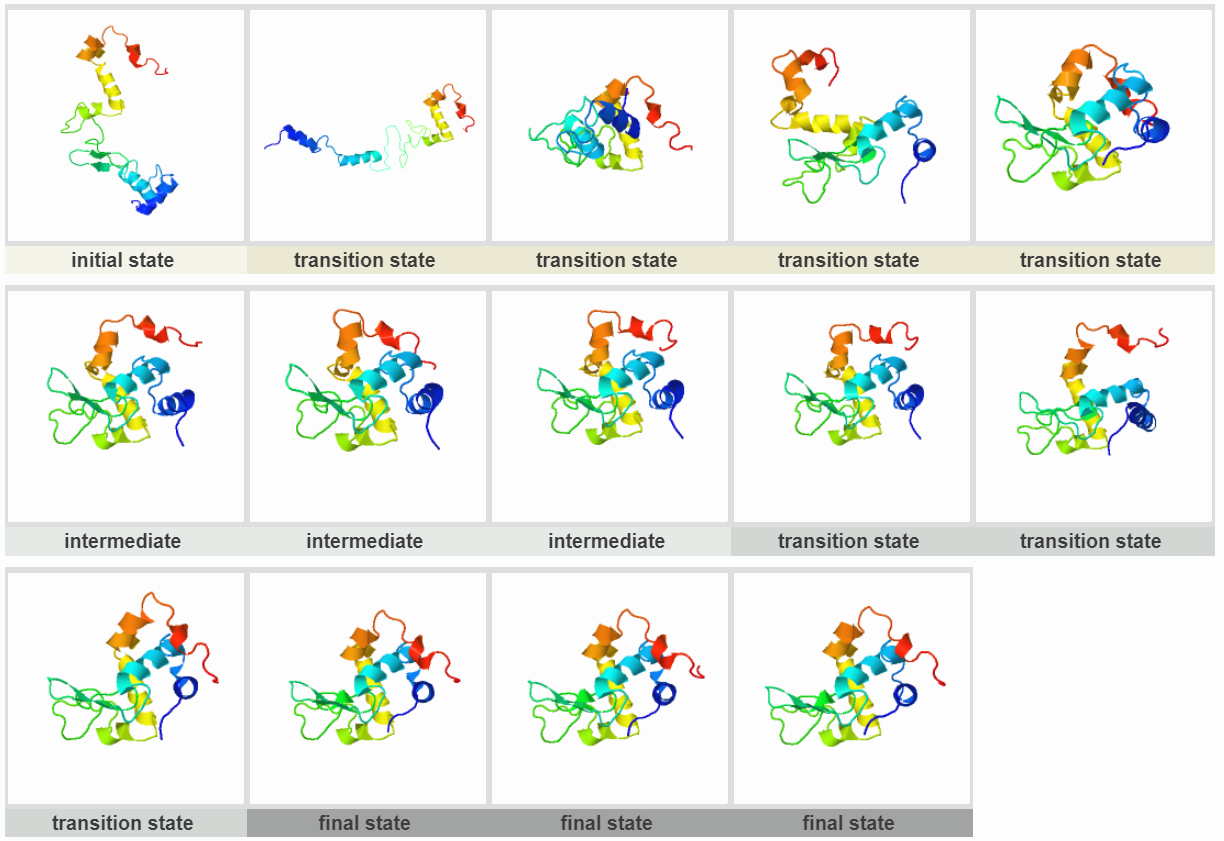


**Fig S28.** Lysozyme C (PDB ID:6LYZ)


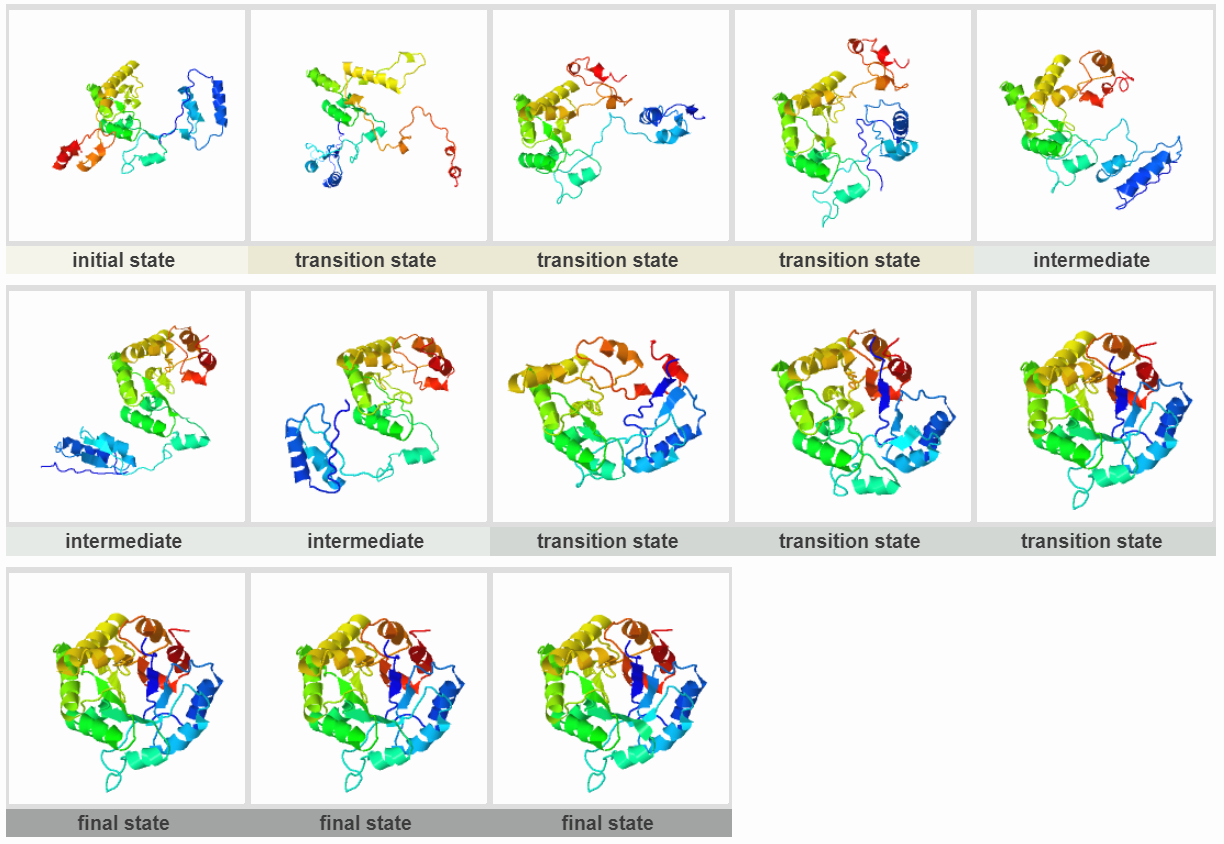


**Fig S29.** TIM (PDB ID:7TIM)


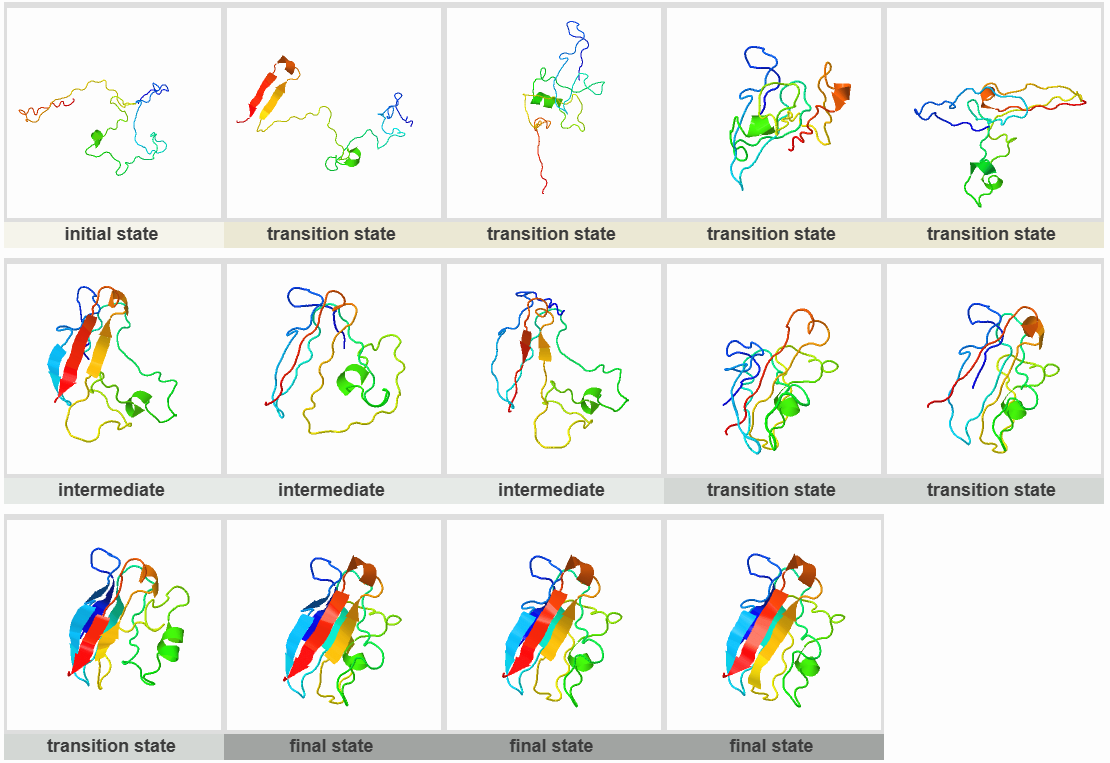


**Fig S30.** Plastocyanin (PDB ID:9PCY)


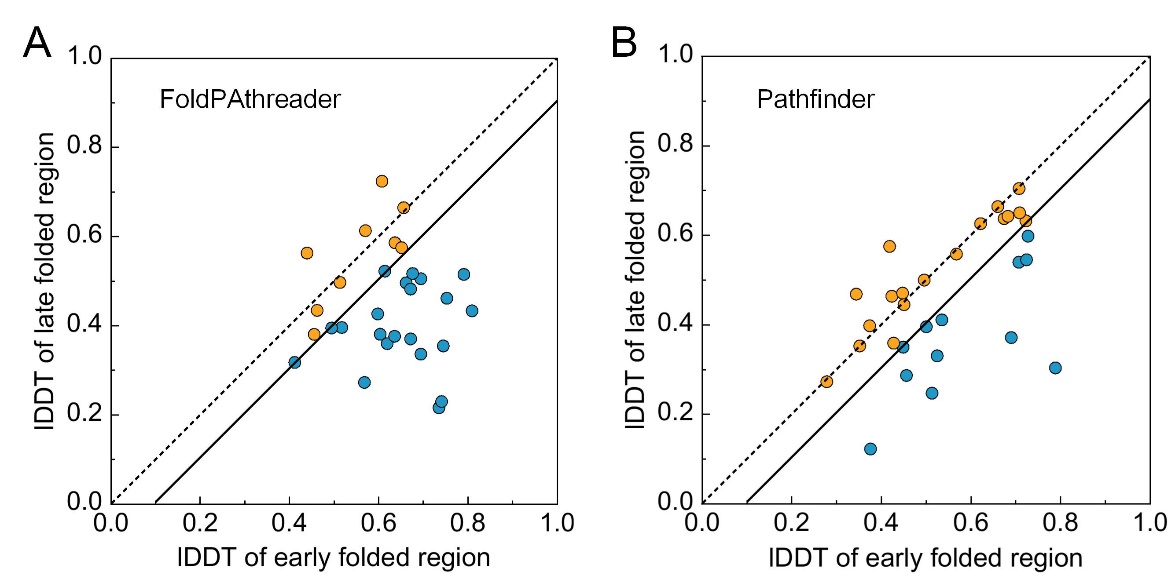


**Fig S31**. Head-to-head comparison between early folded region and late folded region of intermediates predicted by FoldPAthreader and Pathfinder. Blue circles are successfully predicted targets, yellow circles are failed targets.


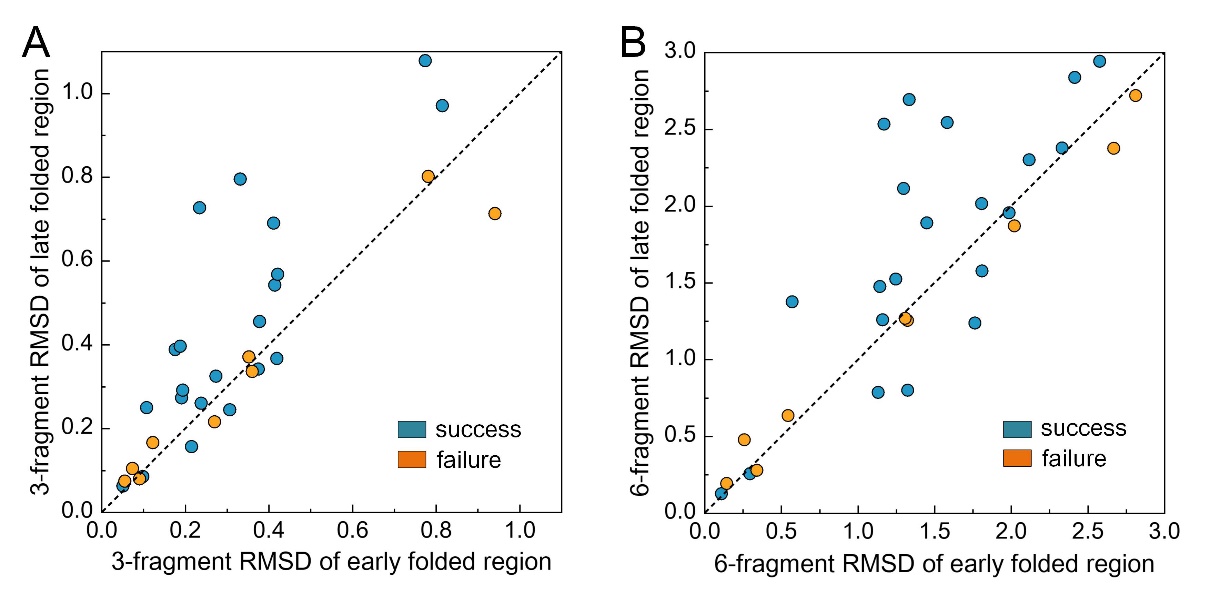


**Fig S32**. The average RMSD of 3-residue fragments and 6-residue fragments corresponding to EFR and LFR of 30 test proteins. Blue circles are targets successfully predicted by FoldPAthreader, yellow circles are failed targets.


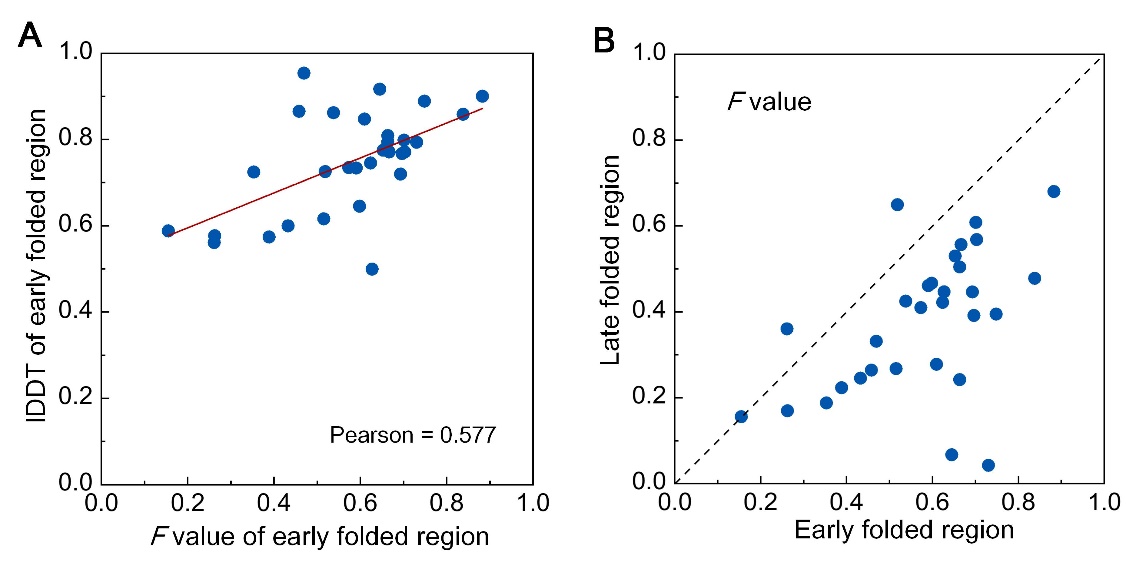


**Fig S33**. (A) The average *F* value of early folded regions versus lDDT of early folded region in predicted intermediates. (B) The average *F* value of early folded regions and late folded regions.
