# Supplementary material for: FoldPAthreader: predicting protein folding pathway using a novel folding force field model derived from known protein universe
Source: Genome Biol. 2024 Jun 11;25:152. doi: 10.1186/s13059-024-03291-x (PMC11167914; doi:10.1186/s13059-024-03291-x)
Supplement: Supplementary file 4 — Additional file 4: Review history. [file 13059_2024_3291_MOESM4_ESM.docx]

**1^st^ round**

**Reviewer 1**

Zhao et al. present FoldPAthreader, a protein folding pathway prediction method that uses homologous structures and a Monte Carlo fragment assembly strategy. I think this paper will be of interest to the community provided that the following comments are addressed.

\# Major

1. The 70% success number is mentioned in multiple places. How is it calculated? It could also be used in the ablation section, as it is not clear how to interpret Figure 7.

2. A number of quantities are mentioned that could also predict early folding regions (EFRs): sequence conservation, residue burial, hydrophobicity and the calculated F measure. These should be correlated or used to predict EFRs as baselines, otherwise there is a question about whether FoldPAthreader is superior to baseline methods for prediction.

3. The link between evolutionary history and the folding pathway and its appearance within FoldPAthreader is not convincing. It seems sensible to guess that local structural variability exhibited in the structural alignments correlates inversely to earliness of folding (the above baseline would help test this), but it is not clear how this links to the claim in the introduction about recapitulation of evolutionary history during folding. Most proteins in the structural alignment will not be part of the evolutionary history of the target protein, though they may be homologous. This claim should be toned down or an example provided where the specific evolutionary history of a protein is linked to its folding with FoldPAthreader.

4. The TMscore of 0.3 used to find structural homologs seems low and will bring in unlrelated folds. How was this number chosen and how is changing it expected to change the results?

\# Minor

5. The term folding force field is mentioned a few times, but it is not clear what it means so it could be clarified. Conventional MD force fields can fold proteins with only physical terms, at least in principle. It is not clear that the "proposed folding force field focuses on the folding process" any more than those. It is a physical and statistical force field with terms promoting native contacts based on variation in homologous structures. Perhaps it is the Monte Carlo sampling approach that makes it appropriate to study folding, but sampling is usually referred to as separate from the force field.

6. It is worth discussing how the F measure could be biased by the global structural alignment. For example, a two domain protein could align primarily on either domain with TMalign, with apparent larger distances in the non-aligned domain. Would it be possible to replace F with sequence conservation?

7. Is there any correlation of Monte Carlo steps to experimental folding time? Do known fast folding proteins reach the native state faster?

8. Conventional MD successes such as the Shaw lab 12 proteins work could also be mentioned in the introduction.

9. Figure 3: summary statistics could be shown, such as the mean values or a histogram/violin plot of the two distributions.

10. Figure 5: the Rg y-axes should be the same across the two plots. I also found the ordering of a-h to be confusing, these could increase left to right.

11. Figure 6: the caption needs more detail, e.g. which protein is shown. It is not clear how the structures in A relate to those in B, there might be a better way to show this.

**Reviewer 2**

In this work, the authors introduce FoldPAthreader, an interesting fold pathway prediction method that allows to investigate the potential folding pathway of a protein of interest. It builds on the recent advances in protein structural bioinformatics and addresses one of the major challenges of the field: how does one protein chain go from a disordered chain of amino acids to an ordered 3D fold. The method is conceptually interesting and of relevance for the field. The paper is written clearly but I have major comments, particularly related to unclarity (that in some cases may come across as over statements) and benchmarking. Please see my comments below:

Major:

- in the overview, some steps are not clear. For example, in line 101, it is not clear what the fragment library is, how it is generated and how it is used. It would be nice and clearer if the same level of detail given to the other features (eg, residue frequency score) is given here. This would help, for example, understand how they relate to the folding information used later. Actually, the fact that they are not explained well here, makes it that some later parts in the results and discussion are harder to interpret/understand.

- In line 108, how different were the experimental setups? are the experimental conditions the same, similar or drastically different? could differences in experimental conditions explain the cases where FoldPAthreader did not preform well?

- In line 136, the lDDT of the different regions was computed using what as a reference? The target (AlphaFold) fold or the final fold generated by FoldPAthreader? If the latter, as the method generates an ensemble, which member of the ensemble was used? It is difficult to follow this paragraph, actually, as it is not clear at which state of the "simulation" these pLDDTs are being computed too. Please clarify this paragraph, it will further allow to better understand the work.

- in line 166, the authors have to be careful when stating that the ensemble may reflect inherent flexibility without providing alternatives. I think what it actually reflects is the uncertantity of the method. There is no data provided by the the authors that supports that the ensemble is biologically relevant, which is a must to conclude that the ensemble may represent biologically relevant states.

- in line 170, the authors make, in my opinion, a fallacious statement. They write that the results support the hypothesis. This would be true if the method to get the results would not be driven by the hypothesis. The authors developed a method that is fundamentally based on and driven by the hypothesis, so it is to be expected that the results follows it. I believe what the authors want to write is that the benchmark of the method indicates that the principles they based their method on can be applied to the test cases, giving support to the method. But they can not generalise it. Only 70% of the cases matched the results of the method and their dataset is small, not covering all possible paths proteins can follow to fold. So I would recommend the authors rephrase this part, clarifying it for the naive reader. This should also be addressed in the discussion, although the last part of the comment was already addressed.

- The authors often over-interpret the results of their method. One example is the point above about line 170. Another is in line 251, where the authors write that the results of their method indicate that there are different priorities for proteins of different secondary structure composition. Maybe that is the case but I don't think the authors provide enough supporting data. They are tacking this particular conclusion based solely on the results of the method and not benchmarking against experimental data (at least at this specific stage). In this line, I believe the maximum that they can conclude is that their method deals with different structure compositions differently. If this describes biologically relevant behaviour, that should be evaluated with some kind of benchmarking. The authors should make clear what they mean in this part, particularly when it can misinterpreted.

- how does the method work with folding-affecting mutations? The authors refer in the intro that some point mutations can impair the folding pathway and promote aggregation, leading often to disease. But do not address this point in their results or the method, I would expect that the method is insensitive to point mutations that affect the folding, as it is based on AlphaFold models and patterns from MSAs. It would be important to address this issue, if not in the results by investigating the results of known variants (which can be easily obtained from UniProt) at least in the discussion.

- the authors show the predicted folding pathway for two wildly studied proteins, BPTI and TIM. These are not only widely studied from a folding perspective, but they are also quite widespread in nature and soluble. Therefore I would expect their MSAs to be quite deep and there are a lot of structures (predicted and experimental) for them. This raises the questions: (1) how does the method work for rare proteins that are predicted at high accuracy with AlphaFold? And (2) how does it deal with transmembrane proteins and those that require chaperones? These points should be addressed at least in the discussion.

- There is no benchmarking against other methods. The paper only covers the method described and does not compare it with other folding pathway prediction methods, which are mentioned in the introduction. In order to evaluate the quality and relevance of the method, it is important to have a point of comparison.

- The web resource is an easy way of using the method. I find it unclear in the main page which method is used when submitting a folding pathway prediction job. Is it FoldPAthreader or PAthreader? This is only clear after selecting the option to predict a folding pathway, but then it seems it is not possible to run PAthreader. It is also not clear from the main text how the two methods differ. It would be important to clarify this and include, at least, PAthreader in a benchmark against FoldPAthreader if possible.

Minor:

- The authors write "The protein folding problem has emerged as a new challenge with the significant advances in deep learning driven protein structure prediction methods.", while this is relatively true, it is misleading. The protein folding problem is an old problem, it did not emerge now. What is unique to the current times is that with the protein structure prediction problem "solved" thanks to deep learning methods, it becomes now a reachable problem to assess using deep learning.

- line 84: the authors write that the AlphaFold database is the most abundant structure database but earlier mention ESMfold database, which has 3x more models than AlphaFold DB. Why did the authors not use ESMfold database?

- in line 176, could the authors write Haeckel's statement here too and not only reference it? It would facilitate reading this paragraph.

- in line 178, "the evolutionary" should be "the evolution" or "the evolutionary history"

- in line 195, why TM-score > 0.3? Anything below 0.5 is not a significant similarity...

- this is just a very minor comment, and is completely up to taste, but I find the animation of the PAthreader logo in the web resource distracting.

**Reviewer 3**

The question addressed in this work is with no doubt of high interest. The method is interpretable. Moreover, it opens very many questions on how to go further with respect to what the authors do, at large scale.

The idea is simple. The authors leverage on existing AF2 structures to produce a library of protein fragments, a physical potential energy function and a statistical potential energy function leading to the reconstruction of the folding pathway.

Their hypothesis is that the conformational changes that proteins undergo when they fold from a disordered state to a specific three-dimensional structure reflect the evolutionary history of the proteins and it should be decoded from sequences. This idea is not new but it is used here in a very successful way. Namely, it reminds me of the usage of coevolution analysis to explain "how coevolving clusters of amino-acids can help to identify stable subparts of the protein sequence in the folding process" for protein A discussed in (Dib, L., & Carbone, A. (2012). Protein fragments: functional and structural roles of their coevolution networks. PLoS One, 7(11), e48124). I cite especially this work because, crossed with what the authors do, coevolution analysis might help them to go further towards a large-scale development of their method.

The method has been tested on 30 known structures. The ensemble of proteins is limited because experimental data on folding pathways reconstruction is also limited. This dataset is definitely biased in terms of the stability of the structures, the lack of long/short unstructured loops, intrinsically disordered regions, and regions that undergo disorder-to-order transitions after complexification. Having said this, it is reasonable to start from well-structured proteins and the authors should just discuss explicitly this point in their article instead of proposing the method as resolving a general problem. In my opinion this method has the clear merit to advance the question but it is not the final work on the question when large-scale prediction has to be approached.

Inaccurate sentences in the abstract should be changed:

Line 10 - "While the structures of almost all known proteins have been successfully predicted, the process by which they fold remains an enigma"

Here we do not speak about "Known proteins" but about "proteins known to have a structure". The large amount of disordered protein sequences is neglected by the interests of this method.

Line 15: the authors use the word "folding mechanism" but I do not see any mechanism that is unraveled in this article. Do they want to speak about "folding pathways"?

This wording should be systematically replaced in the full manuscript. If I miss something, this means that they should addressed explicitly the idea of "mechanism" in a convincing way.

In the background section, line 43, the authors mention molecular dynamics. There is a recent work on Alpha Flow and ESMFlow (https://openreview.net/pdf?id=yQcebEgQfH ; Jing, B., Berger, B., & Jaakkola, T. (2024). AlphaFold Meets Flow Matching for Generating Protein Ensembles. arXiv preprint arXiv:2402.04845) that should be referred to in the introduction or in the discussion. Again, this work can be useful to the authors to go further towards a large-scale development of their method.

Line 87 - Figure 1. The labels U, TS, I, N are not defined. I guess they stand for Transition state (TS) and intermediate (I) states. Please define the four states in the text.

Line 130. The author say "It is observed that the aligned structures of the folding intermediates ensemble are consistent with preferentially folded blue structures…". You need to use quantitative measures. Please replace this sentence with quantitative evidence.

Line 134. "the results suggest that FoldPAthreader has the ability to predict the folding pathway for general proteins, and is not limited to the type and size of proteins.". as mentioned above, the size of the protein is not the only important feature to evaluate difficulties in the folding pathway reconstruction. Disordered regions, loop structures, weakly stable regions are also potential components. The authors should provide support for this statement with much more precision. This effort will establish the limits of the analysis. As mentioned setting these limits will provide a clear view on the work.

Line 146. It would be interesting, and fundamental I guess, to have some evaluation of the variability on the fragments in the library which are used to determine the folding pathways of a protein. How much variability on these fragments exist for pathways that are successfully predicted? We might have the impression that successful predictions are linked to a restricted space of fragment conformations, leading the dynamics. Is it the case?

Line 155. Figure 2. It would be interesting to have a color scale in the predictions that fits the time of formation of the structure, as given for the experimental folding order with blue-grey colors. Could you please replace the present rainbow scale colors with something meaningful to this work? Folding order is what I would expect to see.

As a general principle, please add color scale bars to your figures and explain them in the legends.

Line 155. Figure 2. You say "Their partial structures with high overlap are marked with β-sheet or α-helix, indicating preferential formation." How the overlapping is quantitatively defined? High vs low? Can you be more quantitative?

Line 160. Figure 3B. are the blue square defined wrt existing secondary structures? How are they identified?

Figure 3C. please provide the intuition on small and large differences in IDDT for early/late folded regions. To what is it due? Is IDDT really a good/the right measure to discriminate difficult situations?

Line 172. The fact that the structural space of the known protein universe can be used to study most protein folding pathways is an important point that is made by this work. The authors state the idea as "the structural space of the known protein universe is complete and can be used to study most protein folding mechanisms". As mentioned above they cannot speak about mechanisms and they cannot claim that the space is "complete". They do not prove this.

Line 168. The authors speak about fragments but I had to go in the Methods to discover that their length if either 6- or 3-aa in length. It would be better to address their characteristics from this point in the manuscript. Moreover, I found that more statistics should be provided about fragments. Are they fragments that are found more than others in structures? What is their aa composition? …. There are a number of important questions that can be addressed on them to provide some intuition on their role.

About their length. Why 6 and 3? Is there some meaning about the role that fragments might have compared to 6-fragments?

Can you add in table S4 two lines reporting the performance with the 6-fragment library only and the 3-fragment library only?

There is a lot of work done on structural alphabets, from more than 20 years now and there is no mention of these works in the article. They should be mentioned and discussed in the light of the differences introduced here:

Camproux, A. C., Brevern, A. G., Hazout, S., & Tufféry, P. (2001). Exploring the use of a structural alphabet for structural prediction of protein loops. Theoretical Chemistry Accounts, 106, 28-35.

de Brevern, A., Camproux, A. C., Hazout, S. A., Etchebest, C., & Tufféry, P. (2001). Protein structural alphabets: beyond the secondary structure description. Recent research developments in protein engineering, 319-331.

Etchebest, C., Benros, C., Hazout, S., & de Brevern, A. G. (2005). A structural alphabet for local protein structures: improved prediction methods. Proteins: Structure, Function, and Bioinformatics, 59(4), 810-827.

Tyagi, M., De Brevern, A. G., Srinivasan, N., & Offmann, B. (2008). Protein structure mining using a structural alphabet. Proteins: Structure, Function, and Bioinformatics, 71(2), 920-937.

Pandini, A., Fornili, A., & Kleinjung, J. (2010). Structural alphabets derived from attractors in conformational space. BMC bioinformatics, 11, 1-18.

Craveur, P., Joseph, A. P., Esque, J., Narwani, T. J., Noël, F., Shinada, N., ... & de Brevern, A. G. (2015). Protein flexibility in the light of structural alphabets. Frontiers in molecular biosciences, 2, 20.

Important: The fragment library should be made available. It is not clear if it is.

Line 175. Big claims are made here. The idea is then proved on a single example + 4 more! The text should be rephrased.

Generally, there are limitations of the method that are not enough discussed. For instance:

- What is its dependence on the size of the protein, its conservation in the tree of life?

- Can the method predict folding pathways for new proteins?

- What is its performance on flexible regions? Compared to more conserved ones?

- does the method observe situations where unfolded parts undergo states of disorder-to-order-to-disorder transitions in the dynamics they reconstruct?

**Authors’ response**

***Referees 1 and 2 ask you to justify the TMscore cut-off of 0.3, and Referee 1 asks you to explore the effects of varying the cut-off.***

**Response:** When the TM-score is too high, the similarity and difference of local structures cannot be reflected in the structure alignment, making it difficult to capture structural conservation and folding information. When the TM-score is too low, irrelevant structures are included to the structure alignment, resulting in possible errors in folding information. In this work, we selected structures with TM-score greater than or equal to a given threshold for multiple structure alignment. This threshold is determined through experiments, and the results are presented in **Table R1**. It can be seen that when the threshold is 0.3, the number of prediction results that consistent with the experimental data is the largest.

**Table R1:** Prediction results at different TM-score thresholds.

|  | num (consistent with experimental data) | lDDT of early folded region | lDDT of late folded region |
| --- | --- | --- | --- |
| TM-score = 0.1 | 18/30 | 0.672 | 0.473 |
| TM-score = 0.2 | 20/30 | 0.680 | 0.476 |
| TM-score = 0.3 | **21/30** | 0.681 | 0.474 |
| TM-score = 0.4 | 19/30 | 0.692 | 0.499 |
| TM-score = 0.5 | 16/30 | 0.695 | 0.514 |

***Referee 2 asks you to compare with other methods, including PAthreader.***

**Response:** We added experiments to compare predicted folding pathway with Pathfinder on 30 test proteins. Pathfinder is a protein folding pathway prediction method that explores the transition probabilities of folding intermediate states through conformational sampling [R1]. But we cannot compare with other methods because their codes cannot be localized or folding intermediates cannot be predicted.

The results of FoldPAthreader and Pathfinder are shown in **Figure R1**. Red areas represents that the LDDT of the early folded region is 10%(green) higher than that of the late folded region. The cases in red areas indicate that the folding order is consistent with biological experimental data. On 30 proteins, FoldPAthreader successfully predicted 21 proteins whose intermediates were consistent with biological experimental data, and Pathfinder successfully predicted 12 proteins. The average IDDT of early folding region and late folding region are 0.681 and 0.474 for FoldPAthreader, and 0.568 and 0.479 for Pathfinder. These results show that the performance of FoldPAthreader is significantly better than that of Pathfinder. We have added these to line 183 of the manuscript and the supplementary information.

**Figure R1**. Head-to-head comparison between early folded region and late folded region of intermediates predicted by FoldPAthreader and Pathfinder. Blue circles are successfully predicted targets, yellow circles are failed targets.

In addition, based on suggestions of Referee 2, we have modified the homepage of the web server, as shown in **Figure R2**. It contains two modules: folding pathway prediction (FoldPAthreader) and remote template recognition (PAthreader), and they can both work normally. PAthreader is a remote template recognition method that uses three-track alignment to thread PDB and AlphaFold DB libraries. Based on the identified remote homologs, PAthreader initially explores the folding order of protein using residue frequency distribution and secondary structure information. This process requires artificial thresholds to divide the folding order. Compared with PAthreader, FoldPAthreader extracts folding information from multiple structure alignments to construct a folding force field, and predicts folding pathway through conformational sampling, which does not require manual intervention. Furthermore, FoldPAthreader quantified the accuracy of folding intermediates using the lDDT metric, while PAthreader was unable to predict intermediates with complete three-dimensional structures. Therefore, on the benchmark data, the previous PAthreader cannot be compared with the FoldPAthreader because the output of the method is different. Based on your suggestions, We have added a description of the difference between the two methods on line 82 of manuscript.

**Figure R2**. The homepage of the web server.

line 82:

line 183:

[R1] Huang Z, Cui X, Xia Y, et.al. Pathfinder: Protein folding pathway prediction based on conformational sampling. *PLoS Comput Biol*. 2023;19(9):e1011438.

***Referee 1 mentions other metrics that could be used to predict early folding regions.***

**Response**: In the manuscript, we mentioned that protein folding order is related to structure conservation to some extent, and structure conservation can be reflected by the *F* value calculated from multiple structure alignments. Additionally, the hydrophobicity of protein can also be reflected to some extent by the proportion of buried residues. Therefore, here we analyzed the correlation of residue burial and *F* value with lDDT of early folded regions of predicted intermediate on 30 tested proteins. The results are shown in **Figure R3A, B**, their Pearson values are 0.203 and 0.577 respectively. This shows that both residue burial and *F* value have a certain correlation with lDDT of early folded region of predicted intermediates, although the correlation for residue burial is weak.

We further analyzed the proportion of buried residue and *F* value of early and late folded regions (**Figure R3C, D**). The results showed that 86.7% of proteins have a higher proportion of buried residues in early folded regions than in late folded regions, and 90% of proteins have a higher *F* value in early folded regions than in late folded regions. The results show that residue burial and *F* value are highly correlated with protein folding order, that is, buried residues and structurally conserved regions are preferentially formed in folding process. In the FoldPAthreader, residue burial and *F* value have been incorporated into the physical potential (*env* subterm) and statistical potential energy of the folding force field, respectively. We have added experimental analysis of the correlation of *F* values with folding order in the manuscript and supplementary information.

**Figure R3:** (A) Proportion of buried residues of early folded region in native structures versus lDDT of early folded region in predicted intermediates. (B) The average *F* value of early folded regions versus lDDT of early folded region in predicted intermediates. (C) The proportion of buried residues in early folded regions and late folded regions. (D) The average *F* value of early folded regions and late folded regions.

line 270:

***Referees 2 and 3 ask for a better description of the fragment library, and that you discuss limitations of the method.***

**Response**: We apologize for the unclear description. We have added descriptions to the manuscript based on suggestions of Referees 2 and 3. We calculated the average RMSD of 200 fragments corresponding to each residue position on the 30 test proteins. The results are shown in **Figure R4**, which shows the average RMSD of the 3-residue fragments and 6-residue fragments corresponding to EFR and LFR. On most successfully predicted proteins, the fragments corresponding to LFR has a higher RMSD than EFR. These results indicate that high *F* value regions are conserved and the derived fragments are similar, which can avoid exploring high-energy dead ends and facilitates the rapid assembly of this region. The fragments corresponding to low *F* value regions are diverse, making the low *F* value regions formed later in the prediction process. We have added these to line 193 of the manuscript as well as the supplementary information.

**Figure R4**. The average RMSD of 3-residue fragments and 6-residue fragments corresponding to EFR and LFR of 30 test proteins. Blue circles are targets successfully predicted by FoldPAthreader, yellow circles are failed targets.

In addition, based on suggestions of Referees 3, we added the prediction results using only 3-residues fragments, 6-residues fragments, and 9-residues fragments, as shown in **Table R2**. It can be seen that the performance of prediction using only 3-residues fragments is consistent with using both 3-residues and 6-residues fragments. But the TM-score of its final state is lower than that of 6-residues and 9-residues fragment. In contrast, although high-precision final state structures were obtained for 6-residues and 9-residues fragment, the number of predicted intermediates consistent with experimental data was lower than that 3-residues fragment. This is because MSTA-derived large fragments can assemble to form conformations faster than small fragments, accelerating the convergence of conformation, but they cannot explore conformations with diverse angles in small steps like 3-residues fragment. Therefore, conformational sampling through cooperative assembly of 3-residues and 6-residues fragment may be the optimal strategy in this method.

Finally, we have also added a discussion of limitations of this method to line 449 and the revisions has been highlighted in yellow in the manuscript.

**Table R2:** Results of 3-residues fragment and 6-residues fragment ablation experiments. Num is the number of proteins whose predicted intermediates are consistent with experimental data.

|  | Num (consistent with experimental data) | lDDT of early folded region | lDDT of late folded region | TM-score of final state |
| --- | --- | --- | --- | --- |
| use 3 and 6-fragment | 21/30 | 0.681 | 0.474 | 0.854 |
| use only 3-fragment | 21/30 | 0.674 | 0.499 | 0.826 |
| use only 6-fragment | 19/30 | 0.670 | 0.497 | 0.868 |
| use only 9-fragment | 17/30 | 0.695 | 0.511 | 0.877 |

line 115:

line 193:

line 449:

Reviewer #1

We very much appreciate for your comments and suggestions, which help to significantly improve the manuscript. Below we provide a point-by-point response to the comments, with all changes highlighted in yellow in the manuscript.

Zhao et al. present FoldPAthreader, a protein folding pathway prediction method that uses homologous structures and a Monte Carlo fragment assembly strategy. I think this paper will be of interest to the community provided that the following comments are addressed.

**Response**: Thank you for the nice summary and positive comments on our work. We have revised the manuscript as you suggested, as detailed below.

**# Major**

1. The 70% success number is mentioned in multiple places. How is it calculated? It could also be used in the ablation section, as it is not clear how to interpret Figure 7.

**Response**: We apologize for the unclear description. To objectively evaluate the consistency of protein folding order between the predicted results and biological experimental data, we quantitatively measured the predicted results by IDDT score. It is a scoring metric used to evaluate the local distance difference of atoms in the model, with larger values indicating greater structural similarity. The IDDT of the early folded region (EFR) and late folded region (LFR) were calculated by comparing the predicted intermediates with the native structure. When the lDDT of the early folded region of predicted intermediate is 10% higher than that of the late folded region, it means that the early folded region forms significantly more near-native contacts than the late folded region, indicating that the folding order is consistent with biological experimental data, that is, the prediction is successful. On the 30 target proteins, the IDDT of early folded region for 21 proteins was significantly higher than that of late folded region. Therefore, FoldPAthreader can successfully predict 70% of the proteins whose folding order is consistent with biological experimental data.

In the MSTA ablation test, the number of proteins successfully predicted by the component methods were 21, 17, 15 and 10, accounting for 70%, 56.7%, 50% and 33.3% of the test set respectively, indicating that FoldPAthreader performed best on AlphaFold DB50. Based on your suggestion, we have modified Figure 7 for a more intuitive understanding, as detailed below.

2. A number of quantities are mentioned that could also predict early folding regions (EFRs): sequence conservation, residue burial, hydrophobicity and the calculated F measure. These should be correlated or used to predict EFRs as baselines, otherwise there is a question about whether FoldPAthreader is superior to baseline methods for prediction.

**Response**: We thank you for your suggestions and apologize for the unclear description. In the manuscript, we mentioned that protein folding order is related to structure conservation to some extent, and structure conservation can be reflected by the *F* value calculated from multiple structure alignments. Additionally, the hydrophobicity of protein can also be reflected to some extent by the proportion of buried residues. Therefore, here we analyzed the correlation of residue burial and *F* value with lDDT of early folded regions of predicted intermediate on 30 tested proteins. The results are shown in **Figure R1A, B**, their Pearson values are 0.203 and 0.577 respectively. This shows that both residue burial and *F* value have a certain correlation with lDDT of early folded region of predicted intermediates, although the correlation for residue burial is weak.

We further analyzed the proportion of buried residue and *F* value of early and late folded regions (**Figure R1C, D**). The results showed that 86.7% of proteins have a higher proportion of buried residues in early folded regions than in late folded regions, and 90% of proteins have a higher *F* value in early folded regions than in late folded regions. The results show that residue burial and *F* value are highly correlated with protein folding order, that is, buried residues and structurally conserved regions are preferentially formed in folding process. In the FoldPAthreader, residue burial and *F* value have been incorporated into the physical potential (*env* subterm) and statistical potential energy of the folding force field, respectively. We have added experimental analysis of the correlation of *F* values with folding order in the manuscript and supplementary information.

**Figure R1:** (A) Proportion of buried residues of early folded region in native structures versus lDDT of early folded region in predicted intermediates. (B) The average *F* value of early folded regions versus lDDT of early folded region in predicted intermediates. (C) The proportion of buried residues in early folded regions and late folded regions. (D) The average *F* value of early folded regions and late folded regions.

line 270:

3. The link between evolutionary history and the folding pathway and its appearance within FoldPAthreader is not convincing. It seems sensible to guess that local structural variability exhibited in the structural alignments correlates inversely to earliness of folding (the above baseline would help test this), but it is not clear how this links to the claim in the introduction about recapitulation of evolutionary history during folding. Most proteins in the structural alignment will not be part of the evolutionary history of the target protein, though they may be homologous. This claim should be toned down or an example provided where the specific evolutionary history of a protein is linked to its folding with FoldPAthreader.

**Response**: Thank you for your suggestions. We fully agree with you that local structural variability exhibited in the structural alignments correlates inversely to earliness of folding, which has been verified by the above test results. For the link between protein folding process and evolutionary history, it was inspired by Ernst Haeckel's claim that ontogeny recapitulates phylogeny. But as you said, most of the proteins in the structural alignment may not be part of the evolutionary history of the target protein, which is currently difficult to prove with evidence. Therefore, we toned down this claim in lines 69, 198 and 442 of the manuscript. The revised description has been highlighted in yellow in the manuscript.

line 69:

line 198:

line 442:

4. The TMscore of 0.3 used to find structural homologs seems low and will bring in unlrelated folds. How was this number chosen and how is changing it expected to change the results?

**Response**: Thank you for this important question. When the TM-score is too high, the similarity and difference of local structures cannot be reflected in the structure alignment, making it difficult to capture structural conservation and folding information. When the TM-score is too low, irrelevant structures are included to the structure alignment, resulting in possible errors in folding information.

In this work, we selected structures with TM-score greater than or equal to a given threshold for multiple structure alignment. This threshold is determined through experiments, and the results are presented in **Table R1**. It can be seen that when the threshold is 0.3, the number of prediction results that consistent with the experimental data is the largest.

**Table R1:** Prediction results at different TM-score thresholds.

|  | num (consistent with experimental data) | lDDT of early folded region | lDDT of late folded region |
| --- | --- | --- | --- |
| TM-score = 0.1 | 18/30 | 0.672 | 0.473 |
| TM-score = 0.2 | 20/30 | 0.680 | 0.476 |
| TM-score = 0.3 | **21/30** | 0.681 | 0.474 |
| TM-score = 0.4 | 19/30 | 0.692 | 0.499 |
| TM-score = 0.5 | 16/30 | 0.695 | 0.514 |

\# Minor

5. The term folding force field is mentioned a few times, but it is not clear what it means so it could be clarified. Conventional MD force fields can fold proteins with only physical terms, at least in principle. It is not clear that the "proposed folding force field focuses on the folding process" any more than those. It is a physical and statistical force field with terms promoting native contacts based on variation in homologous structures. Perhaps it is the Monte Carlo sampling approach that makes it appropriate to study folding, but sampling is usually referred to as separate from the force field.

**Response**: We apologize for the unclear description. In this work, the proposed folding force field focuses on the **folding process** rather than the modeling of the final state, which is completely different from traditional modeling force fields such as I-TASSER and Rosetta. The folding force field is a composite force field composed of physical potential and statistical potential. In the physical potential, the hb_srbb, sheet and rsigma terms promote the formation of secondary structures in the early stages of folding, while the hs_pair, pair and env terms favor paired helices or sheet foldons. In the statistical potential, residues with a higher *F* value have a larger score weight, which can promote the formation of contact in the structurally conservative region earlier. By using different energy terms and weights, the three sampling stages are able to search for intermediate and transition states in the potential basin, rather than reaching the final state as quickly as possible. To avoid confusion for readers, we have added a description in line 231 of the manuscript.

line 231:

6. It is worth discussing how the F measure could be biased by the global structural alignment. For example, a two domain protein could align primarily on either domain with TMalign, with apparent larger distances in the non-aligned domain. Would it be possible to replace F with sequence conservation?

**Response**: We thank you for raising this important question. This work focuses on folding pathway prediction of single-domain proteins. The *F* values are calculated through global structural alignment, and structure with high *F* values tend to be more conservative. For multi-domain proteins, as you say, there is a larger distances in the non-aligned domain. Therefore, sequence conservation may be a feasible approach for multi-domain proteins, and we will consider it in future studies. Thank you again for your valuable suggestions.

7. Is there any correlation of Monte Carlo steps to experimental folding time? Do known fast folding proteins reach the native state faster?

**Response**: Based on your suggestion, we believe that there is a correlation between the Monte Carlo step and the experimental folding time, but the experimental folding time of the protein cannot be obtained from the current literature. Therefore, we analyzed the correlation of protein length with Monte Carlo running time. As shown in **Figure R2**, the results show that longer proteins have longer running times, which may be consistent with the folding of proteins in organisms.

**Figure R2:** The length of proteins versus the Monte Carlo running time.

8. Conventional MD successes such as the Shaw lab 12 proteins work could also be mentioned in the introduction.

**Response**: Thank you for point out this. According to your suggestion, we have added the description of Shaw lab 12 proteins work in the introduction.

line 45:

9. Figure 3: summary statistics could be shown, such as the mean values or a histogram/violin plot of the two distributions.

**Response**: Thank you for your suggestion. We have added the average lDDT of the 30 proteins at the end of the histogram in Figure 3C.

10. Figure 5: the Rg y-axes should be the same across the two plots. I also found the ordering of a-h to be confusing, these could increase left to right.

**Response**: We appreciate your careful review, which led us to discover the original error in the Rg y-axes, and we have correct this error. For the a-h conformations, they correspond to the order presented in of Figure 5B to facilitate visual comparison. Figure 5B is sourced from the report by Meng Qin et al [R1].

[R1] Qin, M., Wang, W.& Thirumalai, D. Protein folding guides disulfide bond formation. *Proceedings of the National Academy of Sciences of the United States of America* **112,** 11241-6 (2015).

11. Figure 6: the caption needs more detail, e.g. which protein is shown. It is not clear how the structures in A relate to those in B, there might be a better way to show this.

**Response**: We apologize for the confusion. We have added the description of Figure 6 in the caption. Figure 6A and B present the folding pathway of TIM predicted by FoldPAthreader and simulated by MD, respectively. It can be observed that intermediates *d-g* predicted by FoldPAthreader have similar structural characteristics to intermediate I_1A_ by MD, indicating that the folding pathway predicted by FoldPAthreader for TIM is consistent with the maximum probability pathway of MD (red arrow in Figure 6B).

Reviewer #2

We very much appreciate for your comments and suggestions, which help to significantly improve the manuscript. Below we provide a point-by-point response to the comments, with all changes highlighted in yellow in the manuscript.

In this work, the authors introduce FoldPAthreader, an interesting fold pathway prediction method that allows to investigate the potential folding pathway of a protein of interest. It builds on the recent advances in protein structural bioinformatics and addresses one of the major challenges of the field: how does one protein chain go from a disordered chain of amino acids to an ordered 3D fold. The method is conceptually interesting and of relevance for the field. The paper is written clearly but I have major comments, particularly related to unclarity (that in some cases may come across as over statements) and benchmarking. Please see my comments below:

**Response**: Thank you for the nice summary and positive comments on our work. We have revised the manuscript as you suggested, as detailed below.

Major:

- in the overview, some steps are not clear. For example, in line 101, it is not clear what the fragment library is, how it is generated and how it is used. It would be nice and clearer if the same level of detail given to the other features (eg, residue frequency score) is given here. This would help, for example, understand how they relate to the folding information used later. Actually, the fact that they are not explained well here, makes it that some later parts in the results and discussion are harder to interpret/understand.

**Response**: We apologize for the unclear description. We added a description of the fragment library and *F* value in the overview. Based on different distance deviation thresholds, *F* value is calculated from the MSTA, where a higher value indicates a higher frequency of residue alignment at corresponding positions of structures. The *F* value is combined with the residual distance information extracted from the predicted structure to further design the statistical potential energy function. Meanwhile, the candidate structures screened from MSTA are traversed, and continuous fragments of at least 6 residues and at least 3 residues are added to the fragment list in a dihedral angle representation, to generate a 6-residue fragment library and a 3-residue fragment library. The fragment libraries implicitly contain folding information and are specifically used for folding pathway prediction.

In addition, we also calculated the average RMSD of 200 fragments corresponding to each residue position on the 30 test proteins. The results are shown in **Figure R1**, which shows the average RMSD of the 3-residue fragments and 6-residue fragments corresponding to EFR and LFR. On most successfully predicted proteins, the fragments corresponding to LFR has a higher RMSD than EFR. These results indicate that high *F* value regions are conserved and the derived fragments are similar, which can bypass high-energy barrier and facilitates the rapid assembly of this region. The fragments corresponding to low *F* value regions are diverse, making the low *F* value regions formed later in the prediction process. We have added these to line 180 of the manuscript as well as the supplementary information.

**Figure R1**. The average RMSD of 3-residue fragments and 6-residue fragments corresponding to EFR and LFR of 30 test proteins. Blue circles are targets successfully predicted by FoldPAthreader, yellow circles are failed targets.

line 104:

line 193:

- In line 108, how different were the experimental setups? are the experimental conditions the same, similar or drastically different? could differences in experimental conditions explain the cases where FoldPAthreader did not preform well?

**Response**: Thank you for your suggestion. We investigated experimental methods for determining the folding pathways of 30 tested proteins from the available literature, and the results are shown in **Table R1**. The folding pathways of these proteins were determined using different experimental conditions such as hydrogen-deuterium exchange, circular dichroism or stopped-flow fluorescence experiments. It can be seen from the results that there may be no correlation between the performance of FoldPAthreader and the experimental methods. The difference in experimental conditions may explain the poor performance of FoldPAthreader, which need to be verified with more experimental data in future study.

**Table R1:** Experimental methods and predicted results of 30 test proteins.

| **PDB ID** | **Prediction** | **Experimental method** |
| --- | --- | --- |
| 1AIZ | success | stopped-flow fluorescence |
| 1AYI | success | stopped-flow fluorescence |
| 1BE9 | success | stopped-flow fluorescence + hydrogen-deuterium exchange |
| 1BGS | success | hydrogen-deuterium exchange |
| 1DIV | success | stopped-flow fluorescence |
| 1DKT | failure | stopped-flow fluorescence |
| 1E3Y | failure | stopped-flow circular dichroism + hydrogen-deuterium exchange |
| 1FTG | failure | hydrogen-deuterium exchange |
| 1HRH | failure | stopped-flow circular dichroism + hydrogen-deuterium exchange |
| 1I5T | success | hydrogen-deuterium exchange |
| 1J4H | success | hydrogen-deuterium exchange |
| 1MBC | failure | hydrogen-deuterium exchange |
| 1NTI | success | stopped-flow fluorescence |
| 1ONC | success | hydrogen-deuterium exchange |
| 1QLQ | success | hydrogen-deuterium exchange |
| 1SHF | success | hydrogen-deuterium exchange |
| 1STN | success | hydrogen-deuterium exchange |
| 1UBQ | failure | hydrogen-deuterium exchange |
| 1YYJ | success | hydrogen-deuterium exchange |
| 2CRT | success | stopped-flow fluorescence + hydrogen-deuterium exchange |
| 2F52 | success | stopped-flow fluorescence |
| 2KDI | success | circular dichroism + stopped-flow fluorescence |
| 2LZM | success | stopped-flow circular dichroism + hydrogen-deuterium exchange |
| 2TRX | success | stopped-flow fluorescence |
| 3BLG | failure | stopped-flow circular dichroism + hydrogen-deuterium exchange |
| 3CI2 | success | hydrogen-deuterium exchange |
| 3RNT | failure | hydrogen-deuterium exchange |
| 6LYZ | failure | stopped-flow fluorescence + circular dichroism |
| 7TIM | success | hydrogen-deuterium exchange |
| 9PCY | success | circular dichroism + hydrogen-deuterium exchange |

- In line 136, the lDDT of the different regions was computed using what as a reference? The target (AlphaFold) fold or the final fold generated by FoldPAthreader? If the latter, as the method generates an ensemble, which member of the ensemble was used? It is difficult to follow this paragraph, actually, as it is not clear at which state of the "simulation" these pLDDTs are being computed too. Please clarify this paragraph, it will further allow to better understand the work.

**Response**: We apologize for the unclear description. In this work, the native structure was used as a reference structure to calculate the lDDT of the early folding region and late folding region of the predicted intermediate. The intermediate is obtained by clustering the conformations generated from the folding nucleation stage. We have revised the description in the manuscript.

line 152:

- in line 166, the authors have to be careful when stating that the ensemble may reflect inherent flexibility without providing alternatives. I think what it actually reflects is the uncertantity of the method. There is no data provided by the the authors that supports that the ensemble is biologically relevant, which is a must to conclude that the ensemble may represent biologically relevant states.

**Response**: We thank the reviewer for pointing this out. Following your suggestion, we have removed the description in lines 189 and 439 that ensemble may reflecting inherent flexibility. This revision will improve the accuracy and clarity of the manuscript.

line 189:

line 439:

- in line 170, the authors make, in my opinion, a fallacious statement. They write that the results support the hypothesis. This would be true if the method to get the results would not be driven by the hypothesis. The authors developed a method that is fundamentally based on and driven by the hypothesis, so it is to be expected that the results follows it. I believe what the authors want to write is that the benchmark of the method indicates that the principles they based their method on can be applied to the test cases, giving support to the method. But they can not generalise it. Only 70% of the cases matched the results of the method and their dataset is small, not covering all possible paths proteins can follow to fold. So I would recommend the authors rephrase this part, clarifying it for the naive reader. This should also be addressed in the discussion, although the last part of the comment was already addressed.

**Response**: Thank you for bringing up this important point. We think your comments are reasonable, and we have toned down the description of the hypothesis in lines 69, 198 and 441 of the manuscript. We also made revisions to the discussion section and the revised description has been highlighted in yellow in the manuscript.

line 69:

line 198:

line 441:

- The authors often over-interpret the results of their method. One example is the point above about line 170. Another is in line 251, where the authors write that the results of their method indicate that there are different priorities for proteins of different secondary structure composition. Maybe that is the case but I don't think the authors provide enough supporting data. They are tacking this particular conclusion based solely on the results of the method and not benchmarking against experimental data (at least at this specific stage). In this line, I believe the maximum that they can conclude is that their method deals with different structure compositions differently. If this describes biologically relevant behaviour, that should be evaluated with some kind of benchmarking. The authors should make clear what they mean in this part, particularly when it can misinterpreted.

**Response**: Thank you for point out this. Based on your suggestion, we have toned down the description of the hypothesis in line 193 of the manuscript and removed the claim that the structural space of the known protein universe is complete. The revisions has been highlighted in yellow in the manuscript.

In line 251, it mainly explains the prediction results of our method through biologically relevant behaviors such as the impact of hydrogen bonding on folding, thereby verifying the effectiveness of the method. Based on your suggestions, we have reanalyzed the results in this section. In order to eliminate the error caused by the prediction method, we excluded 9 poorly predicted targets and analyzed the results for 21 proteins whose predicted folding pathways were consistent with experimental data. The average lDDT of the EFR for β-sheet, α-helix and α/β type protein intermediates are 0.778, 0.707 and 0.727, respectively, which are 32.4%, 24.7% and 28.9% higher than the LFR respectively. We have also reworded the description of this section, as detailed below.

line 193:

line 283:

- how does the method work with folding-affecting mutations? The authors refer in the intro that some point mutations can impair the folding pathway and promote aggregation, leading often to disease. But do not address this point in their results or the method, I would expect that the method is insensitive to point mutations that affect the folding, as it is based on AlphaFold models and patterns from MSAs. It would be important to address this issue, if not in the results by investigating the results of known variants (which can be easily obtained from UniProt) at least in the discussion.

**Response**: Thank you for your suggestion. As you pointed out, this method predicts protein folding pathways based on the AlphaFold models and the patterns from MSAs, and is insensitive to point mutations. In future research, folding-affecting mutations is an important problem for this method that needs to be further studied and verified. Based on your suggestion, we have added this description to the conclusions. folding-affecting mutations is an important problem that needs to be further studied and verified in future studies.

line 449:

- the authors show the predicted folding pathway for two wildly studied proteins, BPTI and TIM. These are not only widely studied from a folding perspective, but they are also quite widespread in nature and soluble. Therefore I would expect their MSAs to be quite deep and there are a lot of structures (predicted and experimental) for them. This raises the questions: (1) how does the method work for rare proteins that are predicted at high accuracy with AlphaFold? And (2) how does it deal with transmembrane proteins and those that require chaperones? These points should be addressed at least in the discussion.

**Response**: Thank you for your suggestions. We have counted the numbers of MSA and MSTA of BPTI and TIM, and they all exceeded 10,000, providing sufficient evolutionary information for prediction of protein folding pathways. Despite the promising folding pathway prediction results, the applicability and accuracy of FoldPAthreader could be further improved in several aspects. First, for rare proteins, there may not be enough homologs in the structural database, which will lead to reduced performance in folding pathway prediction. Second, this method predicts protein folding pathways based on the AlphaFold models and the patterns from MSAs, and is insensitive to point mutations. Third, protein folding pathways are also strongly affected by many cellular environmental factors. For example, molecular chaperones can interact with folding proteins to provide temporary structural support to prevent nonspecific interactions and aggregation. The dynamic nature of transmembrane proteins also makes it very challenging to determine the structure of their folding processes. Therefore, combining the characteristics of molecular chaperones and protein flexibility in protein folding pathway prediction methods may help improve the prediction accuracy.

Based on your suggestion, we have added descriptions to the conclusions and the revisions has been highlighted in yellow in the manuscript.

line 449:

- There is no benchmarking against other methods. The paper only covers the method described and does not compare it with other folding pathway prediction methods, which are mentioned in the introduction. In order to evaluate the quality and relevance of the method, it is important to have a point of comparison.

**Response**: Thank you for your suggestion. We added experiments to compare predicted folding pathway with Pathfinder on 30 test proteins. Pathfinder is a protein folding pathway prediction method that explores the transition probabilities of folding intermediate states through conformational sampling [R1]. It is difficult to compare with other methods because their code cannot be localized or cannot predict folding intermediates.

The results of FoldPAthreader and Pathfinder are shown in **Figure R2**. Red areas represents that the LDDT of the early folded region is 10%(green) higher than that of the late folded region. The cases in red areas indicate that the folding order is consistent with biological experimental data. On 30 proteins, FoldPAthreader successfully predicted 21 proteins whose intermediates were consistent with biological experimental data, and Pathfinder successfully predicted 12 proteins. The average IDDT of early folded region and late folded region are 0.681 and 0.474 for FoldPAthreader, and 0.568 and 0.479 for Pathfinder. These results show that the performance of FoldPAthreader is significantly better than that of Pathfinder. We have added these to line 183 of the manuscript and the supplementary information.

**Figure R2**. Head-to-head comparison between early folded region and late folded region of intermediates predicted by FoldPAthreader and Pathfinder. Blue circles are successfully predicted targets, yellow circles are failed targets.

[R1] Huang Z, Cui X, Xia Y, et.al. Pathfinder: Protein folding pathway prediction based on conformational sampling. *PLoS Comput Biol*. 2023;19(9):e1011438.

- The web resource is an easy way of using the method. I find it unclear in the main page which method is used when submitting a folding pathway prediction job. Is it FoldPAthreader or PAthreader? This is only clear after selecting the option to predict a folding pathway, but then it seems it is not possible to run PAthreader. It is also not clear from the main text how the two methods differ. It would be important to clarify this and include, at least, PAthreader in a benchmark against FoldPAthreader if possible.

**Response**: Thank you for point out this. Based on your suggestions, we have modified the homepage of the web server, as shown in **Figure R3**. It contains two modules: folding pathway prediction (FoldPAthreader) and remote template recognition (PAthreader), and they can both work normally.

PAthreader is a remote template recognition method that uses three-track alignment to thread PDB and AlphaFold DB libraries. Based on the identified remote homologs, PAthreader initially explores the folding order of protein using residue frequency distribution and secondary structure information. This process requires artificial thresholds to divide the folding order. Compared with PAthreader, FoldPAthreader extracts folding information from multiple structure alignments to construct a folding force field, and predicts folding pathway through conformational sampling, which does not require manual intervention. Furthermore, FoldPAthreader quantified the accuracy of folding intermediates using the lDDT metric, while PAthreader was unable to predict intermediates with complete three-dimensional structures. Therefore, on the benchmark data, the previous PAthreader cannot be compared with the FoldPAthreader because the output of the method is different. Based on your suggestions, We have added a description of the difference between the two methods on line 82 of manuscript.

**Figure R3**. The homepage of the web server.

line 82:

Minor:

- The authors write "The protein folding problem has emerged as a new challenge with the significant advances in deep learning driven protein structure prediction methods.", while this is relatively true, it is misleading. The protein folding problem is an old problem, it did not emerge now. What is unique to the current times is that with the protein structure prediction problem "solved" thanks to deep learning methods, it becomes now a reachable problem to assess using deep learning.

**Response**: Thank you for your suggestion. We fully agree with you on pointing out the historical context of the protein folding problem. It's indeed an old problem that has been a central challenge in molecular biology for decades. Based on your suggestion, we have revised the corresponding description in the abstract and kept it within 100 words in accordance with the genome biology Method article style.

- line 84: the authors write that the AlphaFold database is the most abundant structure database but earlier mention ESMfold database, which has 3x more models than AlphaFold DB. Why did the authors not use ESMfold database?

**Response**: Thank you for your suggestion. The accuracy of structure predicted by AlphaFold2 using MSA is higher than that of ESMfold, resulting in more high-quality structures in AlphaFold DB. Furthermore, it can be seen from the results of database ablation experiments that FoldPAthreader performs best on AlphaFold DB50 because it provides more diverse structural information. Although AlphaFold DB has more structures, they are extremely identical and redundant, resulting in relatively little available folding information. In future development of FoldPAthreader, we will conduct large-scale data analysis and consider using the ESMfold database.

- in line 176, could the authors write Haeckel's statement here too and not only reference it? It would facilitate reading this paragraph.

- in line 178, "the evolutionary" should be "the evolution" or "the evolutionary history"

**Response**: Thank you for point out this. Based on your suggestion, we removed Haeckel's statement in this section and toned down the description of the hypotheses in the manuscript. "the evolutionary" has been revised to "the evolution". Thank you again for your suggestions, and we believe that these improvements will make it easier for readers to understand.

line 202:

- in line 195, why TM-score > 0.3? Anything below 0.5 is not a significant similarity...

**Response**: We apologize for the confusion. There are some irrelevant structures among the homologs searched by Foldseek, so structures with TM-score less than the threshold need to be excluded. In this work, we selected structures with TM-score ≥ 0.3 for multiple structure alignment. This threshold is determined through parameter experiments, and the results are presented in **Table R2**. It can be seen that when the threshold is 0.3, the number of prediction results that consistent with the experimental data is the largest. When the threshold is 0.5, the structures are too similar to the target protein in the globle topology, which will lead to the loss of structural conservation information in multiple structures alignment.

**Table R2:** Prediction results at different TM-score thresholds.

|  | num (consistent with experimental data) | lDDT of early folded region | lDDT of late folded region |
| --- | --- | --- | --- |
| TM-score = 0.1 | 18/30 | 0.672 | 0.473 |
| TM-score = 0.2 | 20/30 | 0.680 | 0.476 |
| TM-score = 0.3 | **21/30** | 0.681 | 0.474 |
| TM-score = 0.4 | 19/30 | 0.692 | 0.499 |
| TM-score = 0.5 | 16/30 | 0.695 | 0.514 |

- this is just a very minor comment, and is completely up to taste, but I find the animation of the PAthreader logo in the web resource distracting.

**Response**: Thank you for point out this and we have modified PAthreader logo, as shown in **Figure R4**.

**Figure R4**. The homepage of the web server.

Reviewer #3

We very much appreciate for your comments and suggestions, which help to significantly improve the manuscript. Below we provide a point-by-point response to the comments, with all changes highlighted in yellow in the manuscript.

The question addressed in this work is with no doubt of high interest. The method is interpretable. Moreover, it opens very many questions on how to go further with respect to what the authors do, at large scale.

**Response**: Thank you for the nice summary and positive comments on our work. We have revised the manuscript as you suggested, as detailed below.

The idea is simple. The authors leverage on existing AF2 structures to produce a library of protein fragments, a physical potential energy function and a statistical potential energy function leading to the reconstruction of the folding pathway.

Their hypothesis is that the conformational changes that proteins undergo when they fold from a disordered state to a specific three-dimensional structure reflect the evolutionary history of the proteins and it should be decoded from sequences. This idea is not new but it is used here in a very successful way. Namely, it reminds me of the usage of coevolution analysis to explain "how coevolving clusters of amino-acids can help to identify stable subparts of the protein sequence in the folding process" for protein A discussed in (Dib, L., & Carbone, A. (2012). Protein fragments: functional and structural roles of their coevolution networks. PLoS One, 7(11), e48124). I cite especially this work because, crossed with what the authors do, coevolution analysis might help them to go further towards a large-scale development of their method.

**Response**: We thank the reviewers for this insightful comments. Since 2012, deep learning technology has been gradually applied to coevolution analysis. The method of coevolution analysis based on multiple sequence alignment (MSA) provides more reliable and accurate clues for protein structure prediction. In this work, we exploited the evolutionary history of proteins through multiple structure alignment (MSTA) to extract protein folding information, and then applied it to the prediction of protein folding pathway. In the future, combining deep learning techniques with coevolution analysis methods may contribute to the large-scale development of our method. We have added relevant references and cited them appropriately.

line 208:

The method has been tested on 30 known structures. The ensemble of proteins is limited because experimental data on folding pathways reconstruction is also limited. This dataset is definitely biased in terms of the stability of the structures, the lack of long/short unstructured loops, intrinsically disordered regions, and regions that undergo disorder-to-order transitions after complexification. Having said this, it is reasonable to start from well-structured proteins and the authors should just discuss explicitly this point in their article instead of proposing the method as resolving a general problem. In my opinion this method has the clear merit to advance the question but it is not the final work on the question when large-scale prediction has to be approached.

**Response**: Thank you for point out this. Based on your suggestions, we have revised the description of the dataset in the manuscript. For the problem of large-scale prediction, it may be caused by our inappropriate description. As you said, our method helps advance the problem of protein folding pathway prediction, but it still needs improvement to be applied to large-scale protein folding pathway prediction. We have revised the corresponding description in the manuscript. We believe that your comments will play a great role in our follow-up work. Thank you again.

line 128:

line 391:

Inaccurate sentences in the abstract should be changed:

Line 10 - "While the structures of almost all known proteins have been successfully predicted, the process by which they fold remains an enigma"

Here we do not speak about "Known proteins" but about "proteins known to have a structure". The large amount of disordered protein sequences is neglected by the interests of this method.

**Response**: We apologize for the inaccurate description. Based on your suggestions, we have revised the abstract of the manuscript and kept it within 100 words in accordance with the genome biology Method article style.

Line 15: the authors use the word "folding mechanism" but I do not see any mechanism that is unraveled in this article. Do they want to speak about "folding pathways"?

This wording should be systematically replaced in the full manuscript. If I miss something, this means that they should addressed explicitly the idea of "mechanism" in a convincing way.

**Response**: We are in full agreement with your advices. we have replaced and modified "folding mechanism" in the manuscript.

In the background section, line 43, the authors mention molecular dynamics. There is a recent work on Alpha Flow and ESMFlow (https://openreview.net/pdf?id=yQcebEgQfH ; Jing, B., Berger, B., & Jaakkola, T. (2024). AlphaFold Meets Flow Matching for Generating Protein Ensembles. arXiv preprint arXiv:2402.04845) that should be referred to in the introduction or in the discussion. Again, this work can be useful to the authors to go further towards a large-scale development of their method.

**Response**: Thank you for your suggestion. AlphaFLOW/ESMFLOW are flow-based generative modeling methods for learning and sampling the conformational landscape of proteins. This method can be used in place of expensive simulations to diversify and obtain equilibrium ensembles of solved protein structures. It will be useful for further development of our method in future study. Based on your suggestion, we have added descriptions to the introduction section.

line 49:

Line 87 - Figure 1. The labels U, TS, I, N are not defined. I guess they stand for Transition state (TS) and intermediate (I) states. Please define the four states in the text.

**Response**: Thank you for point out this. The fact is such. We have defined them in the legend to Figure 1 of the manuscript.

Line 130. The author say "It is observed that the aligned structures of the folding intermediates ensemble are consistent with preferentially folded blue structures…". You need to use quantitative measures. Please replace this sentence with quantitative evidence.

Line 134. "the results suggest that FoldPAthreader has the ability to predict the folding pathway for general proteins, and is not limited to the type and size of proteins.". as mentioned above, the size of the protein is not the only important feature to evaluate difficulties in the folding pathway reconstruction. Disordered regions, loop structures, weakly stable regions are also potential components. The authors should provide support for this statement with much more precision. This effort will establish the limits of the analysis. As mentioned setting these limits will provide a clear view on the work.

**Response**: Thank you for your suggestion. We have removed this sentence and revised the related description. To maintain logical coherence, we describe the quantitative evidence in line 177. This revision will improve the accuracy and clarity of the manuscript.

For protein size, as you say, it is not the only important feature to evaluate difficulties in the folding pathway reconstruction. We have performed a detailed analysis of the results for different protein types (β-sheet, α-helix and α/β type) in lines 283-300 and have made modifications to the original description, as detailed below.

line 148:

line 177:

lines 283-300:

Line 146. It would be interesting, and fundamental I guess, to have some evaluation of the variability on the fragments in the library which are used to determine the folding pathways of a protein. How much variability on these fragments exist for pathways that are successfully predicted? We might have the impression that successful predictions are linked to a restricted space of fragment conformations, leading the dynamics. Is it the case?

**Response**: Thank you for your suggestion. Indeed, successful predictions are related to the restricted space of fragment conformations. MSTA-derived folding fragment libraries contribute to accelerating the preferential formation of early folded region. We calculated the average RMSD of 200 fragments corresponding to each residue position on the 30 test proteins. The results are shown in **Figure R1**, which shows the average RMSD of the 3-residue and 6-residue fragments corresponding to early folded regions (EFR) and late folded regions (LFR). On most successfully predicted proteins, the fragments corresponding to LFR has a higher RMSD than EFR. These results indicate that high *F* value regions are conserved and the derived fragments are similar, which can avoid exploring high-energy dead ends and facilitates the rapid assembly of this region. The fragments corresponding to low *F* value regions are diverse, making the low *F* value regions formed later in the prediction process. We have added these to line 193 of the manuscript as well as the supplementary information.

**Figure R1**. The average RMSD of 3-residue fragments and 6-residue fragments corresponding to EFR and LFR of 30 test proteins. Blue circles are targets successfully predicted by FoldPAthreader, yellow circles are failed targets.

line 193:

Line 155. Figure 2. It would be interesting to have a color scale in the predictions that fits the time of formation of the structure, as given for the experimental folding order with blue-grey colors. Could you please replace the present rainbow scale colors with something meaningful to this work? Folding order is what I would expect to see.

As a general principle, please add color scale bars to your figures and explain them in the legends.

Line 155. Figure 2. You say "Their partial structures with high overlap are marked with β-sheet or α-helix, indicating preferential formation." How the overlapping is quantitatively defined? High vs low? Can you be more quantitative?

**Response**: This is a good suggestion and we apologize for the unclear description. The structural overlap of the intermediates ensemble in Figure 2 was obtained by selecting the three secondary structure fragments with the highest *F* value for structure alignment. Based on your suggestion, we have modified Figure 2 by replacing the rainbow scale colors with red-white-blue and adding color scale bars. The color blue indicates high overlap in the predicted intermediate ensemble, suggesting that folding occurs preferentially during the prediction process. Conversely, the color red indicates a low overlap, suggesting that folding occurs later in the prediction process.

We have added color scale bars to the Figure 2 and explained them in the legend. Red to blue are encoded by the average RMSDnorm of the residues of the intermediate ensemble. The range of RMSDnorm is 0-1, which uses a method similar to TM-score to normalize RMSD. It is beneficial to color coding, defined as follows:

RMSDnorm= 11+did02

d0=1.243L-15-1.8

where di is the distance between the *i*-th pair of residues. d0 is a scale to normalize the match difference, which comes from the definition of TM-score [R1]. L is the length of the protein. We have added these to the supplementary information (Text S1).

[R1] Zhang Y, Skolnick J. Scoring function for automated assessment of protein structure template quality. *Proteins*. 2004;57(4):702-710.

Line 160. Figure 3B. are the blue square defined wrt existing secondary structures? How are they identified?

Figure 3C. please provide the intuition on small and large differences in IDDT for early/late folded regions. To what is it due? Is IDDT really a good/the right measure to discriminate difficult situations?

**Response**: We apologize for the unclear description. We modified Figure 3B to provide intuition for small and large differences in IDDT for early folded regions (EFR) and late folded regions (LFR). Figure 3B is a distance difference map between the native state and predicted intermediate of CTX III (residue pairs within 15 Å). The blue box corresponds to the distance difference map between the triple-stranded β-sheet of native state and that of predicted intermediate, which are the early folded region. The secondary structure was calculated from the native structure using DSSP program. The purpose of this figure is to illustrate how lDDT is calculated for EFR and LFR. For the predicted intermediate of CTX III (Figure 3A), its triple-stranded β-sheet has a similar structure to the EFR of native state, indicating that the triple-stranded β-sheet folds preferentially during the prediction process. Its IDDT is 0.703, calculated based on the residue pairs in the blue box in Figure 3B, which is significantly higher than the IDDT of LFR (0.419).

The lDDT is a superposition-free score that evaluates local distance differences of atoms in the model. The interaction distances (cutoff 15 Å) between atoms in the reference structure are compared with distances between corresponding atoms in the predictions. If the difference between the two distances is within a defined threshold, the interaction is considered to be preserved in the prediction. The final lDDT-all score is computed by averaging the fraction of correctly modeled interactions for the following four distance difference thresholds: 0.5, 1, 2, and 4 Å. When the lDDT of the EFR of predicted intermediate is higher than that of the LFR, it means that the EFR forms more near-native contacts than the LFR. Therefore, we think that IDDT is a suitable metric for evaluating predicted folding intermediates in this work.

Line 172. The fact that the structural space of the known protein universe can be used to study most protein folding pathways is an important point that is made by this work. The authors state the idea as "the structural space of the known protein universe is complete and can be used to study most protein folding mechanisms". As mentioned above they cannot speak about mechanisms and they cannot claim that the space is "complete". They do not prove this.

**Response**: Thank you for point out this. Based on your suggestion, we have toned down the description of the hypothesis in line 193 of the manuscript and removed the claim that the structural space of the known protein universe is complete. The revisions has been highlighted in yellow in the manuscript.

line 193:

Line 168. The authors speak about fragments but I had to go in the Methods to discover that their length if either 6- or 3-aa in length. It would be better to address their characteristics from this point in the manuscript. Moreover, I found that more statistics should be provided about fragments. Are they fragments that are found more than others in structures? What is their aa composition? …. There are a number of important questions that can be addressed on them to provide some intuition on their role.

**Response**: We apologize for the unclear description. Based on your suggestion, we have added the description of how fragment libraries are generated to the overview on line 115. The fragment libraries are generated by sequentially traversing the candidate structures filtered from MSTA. Continuous fragments of at least 6 residues and at least 3 residues are added to the fragment list in a dihedral angle representation, to generate a 6-residue fragment library and a 3-residue fragment library.

We have also added statistics for the fragment library in line 193 of the manuscript and supplementary information. The average RMSD of 200 fragments corresponding to each residue position on the 30 test proteins was calculated. The results are shown in **Figure R2**, which shows the average RMSD of the 3-residue and 6-residue fragments corresponding to early folded regions (EFR) and late folded regions (LFR). On most successfully predicted proteins, the fragments corresponding to LFR has a higher RMSD than EFR. These results indicate that high *F* value regions are conserved and the derived fragments are similar, which can avoid exploring high-energy dead ends and facilitates the rapid assembly of this region. The fragments corresponding to low *F* value regions are diverse, making the low *F* value regions formed later in the prediction process.

**Figure R2**. The average RMSD of 3-residue fragments and 6-residue fragments corresponding to EFR and LFR of 30 test proteins. Blue circles are targets successfully predicted by FoldPAthreader, yellow circles are failed targets.

line 115:

line 193:

About their length. Why 6 and 3? Is there some meaning about the role that fragments might have compared to 6-fragments?

Can you add in table S4 two lines reporting the performance with the 6-fragment library only and the 3-fragment library only?

**Response**: We apologize for the confusion. In the initialization stage of prediction, the conformation is initialized by random 20*L times of 3-residues fragment assembly. In the folding nucleation stage, the trial conformations in the first half of the generations were generated by 6-residues fragment assembly, and in the second half of the generations using 3-residues fragment assembly. In the structure finalization stage, the generation of trial conformations follows the same process as in the folding nucleation stage.

Based on your suggestion, we added the prediction results using only 3-residues fragments, 6-residues fragments, and 9-residues fragments, as shown in **Table R1**. It can be seen that the performance of prediction using only 3-residues fragments is consistent with using both 3-residues and 6-residues fragments. But the TM-score of its final state is lower than that of 6-residues and 9-residues fragment. In contrast, although high-precision final state structures were obtained for 6-residues and 9-residues fragment, the number of predicted intermediates consistent with experimental data was lower than that 3-residues fragment. This is because MSTA-derived large fragments can assemble to form conformations faster than small fragments, accelerating the convergence of conformation, but they cannot explore conformations with diverse angles in small steps like 3-residues fragment. Therefore, conformational sampling through cooperative assembly of 3-residues and 6-residues fragment may be the optimal strategy in this method. We have added these to the manuscript and the supplementary information.

**Table R1:** Results of 3-residues fragment and 6-residues fragment ablation experiments. Num is the number of proteins whose predicted intermediates are consistent with experimental data.

|  | Num (consistent with experimental data) | lDDT of early folded region | lDDT of late folded region | TM-score of final state |
| --- | --- | --- | --- | --- |
| use 3 and 6-fragment | 21/30 | 0.681 | 0.474 | 0.854 |
| use only 3-fragment | 21/30 | 0.674 | 0.499 | 0.826 |
| use only 6-fragment | 19/30 | 0.670 | 0.497 | 0.868 |
| use only 9-fragment | 17/30 | 0.695 | 0.511 | 0.877 |

line 115:

There is a lot of work done on structural alphabets, from more than 20 years now and there is no mention of these works in the article. They should be mentioned and discussed in the light of the differences introduced here:

Camproux, A. C., Brevern, A. G., Hazout, S., & Tufféry, P. (2001). Exploring the use of a structural alphabet for structural prediction of protein loops. Theoretical Chemistry Accounts, 106, 28-35.

de Brevern, A., Camproux, A. C., Hazout, S. A., Etchebest, C., & Tufféry, P. (2001). Protein structural alphabets: beyond the secondary structure description. Recent research developments in protein engineering, 319-331.

Etchebest, C., Benros, C., Hazout, S., & de Brevern, A. G. (2005). A structural alphabet for local protein structures: improved prediction methods. Proteins: Structure, Function, and Bioinformatics, 59(4), 810-827.

Tyagi, M., De Brevern, A. G., Srinivasan, N., & Offmann, B. (2008). Protein structure mining using a structural alphabet. Proteins: Structure, Function, and Bioinformatics, 71(2), 920-937.

Pandini, A., Fornili, A., & Kleinjung, J. (2010). Structural alphabets derived from attractors in conformational space. BMC bioinformatics, 11, 1-18.

Craveur, P., Joseph, A. P., Esque, J., Narwani, T. J., Noël, F., Shinada, N., ... & de Brevern, A. G. (2015). Protein flexibility in the light of structural alphabets. Frontiers in molecular biosciences, 2, 20.

**Response**: We thank you for your suggestion, which made us realize that the structural alphabet is a very meaningful research. Proteins exhibit a hierarchical structure, with primary sequences of amino acids folding into secondary structures, which further fold into tertiary structures. This process often results in the repetition or reoccurrence of certain structural motifs or conformations within different proteins. To efficiently capture these local conformations, structural alphabets were proposed. These alphabets represent the most typical or frequently occurring local conformations observed in protein structures. By using structural alphabets, researchers can simplify the complex structural information of proteins into a manageable set of representative motifs or states. This simplification aids in structural analysis, protein folding research, and understanding the relationships between sequence, structure, and function of proteins. Based on your suggestion, we have added a description of the structural alphabets to line 73 and added relevant references.

line 73:

Important: The fragment library should be made available. It is not clear if it is.

**Response**: Thank you for your suggestion. We have added the fragment library to the server's prediction results, and users can click on results to download the results file.

Line 175. Big claims are made here. The idea is then proved on a single example + 4 more! The text should be rephrased.

**Response**: Thank you for point out this. We have revised this section and toned down the description of the hypotheses in the manuscript. In this section, we use the case of plastocyanin to intuitively analyze the relationship between protein evolution and folding. The diversity of protein structures observed within protein families is a result of evolutionary processes driven by functional selection, which reflect the evolutionary history of protein families to some extent.

In addition, we also added experiments to analyze the correlation between *F* values and lDDT of early folded region of predicted intermediates (**Figure R3A**) as well as the comparison of average *F* values of early folded region and late folded region (**Figure R3B**) on 30 tested proteins. The results show that there is a certain correlation between *F* value and lDDT of early folded region (Pearson *r* = 0.577), and 90% of the proteins have a higher *F* value in the early folded region than in the late folded region. These suggest that conserved evolutionary regions may be preferentially formed during the folding process. We have added the experiment to this section and the supplementary information.

**Figure R3:** (A) The average *F* value of early folded regions versus lDDT of early folded region in predicted intermediates. (B) The average *F* value of early folded regions and late folded regions.

line 203:

line 270:

Generally, there are limitations of the method that are not enough discussed. For instance:

- What is its dependence on the size of the protein, its conservation in the tree of life?

- Can the method predict folding pathways for new proteins?

- What is its performance on flexible regions? Compared to more conserved ones?

- does the method observe situations where unfolded parts undergo states of disorder-to-order-to-disorder transitions in the dynamics they reconstruct?

**Response**: We appreciate for your suggestions. Below, we have provided point-by-point replies to the comments. We hope that the following experiments and discussions respond to your questions.

*-What is its dependence on the size of the protein, its conservation in the tree of life?*

**Response**: First, the method is not theoretically limited by the size of the protein. We analyzed the correlation of protein length with Monte Carlo running time. As shown in **Figure R4**, the results show that longer proteins have longer running times. Due to limited computing resources, we recommend users to submit proteins with sequence lengths less than 300 on the online server.

Second, during the evolution, proteins may undergo structural conservative or innovative changes. Therefore, proteins can be divided into many families and subfamilies based on their structure and function. Protein evolutionary trees can show the origins and evolutionary pathways of different protein families, providing important clues about the evolutionary history of organisms. We have provided relevant descriptions in line 203 of the manuscript.

**Figure R4:** The length of proteins versus the Monte Carlo running time.

line 203:

*-Can the method predict folding pathways for new proteins?*

**Response**: This method can predict the folding pathways of new proteins because its input is only the amino acid sequence. We predicted the folding pathways of three human proteins whose structures have not been solved experimentally, and the results are shown in **Figure R5**. These results have yet to be verified by biological experiments.

**Figure R5:** The yellow structures are predicted by AlphaFold2 based on the sequence. The red-white-blue structures are the intermediate ensembles predicted by FoldPAthreader. The color blue indicates high overlap in the predicted intermediate ensemble, suggesting that folding occurs preferentially during the prediction process. The color red indicates a low overlap, suggesting that folding occurs later.

*-What is its performance on flexible regions? Compared to more conserved ones?*

**Response**: We searched the two-state structures of 30 test proteins in the PDBflex library [R2], and calculated the distance of the corresponding residues by optimally overlap of the two states. When the distance is greater than 1 Å, it was defined as a flexible residue. Finally, four proteins with more than 5 flexible residues were selected for folding pathway prediction analysis, and the results are shown in **Figure R6.** For the Rd-apocytochrome b562 and Acyl-CoA binding protein, the flexible region is contained in the late folded region, while the flexible region of the HIV-1 ribonuclease H and CTX III is in both the early folded and late folded regions. These may indicate that there some correlation between the protein flexibility and folding pathway. However, this conclusion may not be correct because it is difficult to draw firm conclusions from limited data. Therefore, a more detailed study of the relationship between protein flexibility and folding mechanisms is crucial for a comprehensive understanding of protein folding dynamics in the future. Future research might involve experimental techniques and computational simulations to explore how flexibility impacts folding pathways.

**Figure R6:** (A) The blue-grey structure is an annotated folding order in the native state. The blue regions are first folded, followed by the gray. (B) The red-white-blue structures are the intermediate ensembles predicted by FoldPAthreader. (C) Yellow and green are the two-state structures of the protein, and red is the flexible region.

[R2] Hrabe T, Li Z, Sedova M, et al. PDBFlex: exploring flexibility in protein structures. Nucleic acids research, 2016, 44(D1): D423-D428.

*-does the method observe situations where unfolded parts undergo states of disorder-to-order-to-disorder transitions in the dynamics they reconstruct?*

**Response**: On the test set, we observed that some proteins undergo a disorder-to-order-to-disorder transition during the prediction process. **Figure R7** shows the radius of gyration of the BPTI protein, which gradually decreases from the initial conformation to the final state. The conformation temporarily falls into a local basin during the folding process, i.e. conformation *c*, which indicates that BPTI undergoes a state of disorder-to-order transitions in the early stages. As the conformation jumps out of the basin, it undergoes a state of order-to-disorder transitions. Finally, it folds into an ordered final state.

**2^nd^ round**

**Reviewer 1**

The authors have addressed the comments well

**Reviewer 2**

The authors addressed all my concerns satisfactorily. The manuscript is very much improved

**Reviewer 3**

I appreciate the revisions made by the authors, which have substantially addressed my previous concerns. However, there are a few additional modifications that could further enhance the manuscript and user experience.

Firstly, it would be beneficial to include information regarding the expected computational time in the manuscript and in the server. For instance, when I submitted a protein sequence of approximately 200 amino acids, the process lasted about 9 hours. Providing users with an estimated wait time could help set appropriate expectations.

Additionally, I encountered an issue with the "folding pathway" screen; the intended movie did not display on the website, though I was able to view it locally since the data were provided. Addressing this bug could significantly improve the functionality of the server.

Lastly, there appears to be a discrepancy regarding the maximum protein length acceptable for analysis, which is currently capped at 300 amino acids. The server mentions a "possible" threshold, yet does not accept sequences exceeding this limit. Clarifying this limitation both in the manuscript and on the server would provide clearer guidance for users.

Thank you for considering these points. I look forward to the final version of the paper.

**Authors’ response**

I appreciate the revisions made by the authors, which have substantially addressed my previous concerns. However, there are a few additional modifications that could further enhance the manuscript and user experience.
**Response**: We very much appreciate for your suggestions, which help to improve the manuscript and server. Below we provide a point-by-point response to the comments

Firstly, it would be beneficial to include information regarding the expected computational time in the manuscript and in the server. For instance, when I submitted a protein sequence of approximately 200 amino acids, the process lasted about 9 hours. Providing users with an estimated wait time could help set appropriate expectations.
**Response**: Thank you for your suggestions. We analyzed the correlation of protein length with running time on benchmark. As shown in Figure R1, the results show that longer proteins have longer running times. According to your suggestion, we provide an estimated wait time on the submission page and in the email (Figure R2).

**Figure R1:** The length of proteins versus the running time.

**Figure R2**. The submission page of the web server.

Additionally, I encountered an issue with the "folding pathway" screen; the intended movie did not display on the website, though I was able to view it locally since the data were provided. Addressing this bug could significantly improve the functionality of the server.
**Response**: We apologize for this. It's caused by larger movie files that take too long to render. When loading fails, the user can refresh the web page and reload it. We have added instructions to the results page, as show in Figure R3.

**Figure R3**. The results page of the web server.

Lastly, there appears to be a discrepancy regarding the maximum protein length acceptable for analysis, which is currently capped at 300 amino acids. The server mentions a "possible" threshold, yet does not accept sequences exceeding this limit. Clarifying this limitation both in the manuscript and on the server would provide clearer guidance for users.
**Response**: Thank you for your suggestions. The method is not theoretically limited by the size of the protein. Since computing resources are limited during the CASP16 competition, we only allow users to submit targets with an amino acid length less than 300. We have clarified this limitation on the server, as show in Figure R4.

**Figure R4**. The homepage of the FoldPAthreader server.
